# How to Tell an N from an O: Controlling the Chemoselectivity of Methyltransferases

**DOI:** 10.1021/acscatal.5c00834

**Published:** 2025-04-04

**Authors:** Emely Jockmann, Helena Girame, Wieland Steinchen, Kalle Kind, Gert Bange, Kai Tittmann, Michael Müller, Ferran Feixas, Marc Garcia-Borràs, Jennifer N. Andexer

**Affiliations:** † Institute of Pharmaceutical Sciences, University of Freiburg, Albertstr. 25, 79104 Freiburg, Germany; ‡ Institut de Química Computacional i Catàlisi and Departament de Química, Universitat de Girona, C/ Maria Aurèlia Capmany, 69, 17003 Girona, Spain; § Center for Synthetic Microbiology, Philipps University Marburg, Karl-von-Frisch-Str. 14, 35043 Marburg, Germany; ∥ Department of Chemistry, Philipps University Marburg, Hans-Meerwein-Str. 4, 35043 Marburg, Germany; ⊥ Schwann-Schleiden-Forschungszentrum—Department of Molecular Enzymology, Georg-August-Universität Göttingen, Julia-Lermontowa-Weg 3, 37077 Göttingen, Germany

**Keywords:** anthranilate *N*-methyltransferase, biocatalysis, caffeate *O*-methyltransferase, chemoselectivity, open/closed conformational dynamics

## Abstract

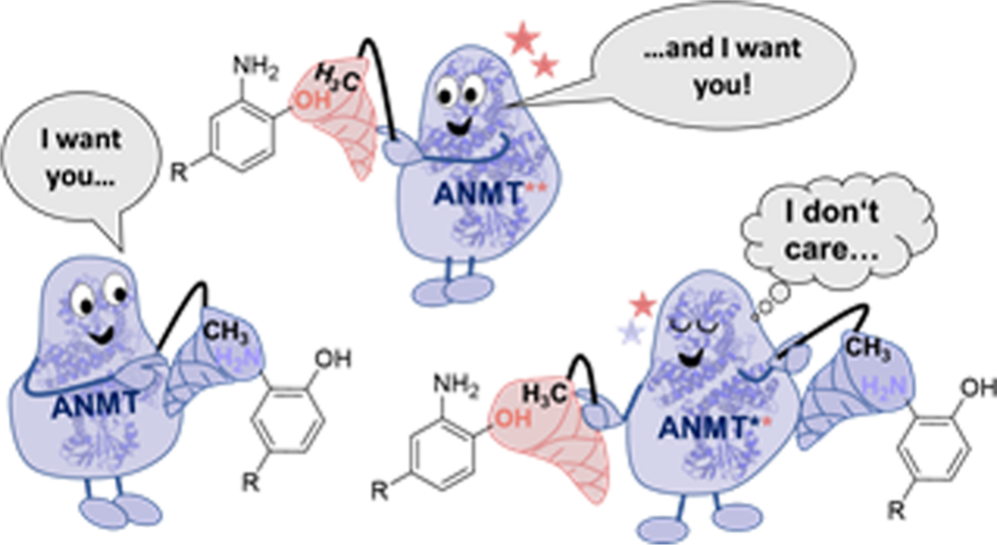

*S*-Adenosyl-l-methionine (SAM)-dependent methyltransferases (MTs) are important enzymes in numerous biological pathways. They share a common S_*N*_2 mechanism but act on different nucleophilic substrates in vivo. Therefore, MTs have a specific chemoselectivity to transfer CH_3_ onto the correct atom type and substrate. Caffeate *O*-MT from *Prunus persica* (*Pp*CaOMT) and anthranilate *N*-MT from *Ruta graveolens* (*Rg*ANMT) share a high similarity regarding their amino acid sequence (>74%). Nevertheless, the physiological substrates (caffeate vs anthranilate) and attacking nucleophiles (hydroxyl vs amino group) are strikingly different. We demonstrate that the differing chemoselectivity is governed by different conformational states of the two enzymes. *O*-Methylation catalyzed by CaOMTs requires a “closed” conformation, whereas ANMTs perform *N*-methylation in an “open” state. We rationally designed seven variants for both *Pp*CaOMT and *Rg*ANMT, which changed their original nucleophile preference to different extents, up to a full inversion. Interestingly, the generated *O*-selective ANMT variant catalyzes *O*-methylation considerably faster than wildtype CaOMT. Molecular dynamics (MD) simulations and hydrogen/deuterium exchange mass spectrometry (HDX-MS) experiments showed that the mutations induced changes in the conformational dynamics of the enzyme variants and by modulating the open/closed transitions impact the corresponding chemoselectivity. Our data show that the selectivity of the methyl transfer reaction is not solely governed by the key residues directly involved in the methyl transfer but is rather synergistically modulated by the conformational dynamics of the enzyme and reaction conditions.

## Introduction

Enzymes are intrinsically stereo-, regio-, and chemoselective catalysts. The broad range of methods in enzyme research such as directed evolution, and computational approaches including AlphaFold or molecular dynamics (MD) simulations, have contributed to gaining more knowledge about the structure and function of enzymes at the molecular level.^1−3^ Based on this, enzymes can be tailored to specific target substrates and reactions. While the adaptation of an enzyme to, e.g., larger residues or functional groups is often comparatively easy to accomplish, switching or changing selectivities (such as stereo- or chemoselectivity) is much more difficult and less straightforward to predict.^2−6^

**Figure 1:**
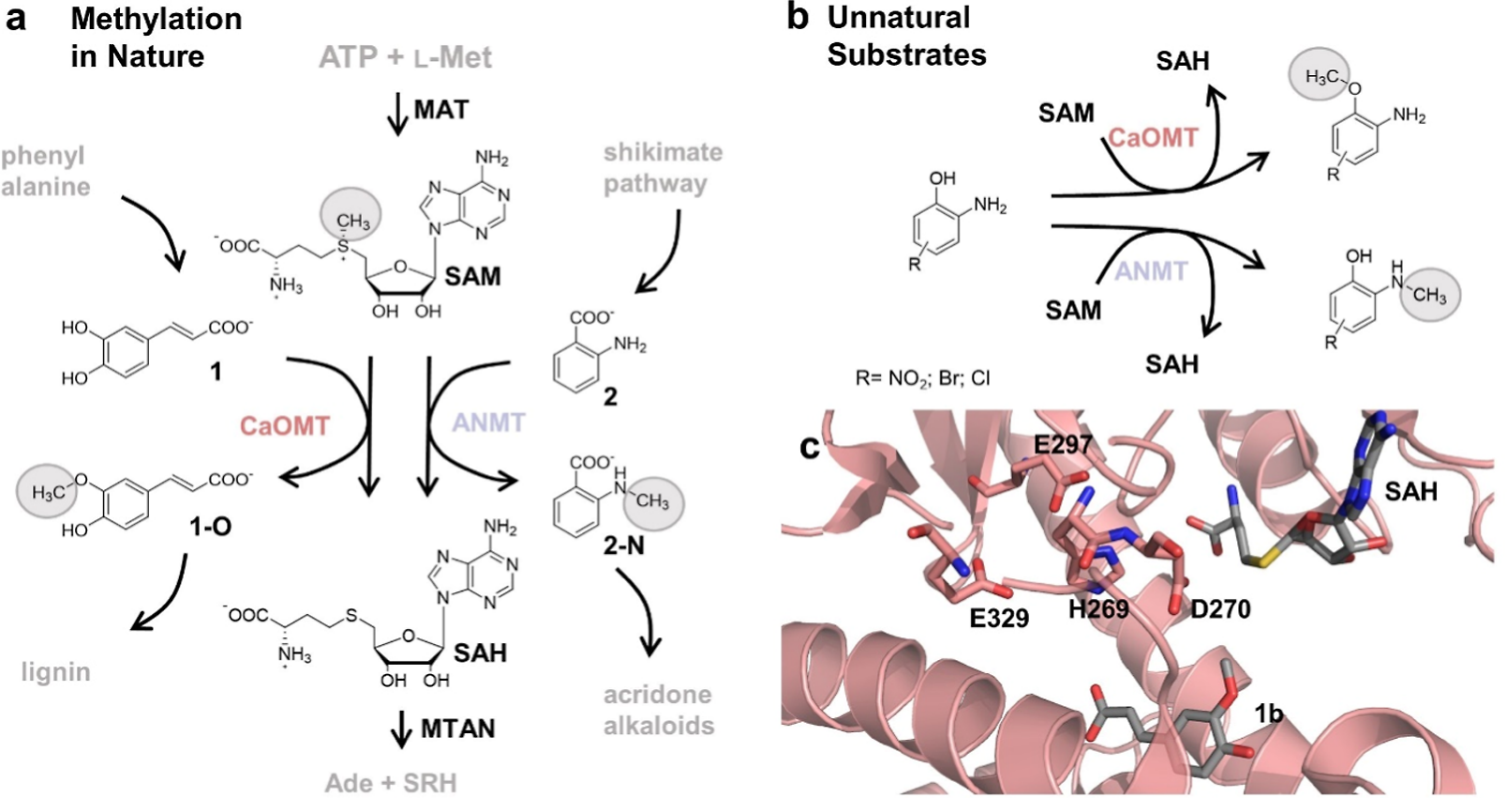
(a) Methylation reactions in nature: The displayed three-enzyme cascade [l-methionine adenosyltransferase (MAT), methyltransferase (MT), methylthioadenosine/-SAH nucleosidase (MTAN)] was used for the methylation reactions in this work. SAM is generated in situ, using ATP and l-methionine (l-Met) as the starting material. The methyl group of SAM is transferred onto different substrates by MTs. In the reaction catalyzed by caffeate *O*-MT (CaOMT), caffeate (**1**) is *O*-methylated in 3-position forming ferulate (**1-O**), a precursor for lignin in plants. Anthranilate *N*-MT (ANMT) transfers the methyl group onto the amino group of anthranilate (**2**) forming *N*-methylanthranilate (**2-N**). **2-N** is further used in acridone alkaloid biosynthesis. After methyl group transfer, SAH is cleaved by MTAN forming adenine (Ade) and *S*-ribosyl-l-homocysteine (SRH). (b) Besides the physiological substrates **1** and **2** (and analogues), also non-physiological substrates [amino phenols with different electron-withdrawing groups (nitro, chloro, and bromo substituents)] are accepted by several CaOMTs and ANMTs. The substrates are strictly chemoselectively converted to the *O*- and *N*-methylated products, respectively. (c) Active site of *Ms*CaOMT cocrystallized with SAH and **2** (PDB code 1KYZ).

The late-stage insertion of methyl groups into pharmaceutically relevant compounds is of great interest due to its effects on chemical and physical properties but requires strict chemoselectivity as many functional groups can be methylated.^7,8^ The potency, half-life, and/or solubility of drug molecules can be changed in different ways.^9^ This phenomenon, described as the *magic methyl effect*, is one reason for the fact that over two-thirds of pharmaceuticals carry at least one of those small carbon fragments.^7,10^*S*-Adenosyl-l-methionine (SAM) is the most common methyl group donor in biology.^11^ The CH_3_ group is enzymatically transferred to nucleophilic substrates by SAM-dependent methyltransferases (MTs; EC 2.1.1.x) forming the methylated substrate and the byproduct *S*-adenosyl-l-homocysteine (SAH).^12^ MTs can be subdivided into *O*-, *N*-, *S*-, *C*-, and halide MTs depending on the (hetero-) atom that is methylated.^13,14^*O*-MTs, which methylate oxygens, make up the largest group. One representative is the caffeate *O*-MT from *Prunus persica* (*Pp*CaOMT; EC 2.1.1.68).^15^ During lignin precursor formation, CaOMTs selectively methylate caffeate (**1**) and analogues thereof in the 3- and 5-position.^16−18^ In previous studies, we investigated the reaction catalyzed by *Pp*CaOMT and compared it with an anthranilate *N*-MT from *Ruta graveolens* (*Rg*ANMT; EC 2.1.1.111).^15^ ANMTs are involved in the biosynthesis of acridone alkaloids in plants and *N*-methylate anthranilate (**2**).^19^ In addition to their physiological substrates, the substrate scope of both enzymes extends to various amino phenols.^15,20^ Although the two enzymes (*Pp*CaOMT; *Rg*ANMT) share high similarity at their amino acid sequence level (>74%), all amino phenols were selectively methylated in either the *O*- or *N*-position (Figure 1a).^15^ Recently, we have shown that *Pp*CaOMT and two catechol *O*-MTs (COMT, EC 2.1.1.6) are capable of *S*-methylation and accept thiols such as thiosalicylate as a substrate.^14^ Compared to hydroxyl groups, thiols are more acidic facilitating the formation of the conjugate base (thiolate) which is a strong nucleophile.^21,22^ In the physiological CaOMT or COMT reaction, the reacting hydroxyl group of the substrate requires to be deprotonated by the enzyme in order to increase the nucleophilicity of the oxygen atom.^16,23,24^ The crystal structure of CaOMT from *Medicago sativa* (*Ms*), cocrystallized with the physiological product ferulic acid (**1-O**) and SAH, was published in 2002 by Zubieta et al. (Figure 1c).^16^ His269 was identified as the catalytic base that deprotonates one hydroxyl group of the catechol in **1** to increase the nucleophilic character for the S_*N*_2-like reaction. Glu329 as a second residue stabilizes His269 by hydrogen bonding, leading to an increased basicity of the histidine. Two adjacent negatively charged residues (Asp270 and Glu297) are thought to be important for electrostatics and correct orientation of the catalytic histidine.^16^ In 2010, Louie et al. published another CaOMT structure from the ryegrass *Lolium perenne* (*Lp*). They described the occurrence of an open and a closed conformation for this enzyme (family) and inferred the importance of conformational dynamics for catalysis. From the crystallographic data, it was concluded that the closed conformation is required for an efficient attack of the SAM methyl group by the catecholic substrate.^25^

According to amino acid alignments, ANMTs feature a histidine residue at the same position as that of CaOMTs. However, an *O*-methylation has not yet been observed for this enzyme family. Furthermore, the neutral amino nitrogen atom is sufficiently nucleophilic to attack the SAM methyl group directly. 2-Amino-4-nitrophenol (**3**) and 2-amino-5-nitrophenol (**4**), as well as substrates with chloro- or bromo substituents replacing the nitro group, were previously tested with the enzymes. The selectively *N*- and *O*-methylated products were formed exclusively by the *Rg*ANMT and *Pp*CaOMT enzymes, respectively (Figure 1b).^15^

The aim of this work was to decipher the factors responsible for the discrimination between *O*- and *N*-methylation catalyzed by the highly related enzymes *Pp*CaOMT and *Rg*ANMT. Based on amino acid alignments, we rationally designed and generated seven variants for each enzyme. Substrates **3** and **4**, which contain both amino and phenol groups as potential methyl acceptors, were used as the main model substrates. The mutations had different effects on catalysis and chemoselectivity, ranging from a (total) loss of activity to the formation of product mixtures (with *O*- and *N*-methylated products) to completely exchanged chemoselectivity. Based on the experimental results [enzyme assays and hydrogen/deuterium exchange coupled with mass spectrometry (HDX-MS) analysis] and computational methods, we pinpoint the molecular basis of the chemoselectivity of both enzyme families and discuss the broader applicability of the knowledge gained.

## Results and Discussion

### Protonation State of the Phenolic Group Enables *O*-Methylation

**Figure 2:**
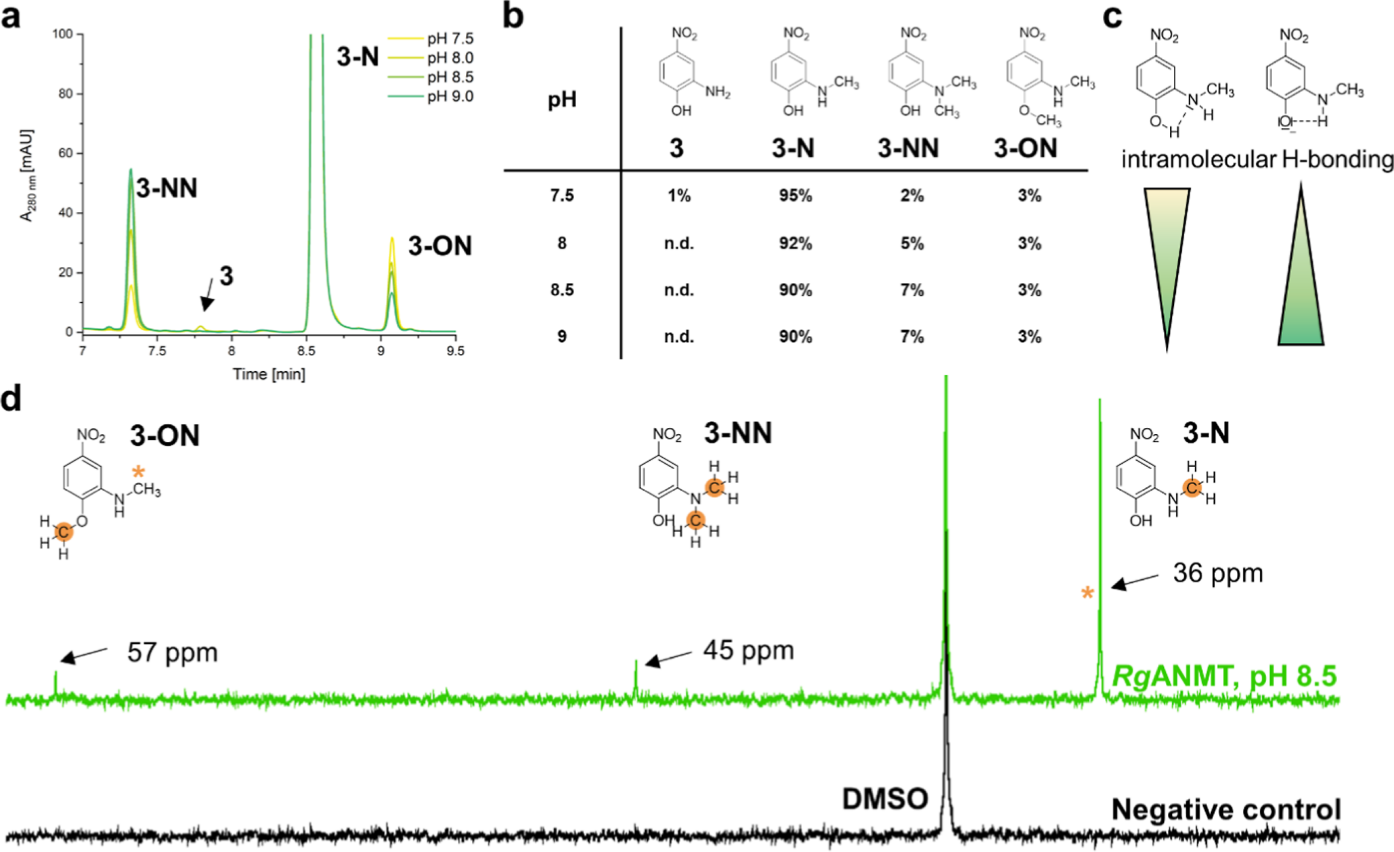
(a) Representative HPLC chromatogram of the reaction catalyzed by ANMT using substrate **3** at pH 7.5; 8.0; 8.5, and 9.0. (b) Distribution of substrate **3** and the formed products after 20 h incubation with ANMT (n.d.—not detected). (c) Intramolecular H-bonding between a phenol proton and adjacent amino group nitrogen/amino group proton and phenolate of **3**. (d) ^13^C NMR of *Rg*ANMT reaction with substrate **3** at pH 8.5 in comparison to a negative control. There are three signals visible that belong to carbons of transferred methyl groups (highlighted in orange). The signal for the labeled (*) carbon atom attached to the amino group in substrate **3-ON** overlaps with the signal from substrate **3-N** at 36 ppm (highlighted with an orange asterisk *).

All enzyme assays were performed in a three-enzyme cascade^26^ as shown in Figure 1a. Substrates **3** and **4** contain both an amino and a phenol group, and the protonation equilibrium of the substrates depends on the pH value of the system (Table 1). The amino group is predicted to be protonated under acidic conditions [pH < 4 (for **3**) and < 4.5 (for **4**)]. The phenol is present in both the neutral and deprotonated form at physiological pH with the neutral form being the dominant species. A higher pH (> 8.5) is required to have substantial concentrations of aminophenolate species for substrate **4** than for substrate **3**. When the two groups are compared, the phenolate exhibits a higher nucleophilicity than the neutral NH_2_ group. Considering this, we tested two buffer systems with different pH values (KPi: pH 5.5; 6.5; 7.0; 7.5; 8.0 and Tris: pH 7.5; 8.0; 8.5; 9.0) to determine whether a shift in chemoselectivity in the *Rg*ANMT-catalyzed reaction can be induced by shifting the acid–base equilibrium of **3** and **4** in solution (Figures 2, S3–S8, S20 and S21, Table S4).

**Table 1:**
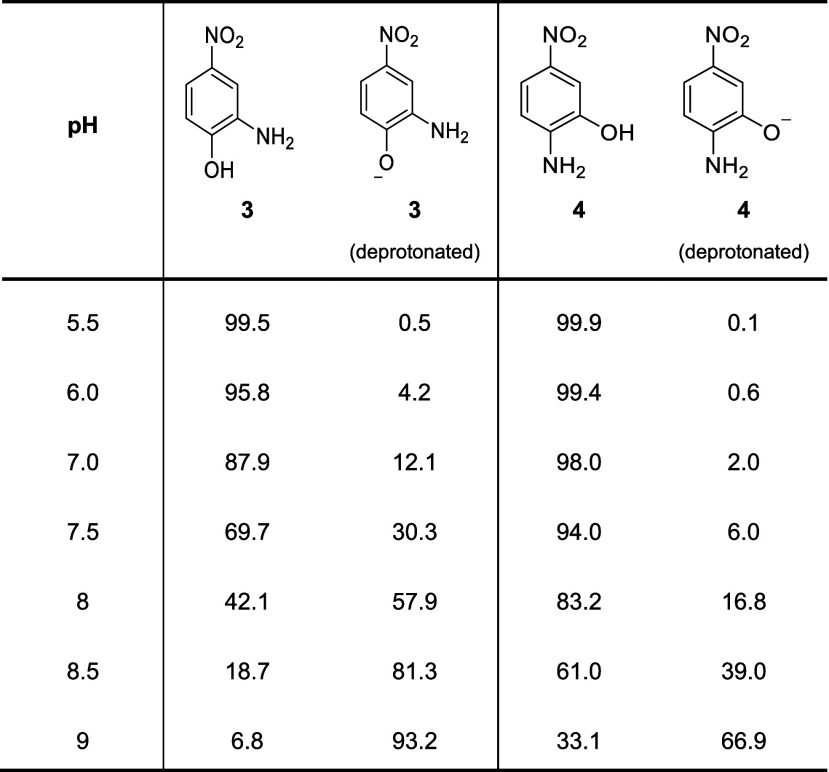
Predicted Acid–Base Equilibrium (in [%] of Species Distribution) for **3** and **4** at Different pH Values^a^

a Protonation state calculations were carried out with Chemicalize based on partial charge distribution calculations for the atoms in the molecule (https://chemicalize.com/, developed by ChemAxon [06, 2024]).

*Rg*ANMT was enzymatically not active at pH 5.5. For all other reactions, the main product was assigned as the *N*-methylated product **3-N**/**4-N** (Figure 2). There were two new products formed at an increased pH value (Figures S3 and S5). One of them was assigned to the previously identified double methylated product carrying the methyl group in the *N*- and *O*-position (**3-ON**/**4-ON**).^15^

LC–MS analysis of the other product formed by *Rg*ANMT (using **3** or **4**) at higher pH values revealed a mass of 183.07 *m*/*z* in positive mode, indicating a transfer of two methyl groups to the corresponding substrates (Figure S8). In the ^13^C NMR spectra of the reaction with **3** or **4**, in addition to a prominent signal at 36 ppm assigned to the carbon of the first methyl group, a second, smaller signal appeared at 45 ppm, indicating a second methylation of the amino nitrogen (Figures 2d, S20, and S21). Interestingly, the amount of the double *N*-methylated product **3-NN** was increased up to 7% at pH 8.5 and 9.0; in contrast, **3-ON** was present in small but constant amount (<5%). Similar patterns were observed for substrate **4** (Figure S5). Stabilization by intramolecular hydrogen bonds between the amino and phenol groups of the substrates might explain the higher amount of the double *N*-methylated products **3-NN** and **4-NN** compared to **3-ON/4-ON** at higher pH values (8.5/9.0). The phenol can form a hydrogen bond to the free electron pair of the nitrogen of the amino group, preventing a second methylation step at the nitrogen, provided the substrate p*K*_a_’s are similar for the enzyme-bound form (Figure 2, lower tested pH values). At higher pH values, the aminophenolate of **3** (or **4**) is present in larger quantities. Intramolecular H-bonding between the amine proton and the phenolate may increase the nucleophilic character of the monomethylated amino group and thus promote a second methylation step, while the nucleophilicity of the phenolate decreases. Notwithstanding, the alkaline conditions might also affect some of the ionizable side chains in the active site of *Rg*ANMT, leading to altered electrostatics and/or structural changes that could reorient the substrate.

As expected, the chemoselectivity of the *Pp*CaOMT-catalyzed reaction was not affected by external changes in buffer pH (Figures S4, S6, and S7). Higher pH values in the *O*-MT reaction merely led to increased amounts of the preferred nucleophile (aminophenolate) and the corresponding methylated product. Changes in buffer pH had no major effect on the chemoselectivity of the *Rg*ANMT- and *Pp*CaOMT-catalyzed reactions: no single *O*- or *N*-methylation (the reverse products), respectively, were detected. Nevertheless, the formation of **3-ON** and **4-ON** was observed in the *Rg*ANMT reaction, which might be related to the formation of intramolecular H-bonds and an increased nucleophilic character of the phenol. In summary, the two enzymes must use individually tailored mechanisms to achieve chemoselectivity control.

### Rationally Designed Enzyme Variants Show Altered Chemoselectivity

**Figure 3:**
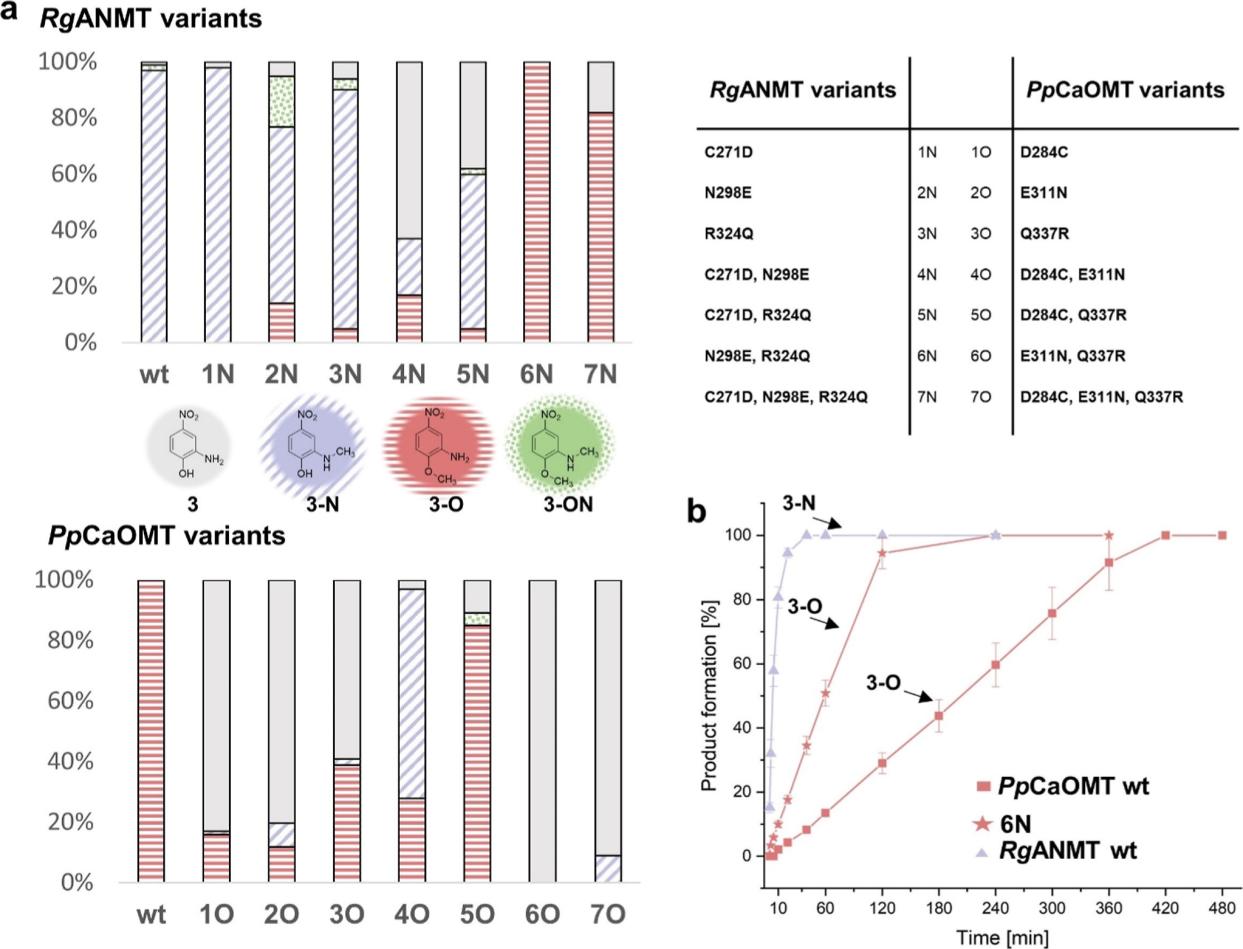
(a) Distribution of substrate **3** and products after 20 h of reaction catalyzed by *Rg*ANMT, *Pp*CaOMT, and corresponding variants. (b) Timeline of reaction with substrate **3** using *Rg*ANMT, *Pp*CaOMT, or variant 6N (N298E, R324Q). *O*-Methylation is shortened by half using 6N (N298E, R324Q) compared to the reaction catalyzed by the *Pp*CaOMT wildtype (wt) enzyme.^15^

With the aim to determine the molecular basis for the different chemoselectivities, in particular the ability of ANMTs to accept amino groups, we re-examined the amino acid sequences of *Rg*ANMT and *Pp*CaOMT and compared them with *Ms*CaOMT as well as *ANMT* and *CaOMT* from *Citrus sinensis* (*Cs*) (multiple sequence alignment in Figure S2). While the putative catalytic base (His269 in *Ms*CaOMT) is present in all enzyme sequences, we found differences in the adjacent residues that have been proposed to be important for the orientation of the catalytic His and the optimal conditions in the active site.^16^ All CaOMTs have polar ionic side-chain residues (for *Ms*CaOMT: Asp284; Glu311), while ANMTs feature neutral residues in the equivalent positions (for *Rg*ANMT: Cys271; Asn298). Based on these findings, two single variants and one double variant with reciprocal exchanges of these residues for *Rg*ANMT and for *Pp*CaOMT were produced by site-directed mutagenesis (Figure 3a). In MD simulation studies with the wildtype *N*-MT and the produced variants with a bound substrate, we observed persistent interactions between the electron-withdrawing group of the substrate (carboxylate of **2**; nitro group of **3**) and the Arg324 residue (Figure S25). *Cs*ANMT also contains an arginine, whereas *Pp*CaOMT and *Cs*CaOMT have glutamine at this position. However, *Ms*CaOMT, as other *O*-MTs, has an alkaline residue (*Ms*CaOMT: His323) at this position, similar to the ANMTs (Figure S2). As the arginine and glutamine side chains are somewhat similar sterically but show different acid–base character, we decided to generate additional variants by switching these two residues (Figure 3a). The variants were produced using a QuikChange PCR protocol, and all proteins were produced in soluble form (for variant designation, see Figure 3a). Chemoselectivity assays were performed at pH 7.5 in Tris buffer (Figures 3 and S9–S19). All but one (6O: E311N, Q337R) variants were enzymatically active. Substantially reduced conversion was observed for some variants (Figure 3a).

*Rg*ANMT variant 1N (C271D) showed no activity toward the phenol and still methylated at high levels at the nitrogen. The exchanged cysteine was already slightly acidic with a calculated p*K*_a_ value of 8.14 for the side chain.^27^ The introduction of only aspartate did not appear to have a major impact, either on the activity or on the chemoselectivity of the enzyme. When a glutamate was introduced instead of the asparagine [2N (N298E)], a small amount of *O*-methylation was observed. In addition to the formation of the main product **3-N** (62%), products **3-O** (14%) and **3-ON** (18%) were detected. The variant 3N (R324Q) gave similar results to 2N. A combination of the mutations N298E and R324Q (6N variant) gave the most exciting results with full conversion and a complete switch from *N*- to *O*-methylation. The triple variant 7N was also exclusively methylating **3** in the *O*-position, nevertheless, with reduced activity (82% conversion) (Figure S9).

For the corresponding *Pp*CaOMT variants, the three candidates with single-point mutations (1O, 2O, and 3O) showed a substantial loss of activity: 59–83% of the substrate was not methylated under the standardized conditions. Nevertheless, *O*- and *N*-methylated products were found after 20 h. The double variant 4O (D284C and E311N) clearly preferred *N*- over *O*-methylation. As described above, *Rg*ANMT variant 6N (N298E, R324Q) delivered the best results regarding a full chemoselectivity switch. The corresponding *O*-MT variant 6O (E311N and Q337R), however, was enzymatically inactive. With an additional residue exchange, triple variant 7O led to conversions of around 10%, exclusively forming *N*-methylated product **3-N** (Figures 3 and S10), exhibiting a complete chemoselectivity switch, albeit with reduced activity.

The same trend was observed using bromo-substituted aminophenols with various substitution patterns as acceptor substrates (substrates **6** to **9**, Figures S16–S19, S23, and S24): both wildtype enzymes exclusively methylated the *N*- or *O*-position (*Rg*ANMT or *Pp*CaOMT, respectively). Further, for most variants tested (2N, 6N, 2O, 4O, and 7O), either a full or a partial swap of chemoselectivity was observed.

In addition, the generated variants were tested with the physiological substrates **1** (caffeate) and **2** (anthranilate) (Figures S11–S15 and S22). Reactions with the ANMT’s physiological substrate **2** revealed similar results as with **3**. With an increased ability of *O*-methylation for the ANMT variants, the acceptance toward **2** was decreased. In the reactions catalyzed by the *Pp*CaOMT variants 4O (D284C and E311N), 5O (D284C and Q337R), and 7O (D284C, E311N, and Q337R), **2-N** was formed in high amounts. A similar behavior was observed for the reactions with **1** (caffeate, physiological substrate CaOMT) or **3**, using the *O*-MT variants. While 1O (D284C), 2O (E311N), and 4O (D284C and E311N) *O*- and *N*-methylate **3**, **1** is still accepted. Triple variant 7O, which only performs *N*-methylation with **3**, does not accept **1** as the substrate. The *N*-MT variants were not active with **1**, which might be due to steric hindrance by the larger side chain of **1**. Moreover, 3,4-dihydroxy benzoic acid (**5**) was tested as a potential physiological compound to accept the methyl group in the *O*-position. Structurally, this substrate resembles amino phenols, which are accepted by ANMTs. None of the *N*-MT variants were able to *O*-methylate **5** (Figure S13). The catechol structure and higher p*K*_a_ values of the catechol (two units higher than those in amino nitrophenols) are possible reasons for substrate rejection.

Previously, we investigated time courses of the *Rg*ANMT and *Pp*CaOMT wildtype enzymes. *Rg*ANMT was much faster than *Pp*CaOMT, completing the *N*-methylation of **3** (0.5 mM) after a 40 min reaction time. *Pp*CaOMT reached full conversion (for *O*-methylation) only after 420 min under identical conditions.^15^ We repeated the experiments with the active enzyme variants and compared the reaction times with the wildtype enzymes. The reaction rate was, in general, reduced compared to the corresponding wildtype enzymes. Variant 1N (C271D) which still *N*-methylates had only formed 30% of **3-N** after 40 min, the point of time when the *Rg*ANMT wildtype reaches full conversion. However, variant 6N (N298E and R324Q) was able to complete *O*-methylation of **3** after 240 min, thereby reducing the reaction time required for the *Pp*CaOMT wildtype enzyme (Figure 3b); this might be an interesting option for the application of these types of enzymes as biocatalysts in chemical synthesis.

### Computational Modeling Reveals Distinct Open/Closed Conformations

In order to further investigate the origins of the different reactivity and chemoselectivity observed for *Rg*ANMT and *Pp*CaOMT, and to gain more insights in the role of the key active site residues identified in the mutational studies, we performed computational modeling based on MD simulations (see the Computational Methods section).

**Figure 4:**
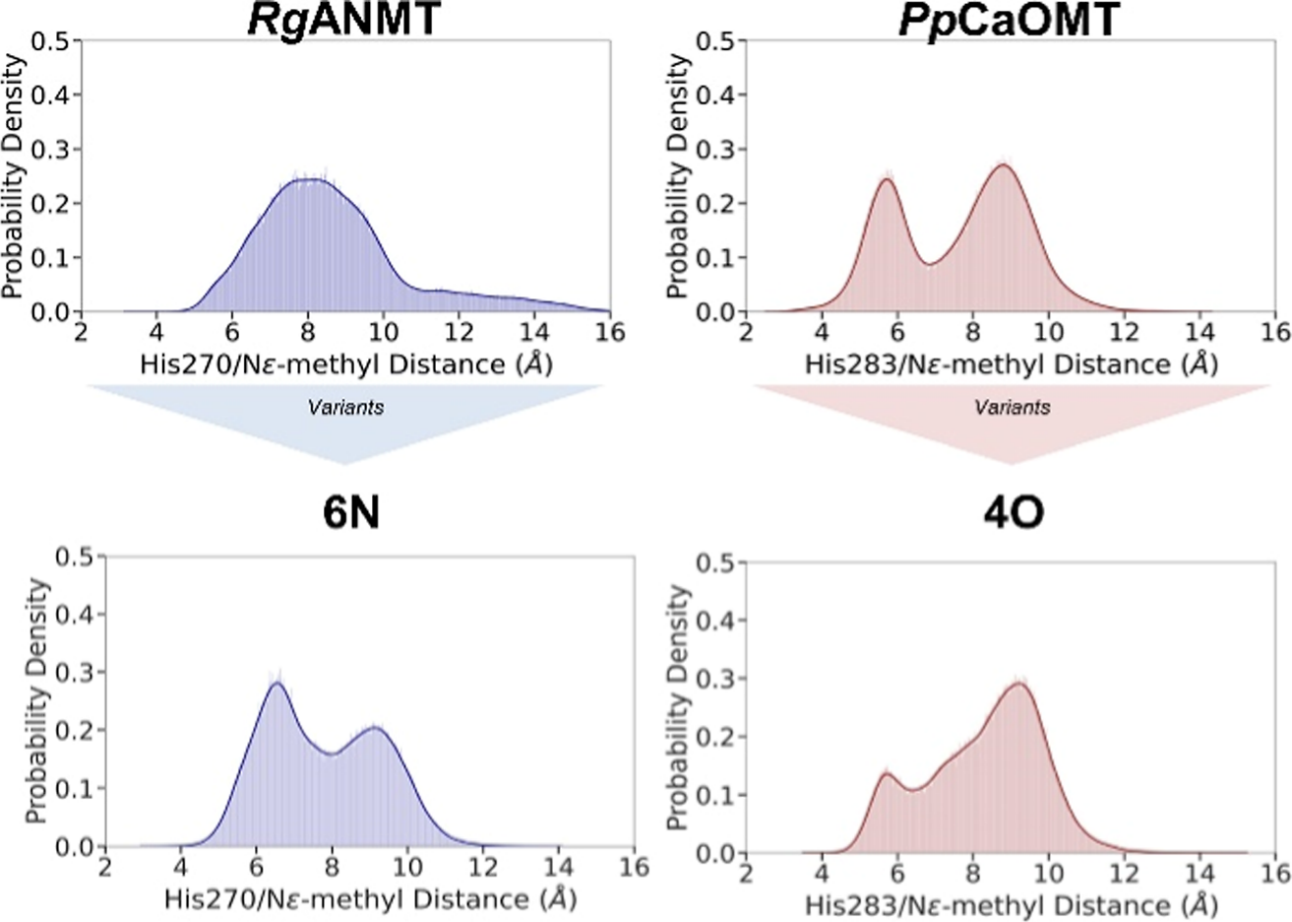
KDE plots of the distance between the catalytic histidine and reactive SAM methyl [His-methyl(SAM)] along the MD trajectories of the wildtype *Rg*ANMT and *Pp*CaOMT enzymes and their corresponding variants **6N** (N298E and R324Q) and **4O** (D284C and E311N). The His–methyl(SAM) distance has been measured between the histidine’s epsilon nitrogen (His270 in *Rg*ANMT, His283 in *Pp*CaOMT) and SAM’s reactive carbon atom. The distance between His270/283 and the methyl group of SAM [methyl(SAM)] describes the open/closed nature of the catalytic pocket (see Figure 5).

**Figure 5:**
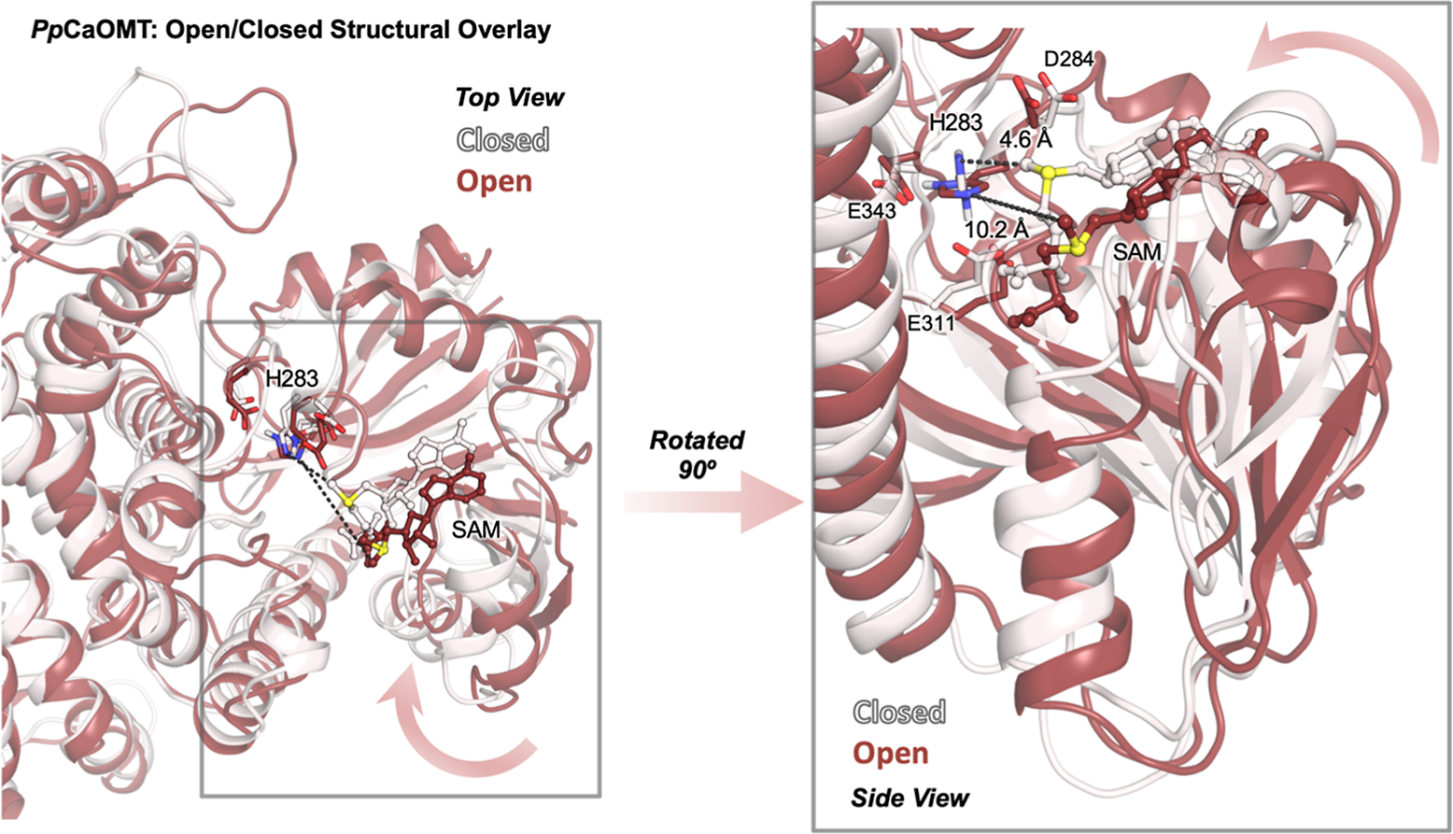
Structural overlay of two representative frames from the MD trajectories of the *Pp*CaOMT holoenzyme model, representing an open (dark-red protein, SAM in dark-red with a sulfur atom in yellow) and a closed conformation (white protein, SAM in white with a sulfur atom in yellow) from top and side views. The arrows indicate the direction of the open to closed transition. Active-site residues His283, Asp284, Glu311, and Glu343 are shown with sticks. The distance between the histidine’s epsilon nitrogen and SAM’s reactive carbon atoms is also reported for each conformation.

MD simulations in the holo state (with bound SAM cofactor) of *Rg*ANMT and *Pp*CaOMT highlighted considerable differences in the flexibility and size of their active site cavities. A similar phenomenon had been observed for crystal structures of *Lp*CaOMT by the group of Noel (pdb: 3p9c).^25^ In order to characterize the open/closed conformational equilibrium of the active site cavity, we monitored the distance between the catalytic histidine (His270/His283) and the SAM methyl group along the MD trajectories for each of the two enzyme monomers (Figures S26–S34). His270/283 is positioned on the opposite structural face of the active site cavity relative to the SAM-binding pocket. The kernel density estimate (KDE) plot obtained from analyzing the simulation data for *Pp*CaOMT disclosed two distinct peaks (Figure 4). This indicates the coexistence of two different conformational states, each linked to a specific set of His283–methyl(SAM) distances: a “closed conformation”, with histidine–methyl(SAM) distances below 6 Å; and an “open conformation”, with histidine–methyl(SAM) distances of over 8 Å (Figure 5). The KDE plot obtained from MD simulations of *Rg*ANMT depicted a single peak, which is centered around the histidine–methyl(SAM) distance that corresponds to the open conformation (Figure 4).

The MD-derived data complements very nicely the X-ray crystallography data from the Noel group, which showed a markedly closed conformation of the product-bound ternary complex, as compared to the structures of the apo and holoenzyme (Figure S35).^25^ These findings highlight the importance of enzyme dynamics in representative CaOMTs. The X-ray structures of *Lp*CaOMT indicate that the apo enzyme (without a cofactor and methyl group acceptor) mainly explores an open-state conformation to facilitate SAM binding, followed by substrate binding, which completes the transition from the open-to-closed state. Based on these observations, we performed restrained MD simulations with the model substrate **3** bound for both *Rg*ANMT and *Pp*CaOMT (details in the Computational Methods section). The MD simulations confirmed a shift toward closed states for *Pp*CaOMT in the presence of the substrate, whereas the ensemble of *Rg*ANMT did not exhibit significant changes with respect to the holo state (Figures S36–48).

Taken together, our MD analysis and published X-ray analysis suggest that in CaOMTs, the closed conformation provides a confined space that facilitates the appropriate substrate positioning in the active site cavity to promote an efficient phenol deprotonation by the catalytic His, followed by a fast *O*-methylation step (Figure S48). The substrate confinement in the closed-conformation active site can prevent protonation of the aminophenolate intermediate by the bulk solvent once formed, thus allowing the *O*-methylation to efficiently take place.

Results obtained from MD simulations further suggest that *Rg*ANMT does not require the same degree of confinement. The *N*-methylation does not require a deprotonation step (as for the *O*-methylation) prior to attack on the SAM methyl carbon. The only requirement for *N*-methylation seems to be productive substrate positioning relative to that of the SAM cofactor.

### Open-to-Closed Conformational Transitions by ActiveSite Mutations

We conducted the same computational analysis with variants 4N (C271D and N298E), 4O (D284C and E311N), and 6N (N298E and R324Q). Compared to their wildtype counterparts, variant 4N (C271D and N298E) explored mostly His–methyl(SAM) distances representative of the closed conformation (Figure S27), whereas the 4O (D284C and E311N) KDE distribution shifted toward an open conformation (Figure 4). The two peaks observed in the KDE plot of 6N (N298E and R324Q) disclosed an open/closed dual character, with the closed state being slightly more populated than the open state (Figure 4).

Swapping the active site residues between *Rg*ANMT and *Pp*CaOMT led to inverted effects in the conformational dynamics of the variants’ active site along MD simulations. In particular, the introduction of negatively charged residues in *Rg*ANMT variants 4N (C271D and N298E) and 6N (N298E and R324Q) stabilizes the closed conformation. These variants show diminished (4N) and abolished (6N) *N*-methylation activity and are capable of *O*-methylation (Figure 3). This effect is further amplified in the presence of the substrate for the 6N variant, where the closed conformation becomes even more stabilized as shown by substrate-bound restrained MD simulations (Figure S39). Conversely, *Pp*CaOMT 4O (D284C and E311N), that is found to prefer *N*-methylation over *O*-methylation (Figure 3), now favorably explores open-state conformations. These observations suggest that *O*-methylation requires access to the closed conformation, whereas *N*-methylation is possible in open-state conformations.

We experimentally probed this assertion by subjecting *Rg*ANMT and *Pp*CaOMT to HDX-MS. We selected the *Rg*ANMT wildtype and the *Rg*ANMT 6N variant due to their clear-cut selectivity for *N*- and *O*-methylation, respectively. For *Pp*CaOMT, we investigated the wildtype protein alongside the 4O variant that, although not completely inverted in selectivity, exhibits a strong preference toward *N*-methylation (approximately 70%, Figure 3). Both wildtype/variant pairs were incubated in deuterium oxide and the degree of hydrogen/deuterium exchange (HDX) quantified by MS whereby differences in HDX between wildtype and variant would be indicative for a discrepancy in their higher-order structure and dynamics.^28^

**Figure 6:**
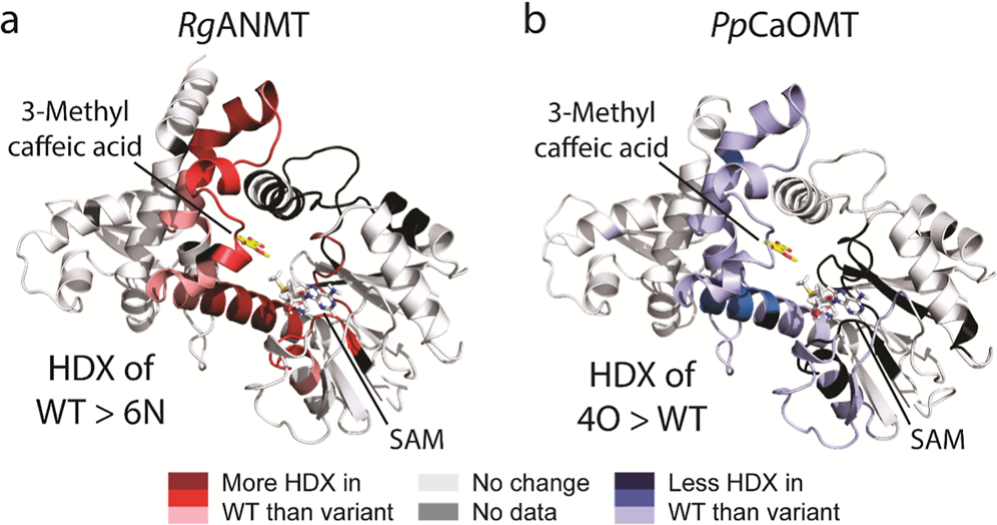
Conformational changes of ANMT and OMT upon active site mutations. The difference in hydrogen/deuterium exchange (HDX) of the *Rg*ANMT wildtype compared to its 6N variant (a) and *Pp*CaOMT wildtype compared to its 4O variant (b) plotted onto structural models of both proteins generated as described in the Computational Methods section, which were used as starting points for MD simulations. Red and blue color denotes higher and lower HDX, respectively, of the WT compared to the variant proteins. The position of the SAM substrate is derived from structural models generated as described in the Computational Methods section; the position of the *O*-MT reaction product ferulic acid (**1-O**) is based on superposition with the structure of *Ms*CaOMT (PDB: 1KYZ).

The differences in HDX between WT and variant for both MTs occur in similar regions lining the methyl acceptor substrate’s binding site (Figure 6). For *Rg*ANMT though, HDX is higher in the strictly *N*-methylating wildtype protein, while the *O*-methylating wildtype *Pp*CaOMT exhibits lower HDX than its 4O variant. Furthermore, the amplitude in observed HDX change is higher for *Rg*ANMT (>20%, Figure S53) than for *Pp*CaOMT (10–15%, Figure S54), agreeing with the latter’s wildtype/4O variant pair not being entirely inverted in selectivity. Collectively, these data experimentally support the theory inferred by MD and crystallographic data that *N*-methylation and *O*-methylation selectivity are linked to the open and closed conformational states of *Rg*ANMT and *Pp*CaOMT, respectively.

### Electrostatic Interactions Drive Protein Conformational Dynamics

**Figure 7:**
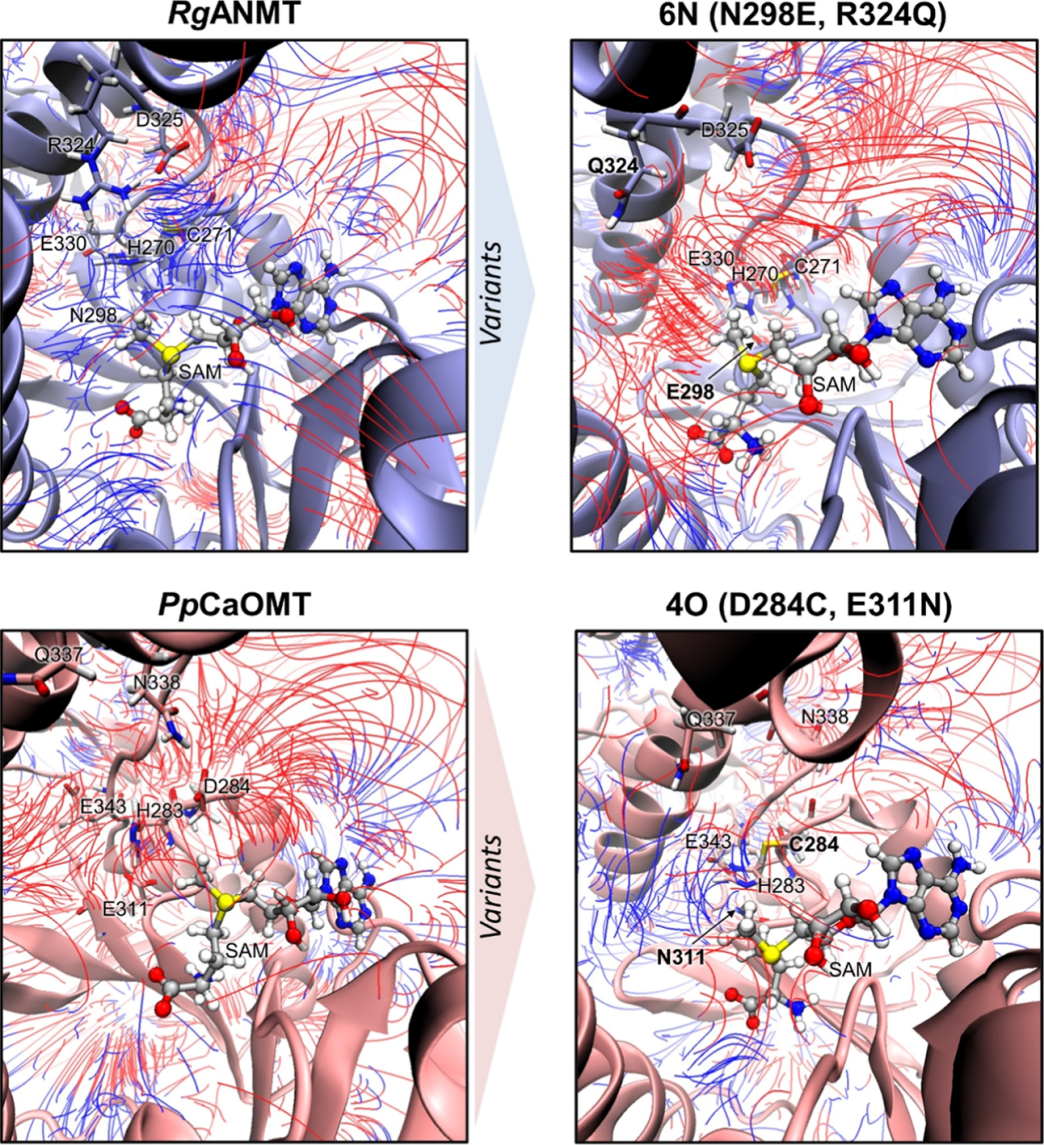
Map of the electric field in the active site of *Rg*ANMT and *Pp*CaOMT and their corresponding variants **6N** (N298E and R324Q) and **4O** (D284C and E311N). The electrostatic properties are depicted with color-coded electric field lines: the red-colored line segments indicate negative electrostatic potentials, while blue segments represent positive electrostatic potentials. The direction of the field lines indicates the direction of attractive electrostatic interactions. Only electric field lines with a gradient magnitude above 2.23 *kT*/eÅ are shown. Activesite residues are represented with sticks; activesite mutations have been marked in variants 6N and 4O. See the Computational Methods sectionfor details on selection of MD snapshots used for the analysis.

Principal component analysis (PCA)^29^ was used to retrieve the most representative conformations from MD trajectories of each studied system [*Rg*ANMT, *Pp*CaOMT, 4N (C271D and N298E), 4O (D284C and E311N), and 6N (N298E and R324Q)]. Selecting representative structures for each of these conformations, the electrostatic potential and electric field map (electrostatic potential gradient) over the three-dimensional space was analyzed using the Adaptive Poisson–Boltzmann Solver (APBS) (Figures 7 and S49–S52).^30^

Electrostatic analysis of the individual active sites revealed a neutral to positively charged gradient region (from the catalytic His region to the SAM-binding region) within the active site of *Rg*ANMT and *Pp*CaOMT variant 4O (D284C and E311N). The catalytic cavity of *Pp*CaOMT, and *Rg*ANMT variants 4N (C271D and N298E) and 6N (N298E and R324Q), is characterized by dominant negative electrostatic potentials around the catalytic His residue (His283 in *Pp*CaOMT, His270 in *Rg*ANMT) and an increased density of positive-to-negative field lines (from the catalytic His region to SAM-binding region). In *Pp*CaOMT, the electric field gradient lines connect the negatively charged catalytic histidine area with the positively charged SAM cofactor-binding region (Figure 7). A similar scenario is found for variants 6N (N298E and R324Q) and 4N (C271D and N298E) (Figures 7 and S51), in which the electrostatic interactions between these two regions of the catalytic pocket are also observed. These two variants can efficiently explore closed conformations along the MD trajectories (Figure 4).

Nevertheless, these strong electrostatic interactions are not observed in the wildtype *Rg*ANMT enzyme, which is found to be mainly in an open conformation during the whole MD simulations, and in the promiscuous 4O (D284C and E311N) variant, which barely explores closed conformations along the MD trajectories (Figure 4). Therefore, these results indicate that closed conformational states for these enzymes are highly stabilized by the electrostatic interactions arising from residues of the activesite cavity. Similar strategies for characterizing electrostatic interactions driving open-to-closed transitions or conformational rearrangements required for catalysis have been employed in the study of other enzymatic systems, such in CRISPR-Cas9^31^ or in self-sufficient P450s.^32^

## Conclusions

A wide range of methods can be combined to introduce a desired selectivity or functionality in enzymes: e.g., directed evolution, (semi)rational design, molecular, and dynamic modeling. There are many examples of successful engineering of enzymes in terms of stereoselectivity, broadening or changing the substrate range or switching to another activity.^33−36^ A complete switch of the (chemoselectivity) of an enzyme by engineering is rarely achieved. Compared to, e.g., the reversal of stereoselectivity, which “only” requires a different binding mode, a switch in chemoselectivity might also require different substrate activation, in addition to changes in substrate and cofactor binding. Recently, Shende et al. described a change from C–C to C–N bond formation by affecting the conformational flexibility of a diketopiperazine dimerase. Their study was based on differences in selectivity of two enzyme homologues.^37^ Our current study focuses on two related enzymes that perform a methyl transfer to different nucleophiles: amino and phenol groups. In this case, *O*-methylation was found to require an extensive conformational change of the enzyme’s active site mediated by interactions of active site residues to align the phenol of the substrate near the catalytic histidine for facilitated deprotonation, followed by a fast attack on the SAM methyl group, as previously described.^25^ MD simulations as well as HDX-MS measurements allowed us to observe this conformational change in the *O*-MT *Pp*CaOMT. Simulations with the *N*-MT *Rg*ANMT enzyme described a preference for an open conformational state. It is proposed that this difference in conformational dynamics has an impact on the chemoselective preferences of these two enzyme families: closed conformations facilitate the positioning of the appropriate substrate in the active site to allow an efficient deprotonation of the phenol group by the catalytic His, followed by a rapid *O*-methylation, while reprotonation of the aminophenolate intermediate by the bulk solvent is prevented; this is not required for *N*-methylation.

Computational modeling suggests that the conformational dynamics of these enzymes are influenced by different electrostatic environments of the active site: *Pp*CaOMT has predominantly negative electrostatic potentials in the region where the catalytic His is located. Together with the positively charged cofactor, this leads to a stabilization of the closed conformation. Although it appears that these electrostatic interactions in the variants can be altered by an exchange of several key residues, they are also influenced by other parts of the enzyme, and the change of the conformational dynamics must be considered as an interplay of several factors. These findings support that biocatalytic reactions are not only determined by a few numbers of residues but also by the interplay and movement of the enzyme as a whole.

In addition, the product range (e.g., toward *N*,*N*-dimethylation) could be controlled by external factors such as pH, which in turn controls the protonation state of the substrate. Further adjustment of the reaction conditions might be a way to increase the conversion to a specific product. A recent example from psilocybin biosynthesis shows another solution for *N*,*N*-dimethylation: a single-point mutation was sufficient to abolish or allow dimethylation due to steric constraints.^38^

Taking together, controlling the (chemo)selectivity of an enzyme requires a network of components including enzyme, substrate, cofactor, and reaction conditions, which often act synergistically. An important question is how these different enzymatic activities with dissimilar chemoselectivities evolve.^39−41^ It is assumed that enzymes from natural product biosynthesis have a special role, as they have to evolve comparably fast. Also, these enzymes might tend to remain in a generalist stage where both the original and the new activities are high.^39^ For the ANMT/CaOMT example presented here, this does not seem to be the case, as all enzymes we have analyzed so far evolved as either *O*- or *N*-MTs. Some of the variants [e.g., 1N (*O*- and *N*-methylating **3**) and 2O (methylating **1**, **2**, and *O*- and *N*-methylating **3**)] tested in this study could be considered generalists, as they perform *O*- and *N*-methylation. As lignin biosynthetic pathways and thus CaOMTs occur in many plants, we assume, that in this case, ANMTs have evolved from CaOMTs and have not remained at the generalist level but have further specialized into pure *N*-MTs by losing the ability to adopt the closed enzyme conformation.

## Methods

### Materials

All substrates were purchased from Sigma-Aldrich (ATP, SAM, SAH, l-methionine, l-methionine-(methyl-^13^C), 2-amino-4-nitro phenol, 2-amino-5-nitro phenol, 2-methoxy-4-nitro aniline, and 2-methoxy-5-nitro aniline) in the highest purity available (see also Table S1). Buffer ingredients and cultivation media were obtained from Carl Roth.

### Molecular Biology/Protein Biochemistry Methods

#### Mutagenesis Experiments

The *Ec*MAT, *Rg*ANMT, *Pp*CaOMT, and *Ec*MTAN plasmids have been described in previous publications.^15,26,42^ The primers used for the mutagenesis experiments were purchased at Eurofins (Sequences in Table S2). For the PCR, 20 ng of the DNA template (gene or vector) was used. Additionally, the samples contained 2.5 μL of the forward and reverse primer (10 μmol L^–1^), 25 μL 2X Phusion Flash PCR Master Mix (Thermo Fisher), and were filled up to 50 μL with H_2_O_Millipore_. A 3-step PCR protocol was performed (Table 2). After amplification, DpnI digestion (according to the manufacturer’s instruction; New England Biolabs) was performed. PCR samples were analyzed by agarose gel electrophoresis (1% agarose, 100 V, 60 min). Second and third mutations were inserted analogously.

**Table 2:** 3-Step PCR Protocol

step	temperature [°C]	time [s]	cycles
initial denaturation	98	30	1
denaturation	98	10	30
annealing	55	30	
elongation	72	90	
final elongation	72	240	1
storage	8	∞	

#### Protein Overproduction

*E. coli* BL21-Gold(DE3) cells were transformed with the plasmid carrying the desired gene. One colony was added to 5 mL LB medium containing kanamycin (50 μg L^–1^) and incubated at 37 °C, 170 rpm overnight. The preculture (1%) was added to 400 mL LB medium containing kanamycin (50 μg L^–1^) and incubated at 37 °C, 170 rpm until the OD600 was between 0.5 and 0.7. Isopropyl-β-d-thiogalactopyranoside (IPTG) was added (final concentration 0.25 mM) to induce the overexpression. The overproduction of the enzymes took place at 20 °C and 140 rpm for 20 h. Cells were harvested by centrifugation (4 °C, 8000 rpm, 7.8 × 1000*g*, 20 min) and stored at 4 °C until following purification.

#### Protein Purification

The harvested pellets were resuspended (4 mL g^–1^) in a lysis buffer [40 mM Tris–HCl, pH 8.0, 100 mM, NaCl, 10% (w/v) glycerol]. Cell lysis was performed using a sonifier (Branson Sonifier 250, Emerson, St. Louis, MO, USA [duty cycle 50%, intensity 50%, 5 × 30 s with 0.5 min breaks within]). The lysis solution was centrifuged at 4 °C for 40 min (24.9 × 1000*g*). The crude lysate was then applied to a nickel-NTA column followed by washing and elution steps. 30 mL lysis buffer containing 10 mM imidazole was used for washing, followed by the elution step using 20 mL lysis buffer with 250 mM imidazole. Afterward, the protein solution was desalted using PD-10 columns (GE Healthcare Life Sciences, Little Chalfont, UK) according to the manufacturer’s instructions. The concentration of the protein solution was determined with a NanoDrop 2000 instrument (Thermo Fisher Scientific, Waltham, MA, USA) at 280 nm. The molecular weight and extinction coefficient (including His_6_-tag) were calculated with the ExPASy ProtParam tool (https://web.expasy.org/protparam/) for further concentration measurements via NanoDrop.

### Assays

Each experiment was performed in at least triplicates. A standard assay to investigate the chemoselectivity of the produced MT (variants) was prepared in 200 μL with 50 mM Tris at pH 7.5, 20 mM MgCl_2_, and 50 mM KCl. 10 μM MAT and MT and 2 μM MTAN enzymes were used. 3 mM ATP and l-methionine and 2 mM substrate were added. Assays were incubated at 37 °C, 300 rpm and samples were taken after 1 and 20 h. The reaction was stopped by the addition of perchloric acid (total conc. 2.5%) and the samples were stored at −4 °C until analysis. For samples analyzed by ^13^C NMR, ^13^C-labeled l-methionine was used as a substrate. For the pH screening experiment, the pH of the Tris buffer was adjusted (pH: 7.5; 8; 8.5; and 9) or replaced by a KPi buffer system with different pH values (5.5; 6.0; 7.0; 7.5; and 8.0).

For the time course assay, the enzyme and substrate concentrations were adapted. The concentrations of the MAT and MT enzymes were 3 μM and of MTAN 1 μM. ATP and l-methionine concentrations were 1 mM and the MT substrate concentration was 0.5 mM.

Conversion rates were calculated from the substrate and product AUC at certain time points.
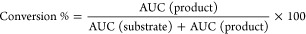


For significance tests, a two-sided *t*-test of two independent samples of the same variance was used (**3-NN**/**3-ON** or **4-NN**/**4-ON**). Significance was confirmed when *p* < 0.05.

### Analytical Methods

#### HPLC Analysis

The HPLC method used for the chemoselectivity studies, as well as the analysis of the time-course experiments, has been described in detail in a previous study (HPLC method B for MT substrates and products).^43^

#### LC–MS Analysis

An LC–MS method that has been described earlier to analyze MT substrates and products (LC–MS method B) was used to analyze the unknown peak found in the pH screening experiment of the wildtype MT reactions.^43^ The samples were diluted to 1:50 and filtered before measurement. A Q1 scan was performed in positive mode.

#### NMR Analysis

A Bruker AVANCE DRX 400 instrument, operating at 100.6 MHz, was used to analyze the chemoselective substrate methylation by ^13^C NMR analysis as described earlier.^15^ Spectra were recorded at 25 °C and analyzed using TopSpin 3.6.2. Prior to the analysis, 5% D_2_O was added to the enzyme reaction samples. In Table S3, the ^13^C signals for the used assay compounds are listed. The signals for the carbon attached to the amino or hydroxyl group are displayed in the NMR figures (Figures S20–S24).

#### Hydrogen/Deuterium Exchange Mass Spectrometry

HDX-MS experiments were conducted essentially as described previously.^44^ In experiment 1, the HDX behavior of *Pp*CaOMT WT was compared side-by-side with that of its 4O variant, while experiment 2 examined the behavior of *Rg*ANMT WT and its 6N variant. Purified proteins were employed at stock concentrations of 50 μM supplemented with 1 mM SAM (Supporting Information Data Set 1 online). From these sample stocks, HDX reactions were prepared by a two-arm robotic autosampler (LEAP technologies) as follows: 7.5 μL of the sample was predispensed and then 67.5 μL of HDX buffer (20 mM Tris-Cl pH 8.0, 100 mM NaCl, 1 mM SAM) prepared with 99.9% D_2_O was added followed by incubation at 25 °C for 10; 30; 100; 1000; or 10,000 s. Then, 55 μL of the HDX reaction was withdrawn, transferred to 55 μL of predispensed quench buffer (400 mM KH_2_PO_4_/H_3_PO_4_, pH 2.2, 2 M guanidine-HCl), and kept at 1 °C, and 95 μL of the resulting mixture was injected into an ACQUITY UPLC M-Class System with HDX Technology (Waters).^45^ The preparation of nondeuterated samples was carried out similarly (incubation for approximately 10 s at 25 °C) by 10-fold dilution of samples with HDX buffer prepared with H_2_O. Injected samples were flushed out of the 50 μL loop with H_2_O + 0.1% (v/v) formic acid (100 μL/min) and guided to a column (2 mm × 2 cm, 12 °C) containing porcine pepsin immobilized to beads, and the resulting peptic peptides were trapped on an AQUITY UPLC BEH C18 1.7 μm 2.1 × 5 mm VanGuard Precolumn (Waters) kept at 0.5 °C. After 3 min of digestion and peptide trapping, the trap column was placed in line with an ACQUITY UPLC BEH C18 1.7 μm 1.0 × 100 mm column (Waters), and the peptides eluted at 0.5 °C using a gradient of eluents A [H_2_O + 0.1% (v/v) formic acid] and B [acetonitrile + 0.1% (v/v) formic acid] at a flow rate of 30 μL/min as follows: 0–7 min: 95–65% A; 7–8 min: 65–15% A; 8–10 min: 15% A; 10–11 min: 5% A; 11–16 min: 95% A. Eluting peptides were guided to a G2-Si HDMS mass spectrometer with ion mobility separation (Waters) and ionized with an electrospray ionization source (250 °C capillary temperature, 3.0 kV spray voltage) and mass spectra acquired in positive-ion mode over a range of 50 to 2000 *m*/*z* in enhanced high-definition MS (HDMS^E^) or high-definition MS (HDMS) mode for nondeuterated and deuterated samples, respectively.^46,47^ Lock-mass correction was conducted with the [Glu1]-Fibrinopeptide B standard (Waters). During separation of the peptide mixtures on the C18 column, the protease column was washed three times with 80 μL of wash solution (0.5 M guanidine hydrochloride in 4% (v/v) acetonitrile), and blank injections were performed between each sample to reduce peptide carry-over. Measurements were conducted in technical triplicate (individual HDX reactions).

ProteinLynx Global SERVER (PLGS, Waters) and DynamX 3.0 softwares (Waters) facilitated peptide identification and analysis of deuterium incorporation essentially as described previously.^44^ In brief, peptides were identified with PLGS from the nondeuterated samples acquired with HDMS^E^ by employing low energy, elevated energy, and intensity thresholds of 300, 100, and 1000 counts, respectively. Identified ions were matched to peptides with a database containing the amino acid sequence of either *Pp*CaOMT or *Rg*ANMT (both as WT sequences), porcine pepsin, and their reversed sequences with the following search parameters: peptide tolerance = automatic; fragment tolerance = automatic; min fragment ion matches per peptide = 1; min fragment ion matches per protein = 7; min peptide matches per protein = 3; maximum hits to return = 20; maximum protein mass = 250,000; primary digest reagent = nonspecific; missed cleavages = 0; false discovery rate = 100. Only peptides identified in two non-deuterated samples with a minimum intensity of 10,000 counts, a peptide length of 5–40 residues, a minimum number of two products, a maximum mass error of 25 ppm, and retention time tolerance of 0.5 min were considered for further analysis. All spectra were manually inspected with DynamX 3.0 software (Waters) and, if necessary, peptides omitted (e.g., in the case of a low signal-to-noise ratio or presence of overlapping peptides). Whenever possible, multiple charge states were employed for the quantification of deuterium uptake. The observable maximal deuterium uptake of a peptide (Supporting Information Data Set 1 online) was calculated by the number of amino acid residues minus one (for the N-terminal residue) minus the number of proline residues. For the calculation of HDX in percent, the absolute HDX was divided by the theoretical maximal deuterium uptake multiplied by 100. To distinguish the residue-specific HDX from overlapping peptides, the shortest peptide covering a residue was employed. Where multiple peptides were of the shortest length, the peptide with the residue closest to the peptide’s C-terminus was utilized.

### Computational Methods

#### Protein Structure Prediction with Homology Modeling

Since the structures of *Rg*ANMT and *Pp*CaOMT are not available from the Protein Data Bank, we resorted to homology modeling to predict their structures. Utilizing the SWISS-MODEL web server (https://swissmodel.expasy.org/),^48−52^ we built 3D models for each enzyme by inputting their FASTA sequences and selecting *M. sativa* CaOMT (PDB ID: 1KYZ) as a template.^16^

Rohde et al. reported the evolutionary connection between *Rg*ANMT and the CaOMT family and suggested *Ms*CaOMT (CaOMT prototype) as a suitable template for homology or comparative modeling.^19^ In particular, PDB entry 1KYZ provides the crystallographic structure of *Ms*CaOMT in complex with SAH and the natural product ferulate, ensuring a reliable representation of the cofactor fitting into the enzyme.

#### Assignment of Protonation States

PROPKA 3^53,54^ and H++ (version 4.0; http://biophysics.cs.vt.edu/H++)^55−57^ methods were employed to predict the protonation states of *Rg*ANMT and *Pp*CaOMT residues at pH 7.5 (normal pH conditions). PROPKA 3 was applied through the PDB2PQR web server (https://server.poissonboltzmann.org/pdb2pqr),^58−60^ maintaining all default options. The default parameters were also kept in the H++ web server, with the exception of the internal dielectric constant, which was adjusted from 10 to 6.

For each enzyme, we compared the p*K*_a_ estimations of the titratable residues between the two chains and between the two computational methods. Visual inspection of the local environment around dubious titratable residues helped decide on the most appropriate protonation form; residues localized close to the SAM cofactor were also manually checked. The predictions obtained from H++ for chain A were assumed and translated to chain B, hence maintaining the same protonation states between the two chains of the homodimer. The protonation states of some histidine residues were set to the neutral tautomeric form with Nδ protonated (HID); those are *Rg*ANMT’s His195 and His358 and *Pp*CaOMT’s His173, His195, His238, His252, and His283.

Finally, the N-terminal was neutralized with the “ACE” capping group and the C-terminal with the “NME” capping group.

#### Ligand Assembly into the Protein Model

The SAM cofactor was manually docked into the *Rg*ANMT- and *Pp*CaOMT-protonated homology models. *Rg*ANMT and *Pp*CaOMT models were aligned with the 1KYZ*Ms*CaOMT complex using PyMOL’s^61^ pair fitting tool, and the SAM cofactors in the two chains of the models were placed at the same position of the two SAH cofactors in 1KYZ*Ms*CaOMT. For the simulations of *Rg*ANMT with substrate **3** (2-amino-4-nitrophenol), the substrate was pair fitted at the same position of the two ferulate products from the 1KYZ*Ms*CaOMT complex.

#### Generation of the Variant Models

We mutated the selected positions of the generated wildtype protein models utilizing the mutagenesis tool from the PyMOL software package.^61^ In that way, we build the following models for the variants: 4N (*Rg*ANMT C271D and N298E), 6N (*Rg*ANMT D284C and E311N), and 4O (*Pp*CaOMT N298E and R324Q). PROPKA 3^53,54^ and H++ (version 4.0)^55,57^ methods were employed again to verify if the introduced mutations altered significantly the p*K*_a_ estimations of the residues. We confirmed that the protonation states assigned for the wildtype enzymes were also valid for the variants.

#### Molecular Dynamics Simulations

Amber 2020,^62^ comprised of Amber20 and AmberTools20 software packages, was employed to set up and perform MD simulations in explicit water. We studied the following systems: (1) the holoenzyme forms of wildtype *Rg*ANMT and *Pp*CaOMT and variants 4N, 6N, and 4O; (2) the substrate-bound ternary complex of *Rg*ANMT and 4N with substrate **3**; (3) the substrate-bound ternary complex of *Rg*ANMT, *Pp*CaOMT, and 6N with substrate **3** restrained to the reactive methyl of SAM; and (4) the substrate-bound ternary complex of *Rg*ANMT, *Pp*CaOMT, and 6N with substrate **3** restrained to the catalytic histidine. We ran 3 independent replicas of 500 ns per system, accumulating a total simulation time of 1.5 μs for each system.

Specific restraints were applied in certain substrate-bound MD simulations (see the technical details below) to prevent unbinding events along MD trajectories and enhance the sampling of substrate-bound conformations relevant to our studies.

General processing and analysis of the MD trajectories were carried out with the cpptraj^63^ module from AmberTools, and the visual assessment of the simulations was conducted with the VMD graphics program.^64^ The root-mean-square deviation (RMSD) of all alpha carbons was monitored relative to the first frame of the production run.

We utilized the LEaP module from AmberTools to prepare every system for MD simulations. The Stony Brook modification of the Amber14 force field (ff14SB)^65^ was used to describe the protein, the general AMBER force field 2 (GAFF2)^66^ to describe the SAM cofactor and substrate **3**, the TIP3P^67^ force field to describe the explicit water molecules, and the Li-Merz ion parameters in TIP3P water^68^ to describe the added counterions. Each enzyme complex was neutralized with the addition of Na^+^/Cl^–^ ions and solvated in a pre-equilibrated cuboid box with a 10 Å buffer of explicit water molecules in the TIP3P model.^68^

To obtain the GAFF2 parameters of the SAM cofactor and substrate **3**, we used the following protocol. First, we employed Gaussian16^69^ to compute the partial atomic charges under the Merz–Singh–Kollman scheme.^70,71^ From the optimized ligand structure, this single-point calculation was conducted at the HF/6-31G(d) level of theory with an implicit CPCM solvent model of diethyl ether.^72,73^ The antechamber module from AmberTools was used to assign the restrained electrostatic potential (RESP) charges for each atom and to generate the GAFF2 parameters.^74^

The energy minimization stage followed a two-step geometry optimization approach. The first minimization imposed restraints on the solute molecules with a harmonic force constant of 500 kcal mol^–1^ Å^–2^, thus optimizing only the positions of counterions and water molecules. The second is an unrestrained minimization of all the atoms in the simulation box. The two calculations involved 2500 cycles in the steepest descent method, followed by 2500 cycles in the conjugate gradient method. The energy minimization stage was performed under constant volume and periodic boundary conditions, applying a 10 Å cutoff to calculate nonbonded interactions and utilizing the particle mesh Ewald (PME) method to model long-range electrostatic interactions.^75−77^

The following heating, equilibration, and production stages also proceeded under periodic boundary conditions, with a 10 Å cutoff to calculate nonbonded interactions and with the PME method to model long-range electrostatics. In addition, the Langevin thermostat was chosen to control the temperature, setting a collision frequency of 1 ps^–1^.^78^ The bond length in bonds involving hydrogen atoms was constrained with the SHAKE algorithm, removing the bond-stretching motion and its rapid time scale. The time step in the molecular dynamics simulation stages was set to 2 fs.^79,80^

The minimized systems were heated progressively using six steps of 20 ps, incrementing the temperature by 50 K with each step. Positional restraints were imposed on the solute molecules, decreasing the magnitude of the harmonic force constant with each step: 210 kcal mol^–1^ Å^–2^ in the 0–50 K step, 165 kcal mol^–1^ Å^–2^ in the 50–100 K step, 125 kcal mol^–1^ Å^–2^ in the 100–150 K step, 85 kcal mol^–1^ Å^–2^ in the 150–200 K step, 45 kcal mol^–1^ Å^–2^ in the 200–250 K step, and 10 kcal mol^–1^ Å^–2^ in the 250–300 K step. The heating stage was performed under constant-volume conditions.

At a constant temperature of 300 K, each system was equilibrated for 2 ns under constant pressure conditions of 1 bar. The pressure was controlled with the Berendsen barostat.^81^ After equilibration in the NPT ensemble, production trajectories of 500 ns were propagated under constant volume and temperature conditions (NVT ensemble).

The two sets of substrate-bound restrained-MD simulations included a distance constraint in the equilibration and production stages to prevent the substrate from leaving the catalytic cavity. In the first, the distance between the center of the substrate’s aromatic ring and the methyl carbon of SAM was restrained with a 50 kcal mol^–1^ Å^–2^ harmonic force constant to keep it below the 6.9 Å. In the second, the distance between the center of the substrate’s aromatic ring and the epsilon nitrogen from the catalytic histidine was similarly restrained with a 50 kcal mol^–1^ Å^–2^ harmonic force constant to keep it below 7.7 Å. These distance thresholds were chosen based on the observation of previous substrate-bound unrestrained-MD trajectories.

#### Principal Component Analysis

We utilized the cpptraj^63^ module from AmberTools to prepare MD data for PCA. The three trajectories of each holoenzyme system were reduced, retaining one frame in every ten frames and removing the solvent (counterions and water molecules). All trajectory frames were aligned to the first frame from the first replica.

Subsequently, we employed the PyEMMA 2.5.7 python package^82^ to perform PCA^83−85^ on the preprocessed MD trajectories, considering only the structural coordinates of the alpha carbons. The k-means algorithm was used,^86,87^ also integrated in PyEMMA 2.5.7, to cluster the frames into 20 cluster centers based on their principal component dimensions.

#### Electrostatic Analysis

By means of PCA and clustering analysis, we identified the most populated and representative conformations of the protein explored during MD trajectories. For each holoenzyme system, a representative trajectory frame was selected from the cluster located in the global minimum of the PC1 and PC2 spaces; if more than one cluster was located in the minimum, the most populated cluster was chosen. The distance between the catalytic histidine and the reactive SAM methyl group along the frames was used for constituting the clusters; the average distance values were retrieved for chains A and B. We selected the representative frame for electrostatic analysis by matching its two histidine–methyl distances with the averages from the cluster.

To perform the electrostatic analysis, we first utilized the ambpdb module from AmberTools to convert the selected trajectory pdb frame into the pqr format, including all protein residues and cofactors while removing water molecules and counter ions. The APBS (version 1.4.2) software^60^ through the VMD graphics program^64^ was used to estimate the electrostatic potential over each representative pqr frame. The APBS electrostatic calculations were performed implementing the linearized Poisson–Boltzmann equation with a grid spacing of approximately 0.5 Å. We used an internal dielectric constant of 2 and set the remaining options to their default values,^60^ including an implicit solvent model with a dielectric constant of 78.54, a temperature of 298.15 K, and a mobile-ion concentration of 0.150 M.

The electrostatic properties are depicted with color-coded electric field lines. The red color indicates negative electrostatic potentials (more negative values than −0.001 *kT*/*e* are shown in red), while the blue represents positive electrostatic potentials (more positive values than +0.001 *kT*/*e* are shown in blue). Values in between −0.001 and 0.001 *kT*/*e* are shown in white.

For visualization purposes and to facilitate the interpretation of the results, in the reported figures, only gradient electric field lines of length longer than 1 Å and gradient magnitude greater than 2.23 *kTe*^–1^ Å^–1^ are represented. This visualization strategy is equivalent to the one used previously by Palermo and co-workers.^31^

## Supporting Information Available

Used substrates and products with abbreviations and retention times; oligonucleotides used for mutagenesis; ^13^C NMR signals for compounds used in the methylation assays; protein sequences; SDS-PAGE analysis; protein sequence alignment; chromatograms of pH , variant, and substrate screening; NMR spectra; data from computational analysesand hydrogen/deuterium exchange – mass spectrometry (PDF)
Summary of the conditions used for the HDX analyses and a full list of the peptides and residue-specific HDX values obtained in the different experiments (XLSX)

## Abbreviations

SAM: *S*-adenosyl-l-methionine
SAH: *S*-adenosyl-l-homocysteine
l-Met: l-methionine
MAT: methionine adenosyltransferase
MTAN: methylthioadenosine/*S*-adenosyl-l-homocysteine nucleosidase
CaOMT: caffeate *O*-methyltransferase
ANMT: anthranilate *N*-methyltransferase
MD: molecular dynamics
KDE: kernel density estimation
PCA: principle component analysis
HDX: hydrogen/deuterium exchange
