# Supplementary material for: How to Tell an N from an O: Controlling the Chemoselectivity of Methyltransferases
Source: ACS Catal. 2025 Apr 4;15(8):6410–25. doi: 10.1021/acscatal.5c00834 (PMC12013660; doi:10.1021/acscatal.5c00834)
Supplement: Supplementary file 1 — cs5c00834_si_001.pdf [file cs5c00834_si_001.pdf]

Supporting Information

How to Tell an N from an O: Controlling the Chemoselectivity of Methyltransferases

Emely Jockmann‡,[a] Helena Girame‡,[b] Wieland Steinchen,[c,d] Kalle Kind,[a] Gert Bange,[c,d] Kai Tittmann,[e] Michael Müller,[a] Ferran Feixas,[b] Marc Garcia-Borràs,[b] Jennifer N. Andexer*[a]*

[a] Institute of Pharmaceutical Sciences, Pharmaceutical and Medicinal Chemistry University of Freiburg, Albertstr. 25, 79104 Freiburg, Germany; E-mail: jennifer.andexer@pharmazie.uni-freiburg.de

[b] Institut de Química Computacional i Catàlisi and Departament de Química, Universitat de Girona, C/ Maria Aurèlia Capmany, 69, 17003 Girona, Spain; E-mail: marc.garcia@udg.edu

[c] Center for Synthetic Microbiology, Philipps University Marburg, Karl-von-Frisch-Str. 14, 35043 Marburg, Germany

[d] Department of Chemistry, Philipps University Marburg, Hans-Meerwein-Str. 4, 35043 Marburg, Germany

[e] Schwann-Schleiden-Forschungszentrum - Department of Molecular Enzymology, Georg-August-Universität Göttingen, Julia-Lermontowa-Weg 3, 37077 Göttingen, Germany

‡ equal contributions

* corresponding authors

Table of Contents

Supplementary Tables 1
Protein sequences 2
Supplementary figures 6
SDS-PAGE analysis 6
Amino acid alignment 7
Experimental data 8
pH screening 8
Variant screening 14
NMR analysis 25
Computational modelling 29
Hydrogen/deuterium exchange – mass spectrometry 55
Supporting References 57

Supplementary Tables

Table S1 – Used substrates and products with abbreviations and retention times.

Substrate	Abbreviation / #	Retention Time [min]
L-methionine	L-Met	-
adenosine triphosphate	ATP	1.1
S-adenosyl-L-methionine	SAM	1.3
S-adenosyl-L-homocysteine	SAH	1.2
adenine	Ade	1.6
2-amino-4-nitrophenol	**3**	7.8
2-(methylamino)-4-nitrophenol	**3-N**	8.6
2-methoxy-5-nitroanilin	**3-O**	8.7
2-methoxy-*N*-methyl-5-nitroaniline	**3-ON**	9.2
2-(dimethylamino)-4-nitrophenol	**3-NN**	7.1
2-amino-5-nitrophenol	**4**	8.2
2-(methylamino)-5-nitrophenol	**4-N**	8.7
2-methoxy-4-nitroanilin	**4-O**	8.8
2-methoxy-*N*-methyl-4-nitroaniline	**4-ON**	9.1
2-(dimethylamino)-5-nitrophenol	**4-NN**	8.4
2-amino-3-bromophenol	**6**	8.4
3-bromo-2-(methylamino)phenol	**6-N**	7.1
2-bromo-6-methoxyaniline	**6-O**	9.1
2-amino-4-bromophenol	**7**	7.7
4-bromo-2-(methylamino)phenol	**7-N**	8.6
5-bromo-2-methoxyaniline	**7-O**	8.9
2-amino-5-bromophenol	**8**	7.2
5-bromo-2-(methylamino)phenol	**8-N**	7.5
4-bromo-2-methoxyaniline	**8-O**	8.4
3-amino-5-bromophenol	**9**	7.8
3-bromo-5-(methylamino)phenol	**9-N**	8.5
3-bromo-5-methoxyaniline	**9-O**	8.9

Table S2 – Primers for mutagenesis.

Primer	Sequence 5´-3´
mut_*rg*anmtC272D_fwd	TCTGCATGATTGGGATGATGAACAGTGTCTGC
mut_*rg*anmtC272D_rv	TCCCAATCATGCAGAATCCATTTCATCAGAATG
mut_*rg*anmtN298E_fwd	TGTTATGGAAAGCGTTGTTCCGGAAACA
mut_*rg*anmtN298E_rv	ACGCTTTCCATAACAATCACTTTGCCATTTTCC
mut_*rg*anmtR324Q_fwd	TGTTCTGCTGATGACCCAGGATGGTG
mut_*rg*anmtR324Q_rv	CACCATCCTGGGTCATCAGCAGAACA
mut_*pp*caomtD284C_fwd	TCTGCATTGTTGGAGCGACGAACATTG
mut_*pp*caomtD284C_rv	CTCCAACAATGCAGAATCCATTTCATAAAAATG
mut_*pp*caomtE311N_fwd	TGTTGTGAATGCACTGCTGCCTGCAATGC
mut_*pp*caomtE311N_rv	AGTGCATTCACAACAATAACTTTGCCGTTATCC
mut_*pp*caomtQ337R_fwd	GATGACCCGTAATCCTGGTGGTAAAGAACGTAGC
mut_*pp*caomtQ337R_rv	GGATTACGGGTCATCATCAGAACATCCAGC

Table S3 – ^{13}C-NMR signals for compounds used in the methylation assays.

	δ [ppm]
Methionine (S-C̲H₃)	13.8
SAM (S-C̲H₃)	23.5
Methionine (H₂N-C̲H)	59.4
Tris (HO-C̲H₂)	61.6
Glycerol (C̲H₂)	62.7
Glycerol (C̲H)	72.1

Protein sequences

The following protein sequences and enzyme sizes include the His$_6$-tag (marked in bold). Mutations / switched residues are highlighted in red.

>*Ec*MAT (44.1 kDa)

MGSS**HHHHHH**SSGLVPRGSHMAKHLFTSESVSEGHPDKIADQISDAVLDAILEQDPKARVA
CETYVKTGMVLVGGEITTSAWVDIEEITRNTVREIGYVHSDMGFDANSCAVLSAIGKQSPDIN
QGVDRADPLEQGAGDQGLMFGYATNETDVLMPAPITYAHRLVQRQAEVRKNGTLPWLRPD
AKSQVTFQYDDGKIVGIDAVVLSTQHSEEIDQKSLQEAVMEEIIKPILPAEWLTSATKFFINPT
GRFVIGGPMGDCGLTGRKIIVDTYGGMARHGGGAFSGKDPSKVDRSAAYAARYVAKNIVAA
GLADRCEIQVSYAIGVAEPTSIMVETFGTEKVPSEQLTLLVREFFDLRPYGLIQMLDLLHPIYK
ETAAYGHFGREHFPWEKTDKAQLLRDAAGLK

>*Ec*MTAN (26.5 kDa)

MGSS**HHHHHH**SSGLVPRGSHMKIGIIGAMEEEVTLLRDKIENRQTISLGGCEIYTGQLNGTE
VALLKSGIGKVAAALGATLLLEHCKPDVIINTGSAGGLAPTLKVGDIVVSDEARYHDADVTAF
GYEYGQLPGCPAGFKADDKLIAAAEACIAELNLNAVRGLIVSGDAFINGSVGLAKIRHNFPQA
IAVEMEATAIAHVCHNFNVPFVVVRAISDVADQQSHLSFDEFLAVAAKQSSLMVESLVQKLA
HG

>*Pp*CaOMT (43.7 kDa)

MGSS**HHHHHH**SSGLVPRGSHMASSLERKSHPKINHAEPEDEITKEEEDESFCYAMQLVGS
SVLSMSLQSAIKLGIFDIIARKGPGAKLSSSEIATKIGTENPEAPVMVDRILRLLTSHSVLNCSA
VAANGGSDFQRVYSLGPVSKYFVNDEEGGSLGPLLTLIQDRVFLESWSQLKDAVVEGGIPF
NRVHGMHAFEYPGLDPRFNQVFNTAMFNHTTIVIKKLLHIYKGLEDKNLTQLVDVGGGLGVT
LNLITSRYQHIKGINFDLPHVVNHAPSYPGVEHVGGDMFASVPSGDAIFMKWILHDWSDEHC
LKLLKNCYKAIPDNGKVIVVEALLPAMPETSTATKTTSQLDVLMMTQNPGGKERSEQEFMAL
ATGAGFSGIRYECFVCNFWVMEFFK

>*Rg*ANMT (42.2 kDa)

MGSS**HHHHHH**SSGLVPRGSHMGSLSESHTQYKHGVEVEEDEEESYSRAMQLSMAIVLPM
ATQSAIQLGVFEIIAKAPGGRLSASEIATILQAQNPKAPVMLDRMLRLLVSHRVLDCSVSGPA
GERLYGLTSVSKYFVPDQDGASLGNFMALPLDKVFMESWMGVKGAVMEGGIPFNRVHGM
HIFEYASSNSKFSDTYHRAMFNHSTIALKRILEHYKGFENVTKLVDVGGGLGVTLSMIASKYP
HIQAINFDLPHVVQDAASYPGVEHVGGNMFESVPEGDAILMKWILHCWDDEQCLRILKNCY
KATPENGKVIVMNSVVPETPEVSSSARETSLLDVLLMTRDGGGRERTQKEFTELAIGAGFKG
INFACCVCNLHIMEFFK

>*Pp*CaOMT D284C (1O)

MGSS**HHHHHH**SSGLVPRGSHMASSLERKSHPKINHAEPEDEITKEEEDESFCYAMQLVGS
SVLSMSLQSAIKLGIFDIIARKGPGAKLSSSEIATKIGTENPEAPVMVDRILRLLTSHSVLNCSA
VAANGGSDFQRVYSLGPVSKYFVNDEEGGSLGPLLTLIQDRVFLESWSQLKDAVVEGGIPF
NRVHGMHAFEYPGLDPRFNQVFNTAMFNHTTIVIKKLLHIYKGLEDKNLTQLVDVGGGLGVT
LNLITSRYQHIKGINFDLPHVVNHAPSYPGVEHVGGDMFASVPSGDAIFMKWILH**C**WSDEHC
LKLLKNCYKAIPDNGKVIVVEALLPAMPETSTATKTTSQLDVLMMTQNPGGKERSEQEFMAL
ATGAGFSGIRYECFVCNFWVMEFFK

>*Pp*CaOMT E311N (2O)

MGSS**HHHHHH**SSGLVPRGSHMASSLERKSHPKINHAEPEDEITKEEEDESFCYAMQLVGS
SVLSMSLQSAIKLGIFDIIARKGPGAKLSSSEIATKIGTENPEAPVMVDRILRLLTSHSVLNCSA
VAANGGSDFQRVYSLGPVSKYFVNDEEGGSLGPLLTLIQDRVFLESWSQLKDAVVEGGIPF
NRVHGMHAFEYPGLDPRFNQVFNTAMFNHTTIVIKKLLHIYKGLEDKNLTQLVDVGGGLGVT
LNLITSRYQHIKGINFDLPHVVNHAPSYPGVEHVGGDMFASVPSGDAIFMKWILHDWSDEHC
LKLLKNCYKAIPDNGKVIVV**N**ALLPAMPETSTATKTTSQLDVLMMTQNPGGKERSEQEFMAL
ATGAGFSGIRYECFVCNFWVMEFFK

>*Pp*CaOMT Q337R (3O)

MGSS**HHHHHH**SSGLVPRGSHMASSLERKSHPKINHAEPEDEITKEEEDESFCYAMQLVGS
SVLSMSLQSAIKLGIFDIIARKGPGAKLSSSEIATKIGTENPEAPVMVDRILRLLTSHSVLNCSA
VAANGGSDFQRVYSLGPVSKYFVNDEEGGSLGPLLTLIQDRVFLESWSQLKDAVVEGGIPF
NRVHGMHAFEYPGLDPRFNQVFNTAMFNHTTIVIKKLLHIYKGLEDKNLTQLVDVGGGLGVT
LNLITSRYQHIKGINFDLPHVVNHAPSYPGVEHVGGDMFASVPSGDAIFMKWILHDWSDEHC
LKLLKNCYKAIPDNGKVIVVEALLPAMPETSTATKTTSQLDVLMMT**R**NPGGKERSEQEFMAL
ATGAGFSGIRYECFVCNFWVMEFFK

>*Pp*CaOMT D284C, E311N (4O)

MGSS**HHHHHH**SSGLVPRGSHMASSLERKSHPKINHAEPEDEITKEEEDESFCYAMQLVGS
SVLSMSLQSAIKLGIFDIIARKGPGAKLSSSEIATKIGTENPEAPVMVDRILRLLTSHSVLNCSA
VAANGGSDFQRVYSLGPVSKYFVNDEEGGSLGPLLTLIQDRVFLESWSQLKDAVVEGGIPF
NRVHGMHAFEYPGLDPRFNQVFNTAMFNHTTIVIKKLLHIYKGLEDKNLTQLVDVGGGLGVT
LNLITSRYQHIKGINFDLPHVVNHAPSYPGVEHVGGDMFASVPSGDAIFMKWILH**C**WSDEHC
LKLLKNCYKAIPDNGKVIVV**N**ALLPAMPETSTATKTTSQLDVLMMTQNPGGKERSEQEFMAL
ATGAGFSGIRYECFVCNFWVMEFFK

>*Pp*CaOMT D284C, Q337R (5O)

MGSS**HHHHHH**SSGLVPRGSHMASSLERKSHPKINHAEPEDEITKEEEDESFCYAMQLVGS
SVLSMSLQSAIKLGIFDIIARKGPGAKLSSSEIATKIGTENPEAPVMVDRILRLLTSHSVLNCSA
VAANGGSDFQRVYSLGPVSKYFVNDEEGGSLGPLLTLIQDRVFLESWSQLKDAVVEGGIPF
NRVHGMHAFEYPGLDPRFNQVFNTAMFNHTTIVIKKLLHIYKGLEDKNLTQLVDVGGGLGVT
LNLITSRYQHIKGINFDLPHVVNHAPSYPGVEHVGGDMFASVPSGDAIFMKWILH**C**WSDEHC
LKLLKNCYKAIPDNGKVIVVEALLPAMPETSTATKTTSQLDVLMMT**R**NPGGKERSEQEFMAL
ATGAGFSGIRYECFVCNFWVMEFFK

>*Pp*CaOMT E311N, Q337R (6O)

MGSS**HHHHHH**SSGLVPRGSHMASSLERKSHPKINHAEPEDEITKEEEDESFCYAMQLVGS
SVLSMSLQSAIKLGIFDIIARKGPGAKLSSSEIATKIGTENPEAPVMVDRILRLLTSHSVLNCSA
VAANGGSDFQRVYSLGPVSKYFVNDEEGGSLGPLLTLIQDRVFLESWSQLKDAVVEGGIPF
NRVHGMHAFEYPGLDPRFNQVFNTAMFNHTTIVIKKLLHIYKGLEDKNLTQLVDVGGGLGVT
LNLITSRYQHIKGINFDLPHVVNHAPSYPGVEHVGGDMFASVPSGDAIFMKWILHDWSDEHC
LKLLKNCYKAIPDNGKVIVV**N**ALLPAMPETSTATKTTSQLDVLMMT**R**NPGGKERSEQEFMAL
ATGAGFSGIRYECFVCNFWVMEFFK

>*Pp*CaOMT D284C, E311N, Q337R (7O)

MGSS**HHHHHH**SSGLVPRGSHMASSLERKSHPKINHAEPEDEITKEEEDESFCYAMQLVGS
SVLSMSLQSAIKLGIFDIIARKGPGAKLSSSEIATKIGTENPEAPVMVDRILRLLTSHSVLNCSA
VAANGGSDFQRVYSLGPVSKYFVNDEEGGSLGPLLTLIQDRVFLESWSQLKDAVVEGGIPF
NRVHGMHAFEYPGLDPRFNQVFNTAMFNHTTIVIKKLLHIYKGLEDKNLTQLVDVGGGLGVT
LNLITSRYQHIKGINFDLPHVVNHAPSYPGVEHVGGDMFASVPSGDAIFMKWILH**C**WSDEHC
LKLLKNCYKAIPDNGKVIVV**N**ALLPAMPETSTATKTTSQLDVLMMT**R**NPGGKERSEQEFMAL
ATGAGFSGIRYECFVCNFWVMEFFK

>*Rg*ANMT C271D (1N)

MGSS**HHHHHH**SSGLVPRGSHMGSLSESHTQYKHGVEVEEDEEESYSRAMQLSMAIVLPM
ATQSAIQLGVFEIIAKAPGGRLSASEIATILQAQNPKAPVMLDRMLRLLVSHRVLDCSVSGPA
GERLYGLTSVSKYFVPDQDGASLGNFMALPLDKVFMESWMGVKGAVMEGGIPFNRVHGM
HIFEYASSNSKFSDTYHRAMFNHSTIALKRILEHYKGFENVTKLVDVGGGLGVTLSMIASKYP
HIQAINFDLPHVVQDAASYPGVEHVGGNMFESVPEGDAILMKWILH**D**WDDEQCLRILKNCY
KATPENGKVIVMNSVVPETPEVSSSARETSLLDVLLMTRDGGGRERTQKEFTELAIGAGFKG
INFACCVCNLHIMEFFK

>*Rg*ANMT N298E (2N)

MGSS**HHHHHH**SSGLVPRGSHMGSLSESHTQYKHGVEVEEDEEESYSRAMQLSMAIVLPM
ATQSAIQLGVFEIIAKAPGGRLSASEIATILQAQNPKAPVMLDRMLRLLVSHRVLDCSVSGPA
GERLYGLTSVSKYFVPDQDGASLGNFMALPLDKVFMESWMGVKGAVMEGGIPFNRVHGM
HIFEYASSNSKFSDTYHRAMFNHSTIALKRILEHYKGFENVTKLVDVGGGLGVTLSMIASKYP
HIQAINFDLPHVVQDAASYPGVEHVGGNMFESVPEGDAILMKWILHCWDDEQCLRILKNCY
KATPENGKVIVM**E**SVVPETPEVSSSARETSLLDVLLMTRDGGGRERTQKEFTELAIGAGFKG
INFACCVCNLHIMEFFK

>*Rg*ANMT R324Q (3N)

MGSS**HHHHHH**SSGLVPRGSHMGSLSESHTQYKHGVEVEEDEEESYSRAMQLSMAIVLPM
ATQSAIQLGVFEIIAKAPGGRLSASEIATILQAQNPKAPVMLDRMLRLLVSHRVLDCSVSGPA
GERLYGLTSVSKYFVPDQDGASLGNFMALPLDKVFMESWMGVKGAVMEGGIPFNRVHGM
HIFEYASSNSKFSDTYHRAMFNHSTIALKRILEHYKGFENVTKLVDVGGGLGVTLSMIASKYP
HIQAINFDLPHVVQDAASYPGVEHVGGNMFESVPEGDAILMKWILHCWDDEQCLRILKNCY
KATPENGKVIVMNSVVPETPEVSSSARETSLLDVLLMT**Q**DGGGRERTQKEFTELAIGAGFK
GINFACCVCNLHIMEFFK

>*Rg*ANMT C271D, N298E (4N)

MGSS**HHHHHH**SSGLVPRGSHMGSLSESHTQYKHGVEVEEDEEESYSRAMQLSMAIVLPM
ATQSAIQLGVFEIIAKAPGGRLSASEIATILQAQNPKAPVMLDRMLRLLVSHRVLDCSVSGPA
GERLYGLTSVSKYFVPDQDGASLGNFMALPLDKVFMESWMGVKGAVMEGGIPFNRVHGM
HIFEYASSNSKFSDTYHRAMFNHSTIALKRILEHYKGFENVTKLVDVGGGLGVTLSMIASKYP
HIQAINFDLPHVVQDAASYPGVEHVGGNMFESVPEGDAILMKWILH**D**WDDEQCLRILKNCY
KATPENGKVIVM**E**SVVPETPEVSSSARETSLLDVLLMTRDGGGRERTQKEFTELAIGAGFKG
INFACCVCNLHIMEFFK

>*Rg*ANMT C271D, R324Q (5N)

MGSS**HHHHHH**SSGLVPRGSHMGSLSESHTQYKHGVEVEEDEEESYSRAMQLSMAIVLPM
ATQSAIQLGVFEIIAKAPGGRLSASEIATILQAQNPKAPVMLDRMLRLLVSHRVLDCSVSGPA
GERLYGLTSVSKYFVPDQDGASLGNFMALPLDKVFMESWMGVKGAVMEGGIPFNRVHGM
HIFEYASSNSKFSDTYHRAMFNHSTIALKRILEHYKGFENVTKLVDVGGGLGVTLSMIASKYP
HIQAINFDLPHVVQDAASYPGVEHVGGNMFESVPEGDAILMKWILH**D**WDDEQCLRILKNCY
KATPENGKVIVMNSVVPETPEVSSSARETSLLDVLLMT**Q**DGGGRERTQKEFTELAIGAGFK
GINFACCVCNLHIMEFFK

>*Rg*ANMT N298E, R324Q (6N)

MGSS**HHHHHH**SSGLVPRGSHMGSLSESHTQYKHGVEVEEDEEESYSRAMQLSMAIVLPM
ATQSAIQLGVFEIIAKAPGGRLSASEIATILQAQNPKAPVMLDRMLRLLVSHRVLDCSVSGPA
GERLYGLTSVSKYFVPDQDGASLGNFMALPLDKVFMESWMGVKGAVMEGGIPFNRVHGM
HIFEYASSNSKFSDTYHRAMFNHSTIALKRILEHYKGFENVTKLVDVGGGLGVTLSMIASKYP
HIQAINFDLPHVVQDAASYPGVEHVGGNMFESVPEGDAILMKWILHCWDDEQCLRILKNCY
KATPENGKVIVM**E**SVVPETPEVSSSARETSLLDVLLMT**Q**DGGGRERTQKEFTELAIGAGFKG
INFACCVCNLHIMEFFK

>*Rg*ANMT C271D, N298E, R324Q (7N)

MGSS**HHHHHH**SSGLVPRGSHMGSLSESHTQYKHGVEVEEDEEESYSRAMQLSMAIVLPM
ATQSAIQLGVFEIIAKAPGGRLSASEIATILQAQNPKAPVMLDRMLRLLVSHRVLDCSVSGPA
GERLYGLTSVSKYFVPDQDGASLGNFMALPLDKVFMESWMGVKGAVMEGGIPFNRVHGM
HIFEYASSNSKFSDTYHRAMFNHSTIALKRILEHYKGFENVTKLVDVGGGLGVTLSMIASKYP
HIQAINFDLPHVVQDAASYPGVEHVGGNMFESVPEGDAILMKWILH**D**WDDEQCLRILKNCY
KATPENGKVIVM**E**SVVPETPEVSSSARETSLLDVLLMT**Q**DGGGRERTQKEFTELAIGAGFKG
INFACCVCNLHIMEFFK

Supplementary figures

SDS-PAGE analysis

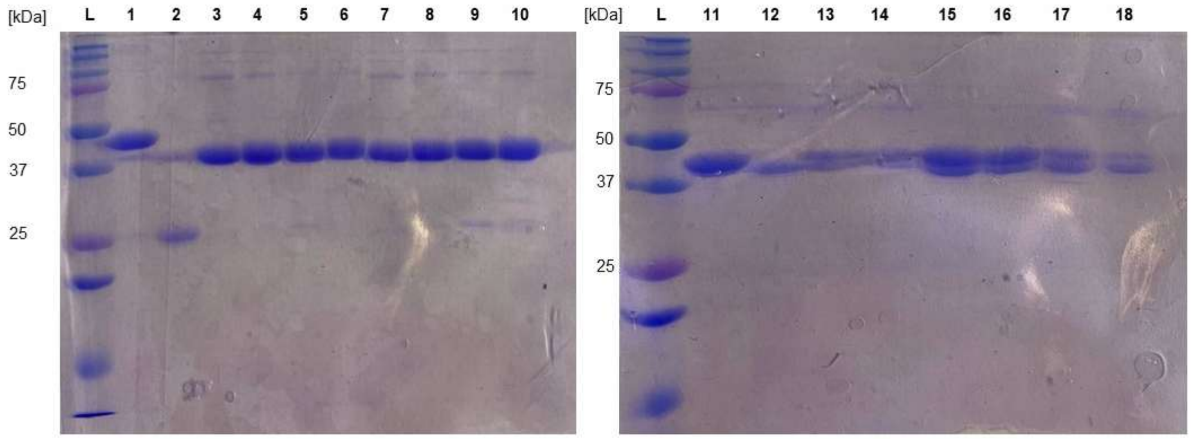

Figure S1 – Visualisation of the enzymes used in this study via SDS-PAGE analysis. L=Ladder. 1-18: Desalted enzymes used in assays. 1: *Ec*MAT (44.1 kDa); 2: *Ec*MTAN (26.5 kDa); 3: *Rg*ANMT (42.2 kDa); 4: *Rg*ANMT C271D; 5: *Rg*ANMT N298E; 6: *Rg*ANMT C271D N298E; 7: *Rg*ANMT R324Q; 8: *Rg*ANMT C271D R324Q; 9: *Rg*ANMT N298E R324Q; 10: *Rg*ANMT C271D N298E R324Q; 11: *Pp*CaOMT (43.7 kDa); 12: *Pp*CaOMT D284C; 13: *Pp*CaOMT E311N; 14: *Pp*CaOMT D284C E311N; 15: *Pp*CaOMT Q337R; 16: *Pp*CaOMT D284C Q337R; 17: *Pp*CaOMT E311N Q337R; 18: *Pp*CaOMT D284C E311N Q337R.

<u>Amino acid alignment</u>

```
CLUSTAL O(1.2.4) multiple sequence alignment


MsCaOMT      MGSTGETQ-----------ITPTHISDEEANLFAMQLASASVLPMILKSALELDLLEIIA   49
RgANMT       MGSLSE------SHTQYKHGVEVEEDEEESYSRAMQLSMAIVLPMATQSAIQLGVFEIIA   54
CsANMT       MGSLSE------YQKL--AQKKHEEEEEESYSHAMQLAMGVVLPMATQAAIQLGVFEIIA   52
PpCaOMT      MASSLERKSHPKINHAEPEDEITKEEEDESFCYAMQLVGSSVLSMSLQSAIKLGIFDIIA   60
CsCaOMT      ------------------MDSIVDGERDQSFAYASQLVMGTVLPMAIQAVYELGIFEILD   42
                                        :::   * **  . ** *  : . :*.:::*:

MsCaOMT      KAGPGAQISPIEIASQLPT-TNPDAPVMLDRMLRLLACYIILTCSVRTQQDG-KVQRLYG   107
RgANMT       K-APGGRLSASEIATILQA-QNPKAPVMLDRMLRLLVSHRVLDCSVSG----PAGERLYG   108
CsANMT       K-A--GELSAPEIAAQLQA-QNVKAPMMLDRMLRLLVSHRVLECSVSG------GERLYA   102
PpCaOMT      RKGPGAKLSSSEIATKIGT-ENPEAPVMVDRILRLLTSHSVLNCSAVAANGGSDFQRVYS   119
CsCaOMT      KVGPGAKLCASDIAAQLLT-KNKDAPMMLDRILRLLASYSVVECSLDA----SGARRLYS   97
               .    :   ::*: : :  * .** *:**:****..: :: *            * *.

MsCaOMT      LATVAKYLVKNEDGVSISALNLMNQDKVLMESWYHLKDAVLDGGIPFNKAYGMTAFEYHG   167
RgANMT       LTSVSKYFVPDQDGASLGNFMALPLDKVFMESWMGVKGAVMEGGIPFNRVHGMHIFEYAS   168
CsANMT       LNPVSKYFVSNKDGASLGHFMALPLDKVFMESWLGLKDAVMEGGIPFNRVHGMHIFEYAS   162
PpCaOMT      LGPVSKYFVNDEEGGSLGPLLTLIQDRVFLESWSQLKDAVVEGGIPFNRVHGMHAFEYPG   179
CsCaOMT      LNSVSKYYVPNKDGVLLGPLLQMNQDKVLLESWSQLKDAILEGGIPFNRAHGVHVFEYAG   157
                *.*: . :::*  :. :  :  *:*::***  :*.*:::******:.:*:  *** .

MsCaOMT      TDPRFNKVFNKGMSDHSTITMKKILETYTGFE--GLKSLVDVGGGTGAVINTIVSKYPTI   225
RgANMT       SNSKFSDTYHRAMFNHSTIALKRILEHYKGFE--NVTKLVDVGGGLGVTLSMIASKYPHI   226
CsANMT       GNPRFNETYHEAMFNHSTIAMERILEHYEGFQ--NVERLVDVGGGFGVTLSMITSKYPQI   220
PpCaOMT      LDPRFNQVFNTAMFNHTTIVIKKLLHIYKGLEDKNLTQLVDVGGGLGVTLNLITSRYQHI   239
CsCaOMT      LDPKFNKHFNTAMYNYTSLVMSNILESYKGFD--NIKQLVDVGGSLGITLQAITTKYPYI   215
              : :*.  :: .* ::: :  ..:*. * *::  .:  ******. * .:  *.::*  *

MsCaOMT      KGINFDLPHVIEDAPSYPGVEHVGGDMFVSIPKADAVFMKWICHDWSDEHCLKFLKNCYE   285
RgANMT       QAINFDLPHVVQDAASYPGVEHVGGNMFESVPEGDAILMKWILHCWDDEQCLRILKNCYK   286
CsANMT       KAVNFDLPHVVQDAPSYAGVEHVGGNMFESVPEGDAILMKWILHCWDDDHCLRILKNCYK   280
PpCaOMT      KGINFDLPHVVNHAPSYPGVEHVGGDMFASVPSGDAIFMKWILHDWSDEHCLKLLKNCYK   299
CsCaOMT      KGINFDQPHVIDHAPSHPRIEHVGGDMFQSVPKGDAIIMKSVLHDWNDEHCLKLLKNCYK   275
             :.:*** ***:..* ..  : ****:** .:*..*:::** : * *.*::*  :*****.

MsCaOMT      ALPDNGKVIVAECILPVAPDSSLATKGVVHIDVIMLAHNPGGKERTQKEFEDLAKGAGFQ   345
RgANMT       ATPENGKVIVMNSVVPETPEVSSSARETSLLDVLLMTRDGGGRERTQKEFTELAIGAGFK   346
CsANMT       AVPGNGKVIVMNSIVPEIPEVSSAARETSLLDVLLMTRDGGGRERTKKEYTELAIAAGFK   340
PpCaOMT      AIPDNGKVIVVEALLPAMPETSTATKTTSQLDVLMMTQNPGGKERSEQEFMALATGAGFS   359
CsCaOMT      SIPEDGKVIVVESMLPEVPNTSIESKSNSHFDVLMMIQSPGGKERTRHEFMTLATGAGFG   335
             : * .***:: :.::*  *: .  ::    .*:::: :. **:** .:*:  ** .***

MsCaOMT      GFKVHCNAFNTYIMEFLKKV           365
RgANMT       GINFACCVCNLHIMEFFK--           364
CsANMT       GINFASCVCNLYIMEFFK--           358
PpCaOMT      GIRYECFVCNFWVMEFFK--           377
CsCaOMT      GISCELAIGNLWVMEFYK--           353
             *.       *   :** *
```

Figure S2 – Amino acid alignment of the used MTs (*Rg*ANMT; *Pp*CaOMT) in comparison to *Cs*ANMT; *Ms*CaOMT and *Cs*CaOMT. The alignment was created with Clustal Omega.[1,2] The positions in yellow were replaced by the residue from the other enzyme family.

<u>Experimental data</u>

pH screening

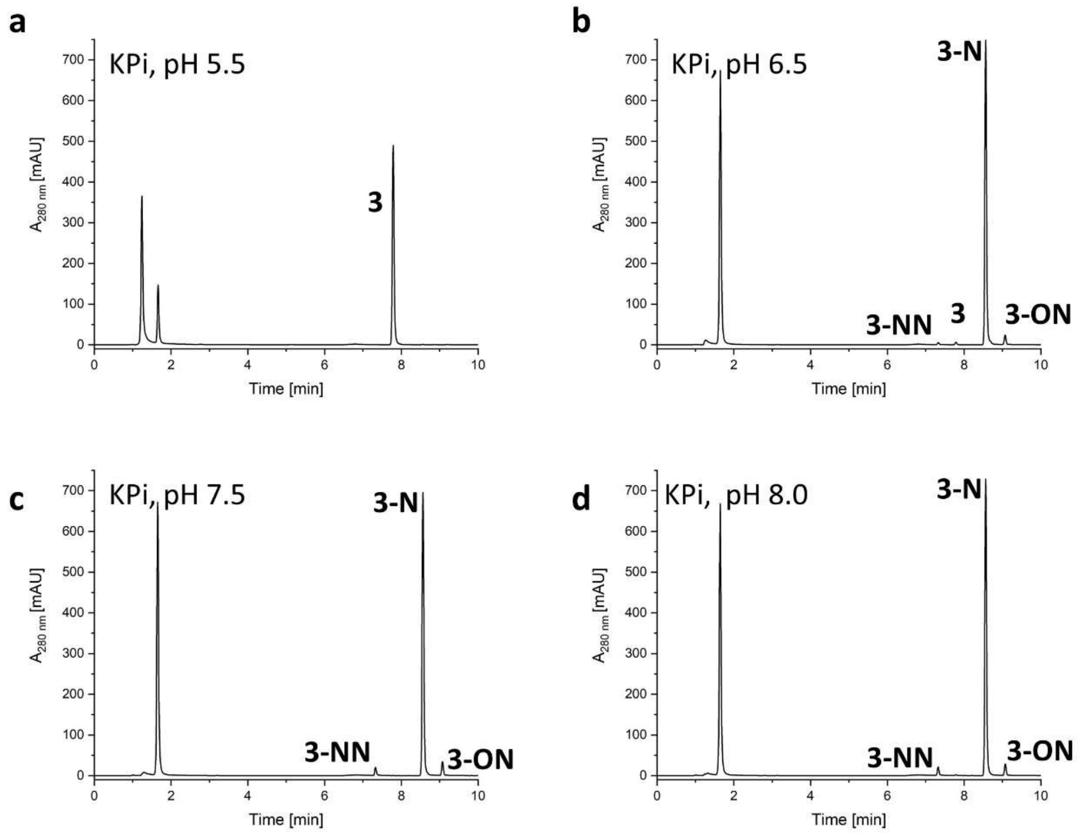

Figure S3 – pH screening HPLC. The reaction was catalysed by *Rg*ANMT using substrate **3**. a: KPi buffer; pH 5.5. b: KPi buffer; pH 6.5. c: KPi buffer; pH 7.5. d: KPi buffer; pH 8.0.

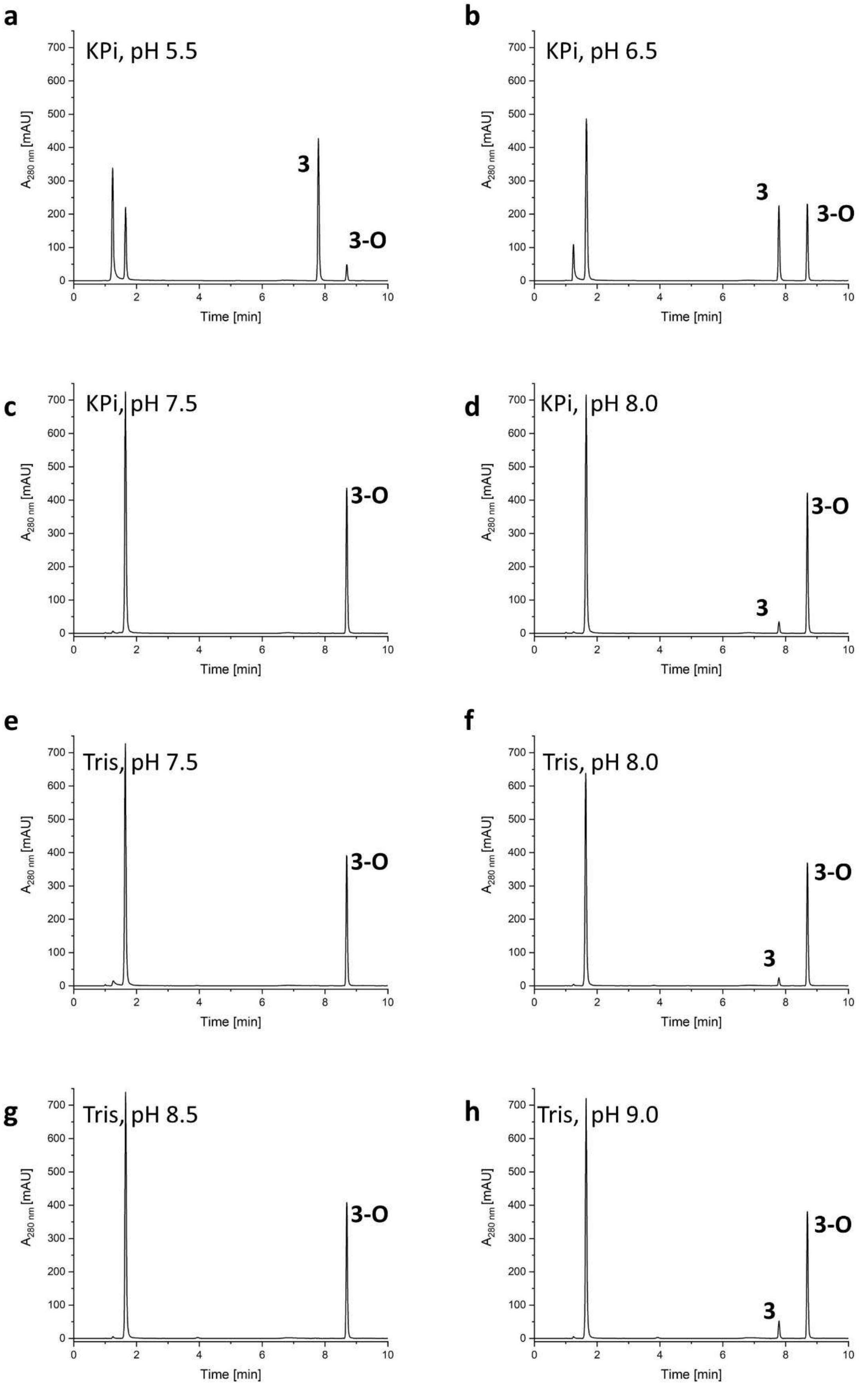

Figure S4 – pH screening HPLC. The reaction was catalysed by *Pp*CaOMT using substrate **3**. a: KPi buffer; pH 5.5. b: KPi buffer; pH 6.5. c: KPi buffer; pH 7.5. d: KPi buffer; pH 8.0. e: Tris buffer; pH 7.5. f: Tris buffer; pH 8.0. g: Tris buffer; pH 8.5. h: Tris buffer; pH 9.0.

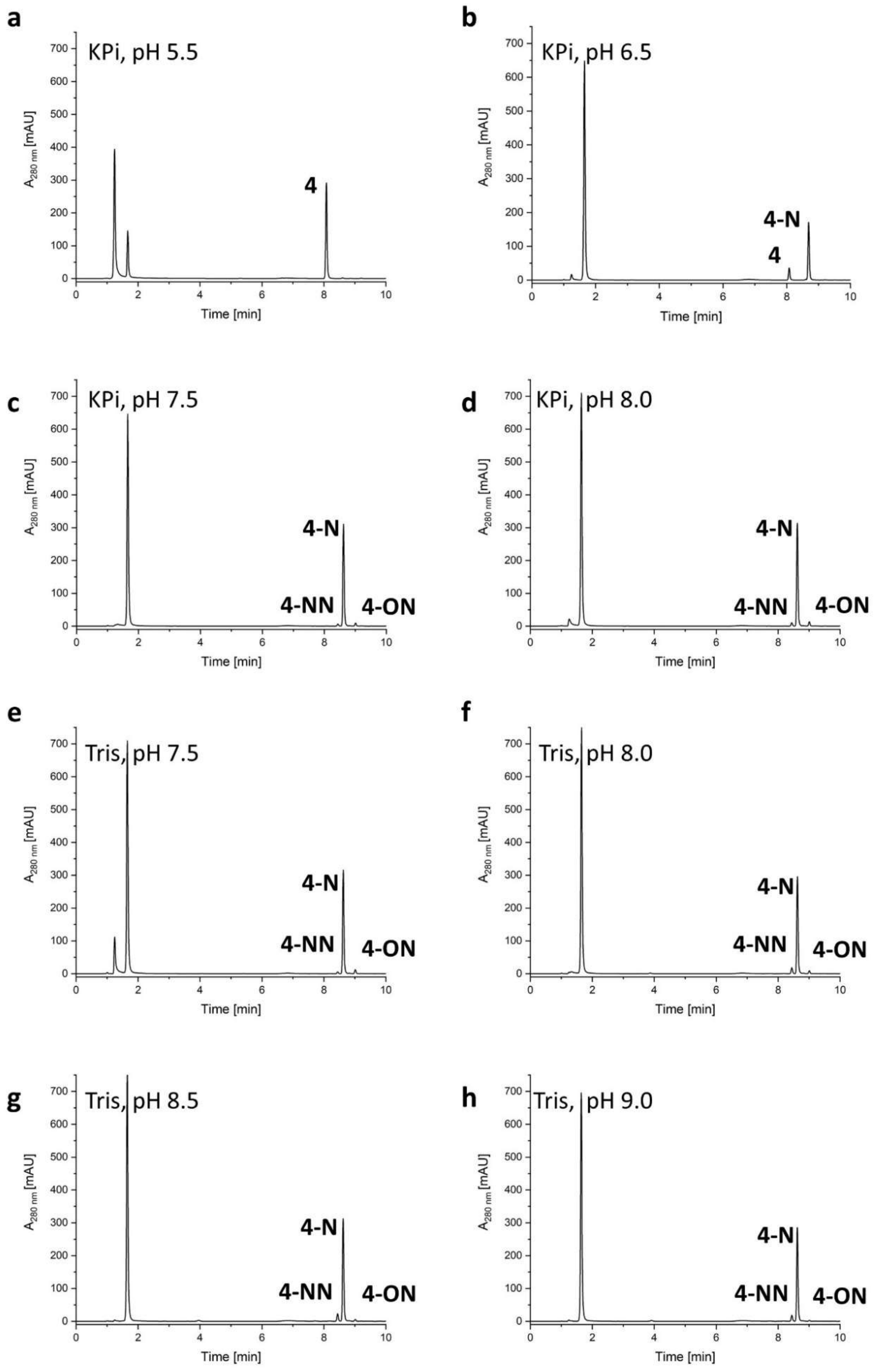

Figure S5 – pH screening HPLC. The reaction was catalysed by *Rg*ANMT using substrate **4**. a: KPi buffer; pH 5.5. b: KPi buffer; pH 6.5. c: KPi buffer; pH 7.5. d: KPi buffer; pH 8.0. e: Tris buffer; pH 7.5. f: Tris buffer; pH 8.0. g: Tris buffer; pH 8.5. h: Tris buffer; pH 9.0.

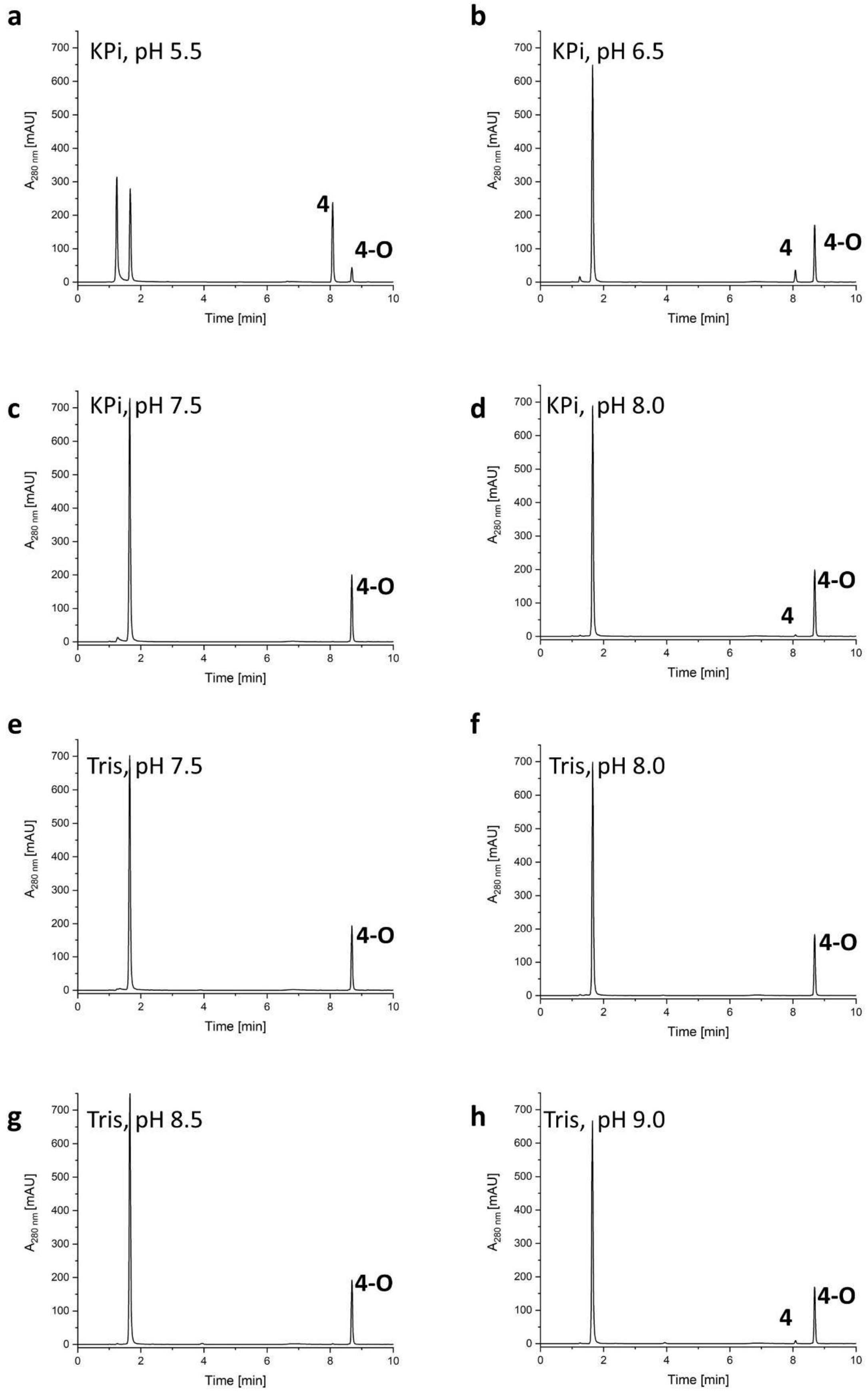

Figure S6 – pH screening HPLC. The reaction was catalysed by *Pp*CaOMT using substrate **4**. a: KPi buffer; pH 5.5. b: KPi buffer; pH 6.5. c: KPi buffer; pH 7.5. d: KPi buffer; pH 8.0. e: Tris buffer; pH 7.5. f: Tris buffer; pH 8.0. g: Tris buffer; pH 8.5. h: Tris buffer; pH 9.0.

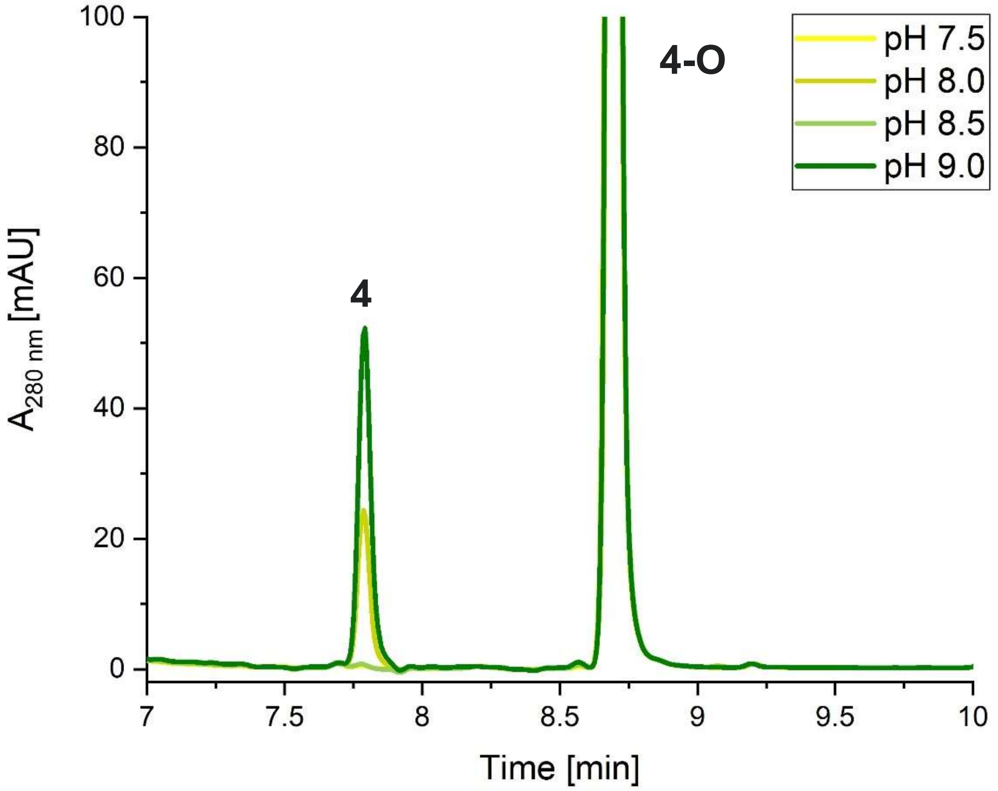

Figure S7 – Overlay of selected reactions with *Pp*CaOMT and substrate **4** (forming **4-O**) at pH value 7.5; 8.0; 8.5; 9.0 (Tris buffer).

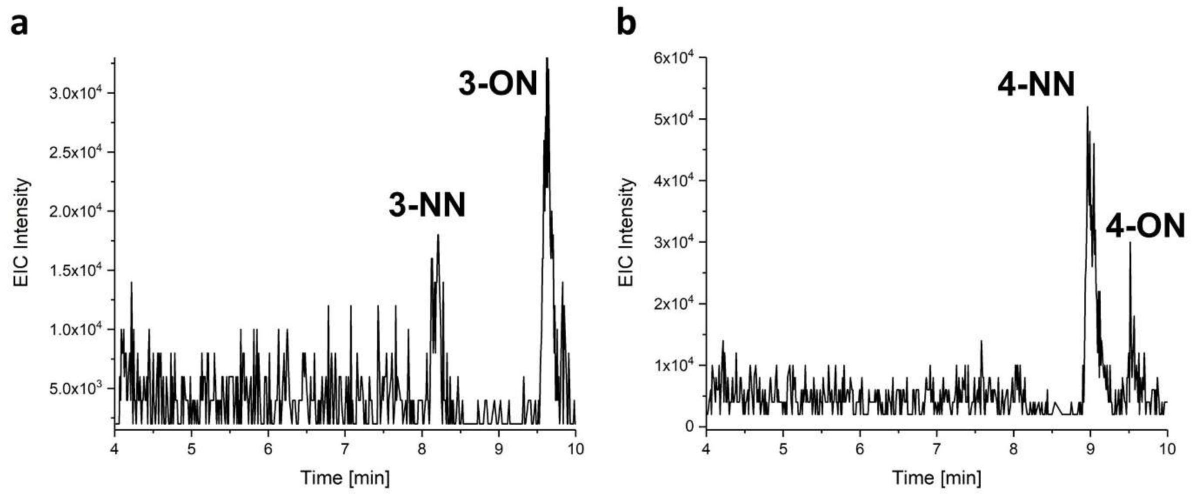

Figure S8 – Extracted ion chromatogram of reaction catalysed by *Rg*ANMT at pH 8.5 using substrate **3** (a) or **4** (b). Q1 measurements were performed in positive mode. Extracted mass: 183.07 Da.

Table S4 – Distribution of substrate **3** and **4** and all formed products at different pH values in Tris buffer using *Rg*ANMT as biocatalyst. (n.d.- not detected)

pH	**3** 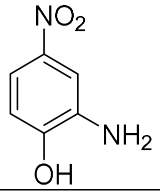		**3-N** 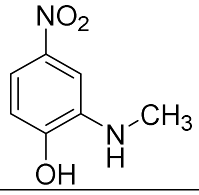		**3-NN** 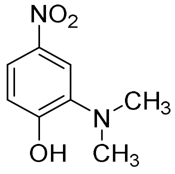		**3-ON** 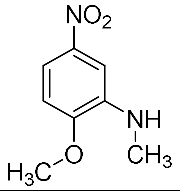		**t-test**
7.5	1%	±0.5%	95%	±1.1%	2%	±0.5%	3%	±1.3%	0.372
8.0	n.d.		92%	±0.6%	5%	±0.3%	3%	±0.2%	0.057
8.5	n.d.		90%	±0.4%	7%	±0.4%	3%	±0.04%	0.008
9.0	n.d.		90%	±0.3%	7%	±0.3%	3%	±0.04%	0.003
pH	**4** 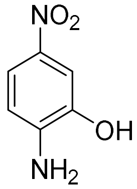		**4-N** 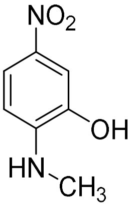		**4-NN** 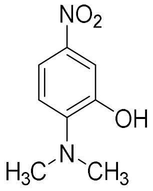		**4-ON** 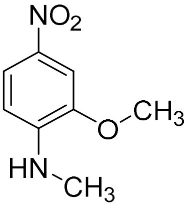		
7.5	n.d.		96%	±2.0%	1%	±0.9%	3%	±1.1%	0.321
8.0	n.d.		92%	±0.6%	5%	±0.5%	3%	±0.1%	0.033
8.5	n.d.		89%	±2.1%	9%	±1.6%	2%	±0.5%	0.055
9.0	n.d.		93%	±0.4%	6%	±0.2%	1%	±0.2%	0.003

Variant screening

Substrates: Aminonitrophenols

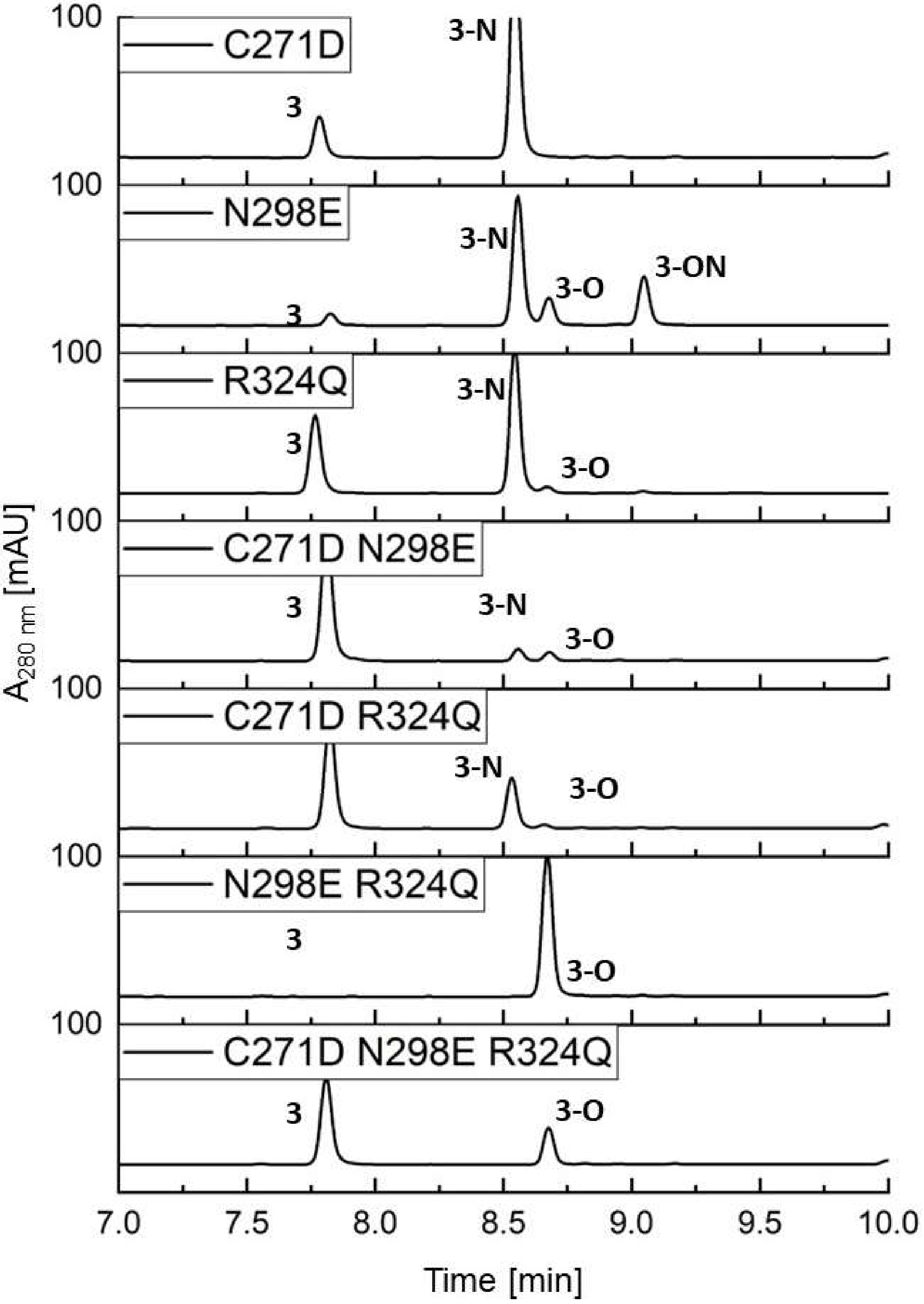

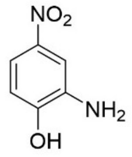

3

Figure S9 – Endpoint of time course experiment. Reactions catalysed by *Rg*ANMT variants using substrate **3**.

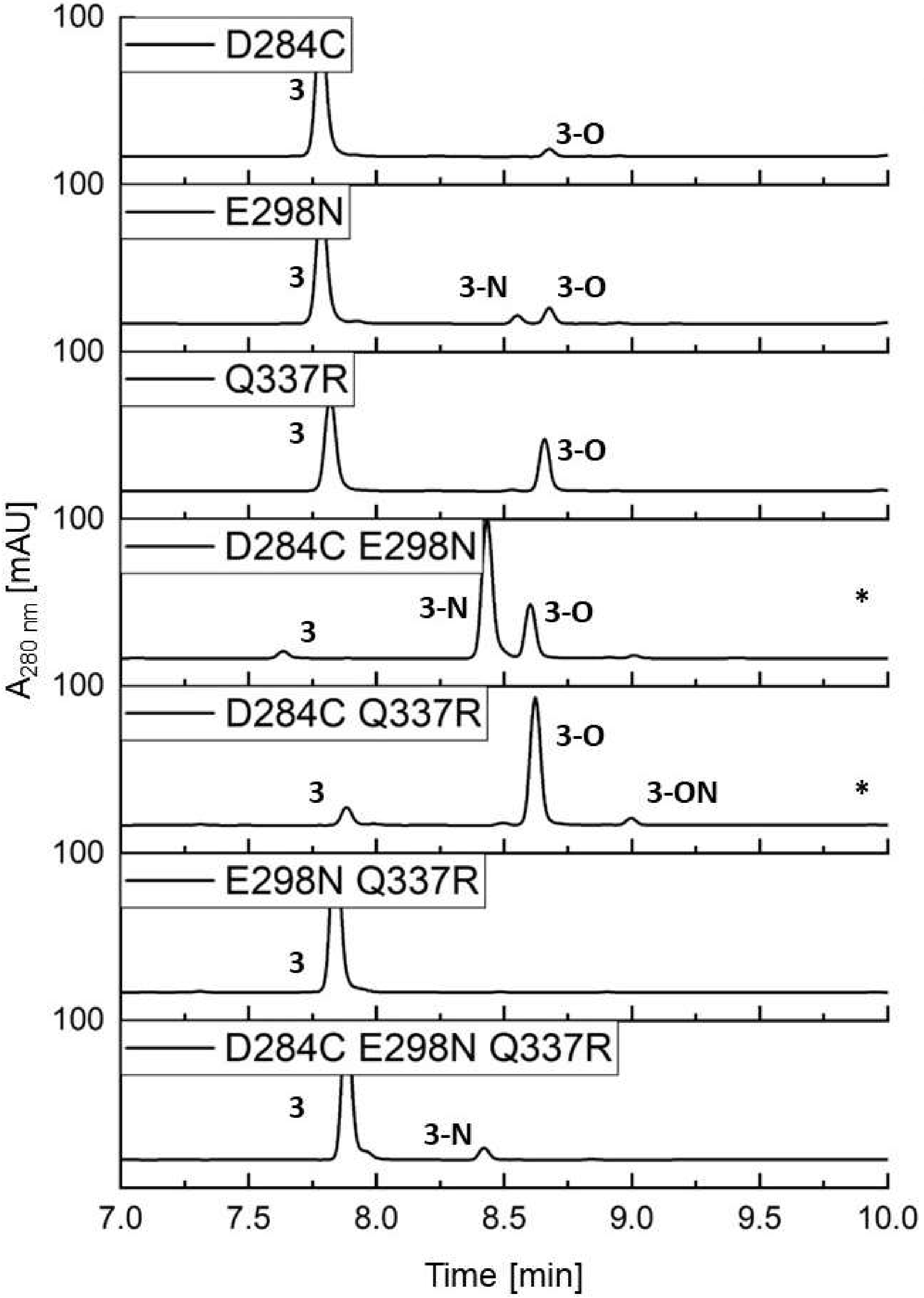

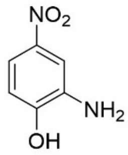

3

Figure S10 – Endpoint of time course experiment. Reactions catalysed by *Pp*CaOMT variants using substrate **3**. Chromatograms marked with * show a slight retention time shift due to pressure issues during measurements.

Substrates: Physiological and related substrates

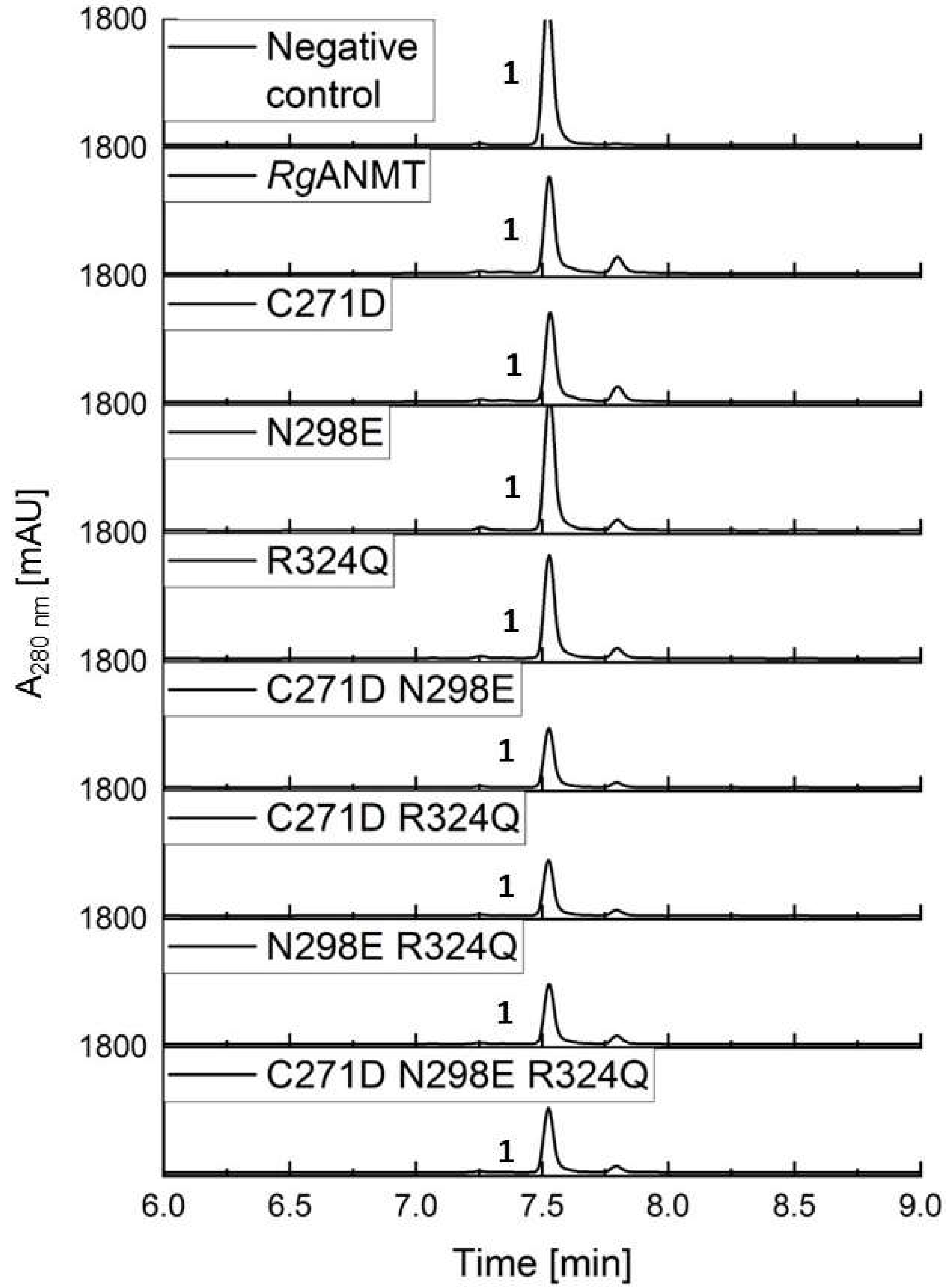

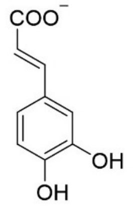

1

Figure S11 – Reactions catalysed by *Rg*ANMT variants using substrate **1**. The peak at 7.8 min remains unidentified. Nevertheless, methylation was excluded as possible reaction with ^{13}C-NMR analysis (see Figure S22). Samples were taken after 20 h.

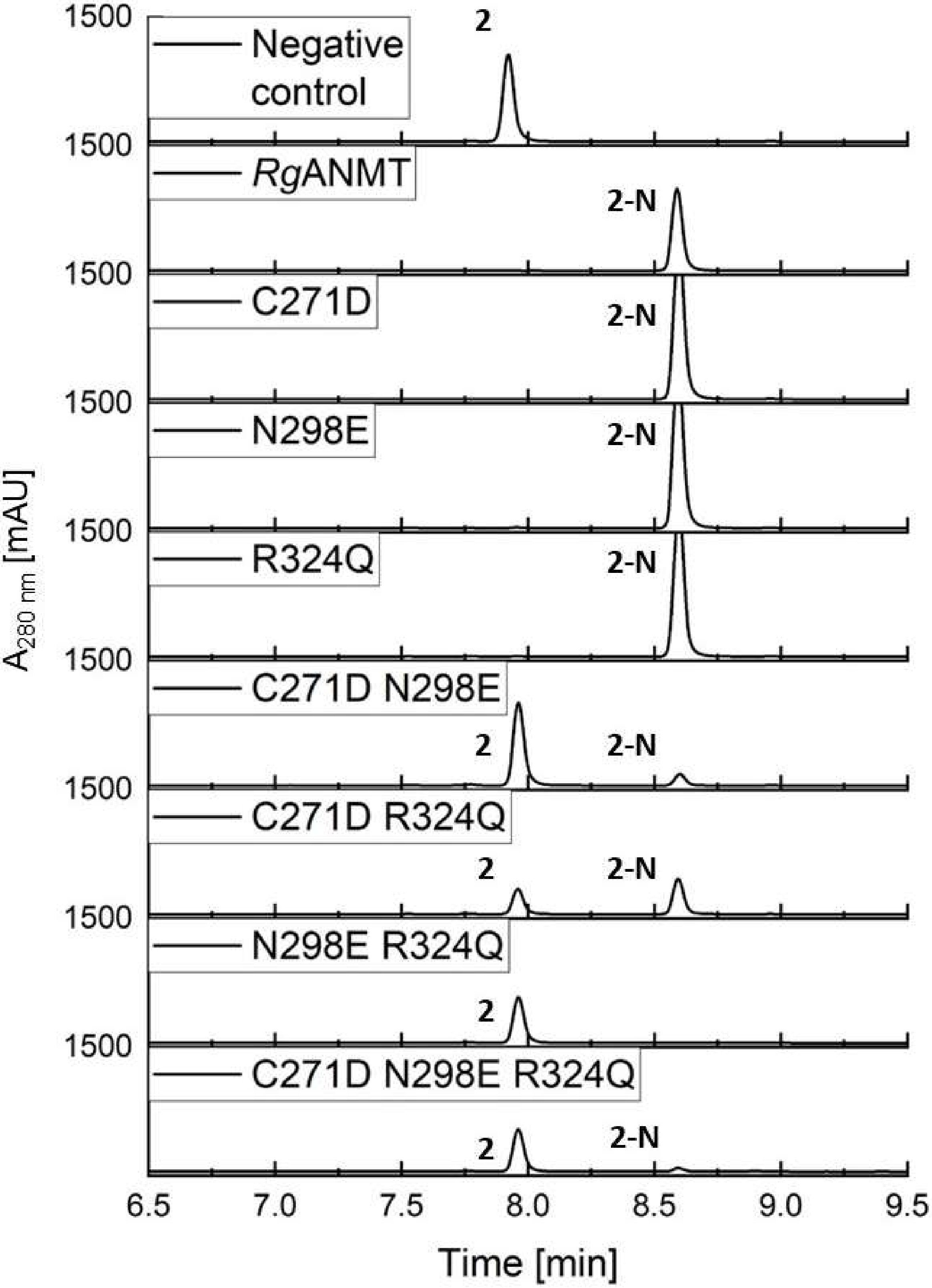

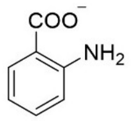

2

Figure S12 – Reactions catalysed by *Rg*ANMT variants using substrate **2**. Samples were taken after 20 h.

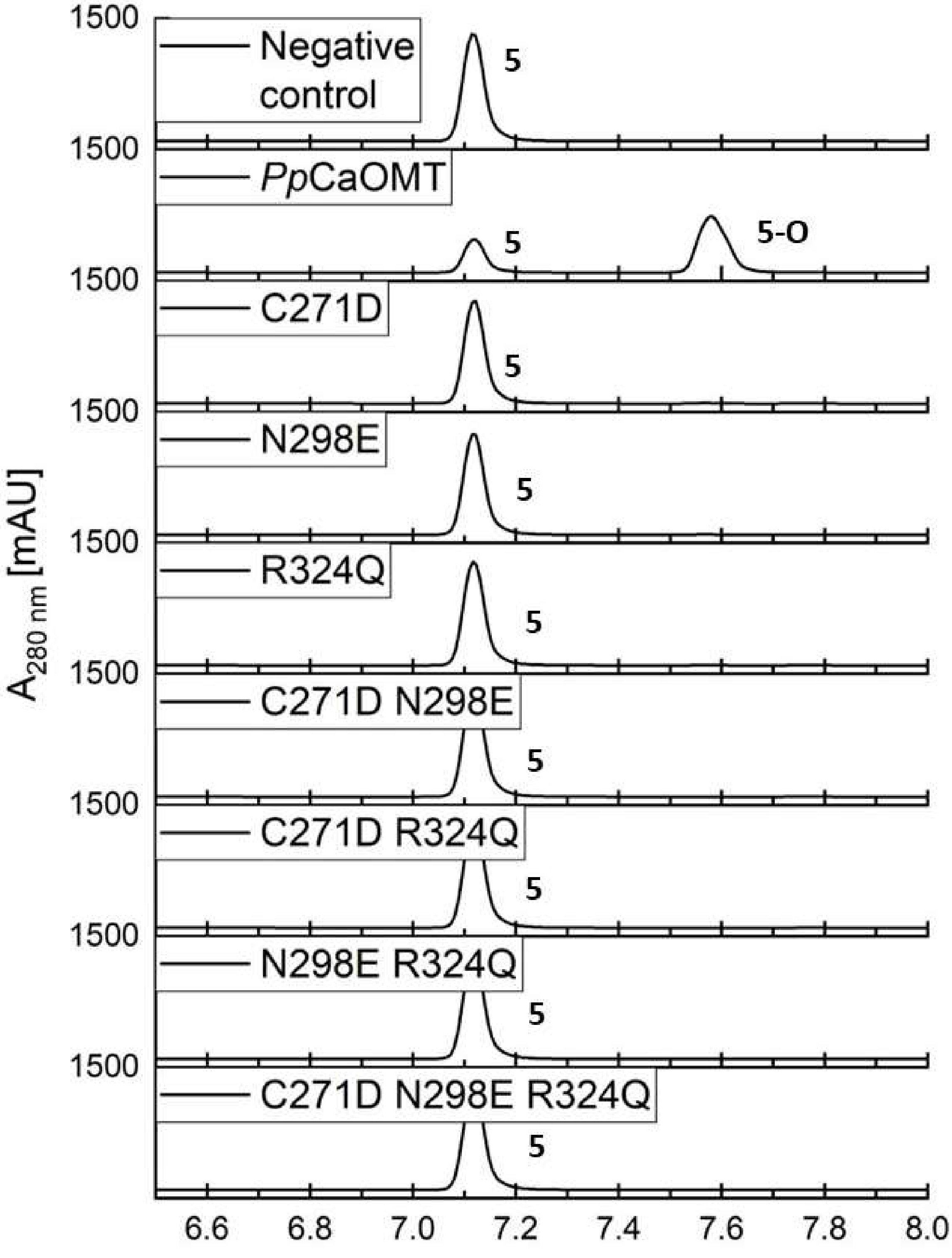

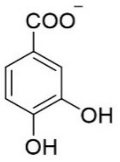

5

Figure S13 – Reactions catalysed by *Rg*ANMT variants (and *Pp*CaOMT as positive control) using substrate **5**. Samples were taken after 20 h.

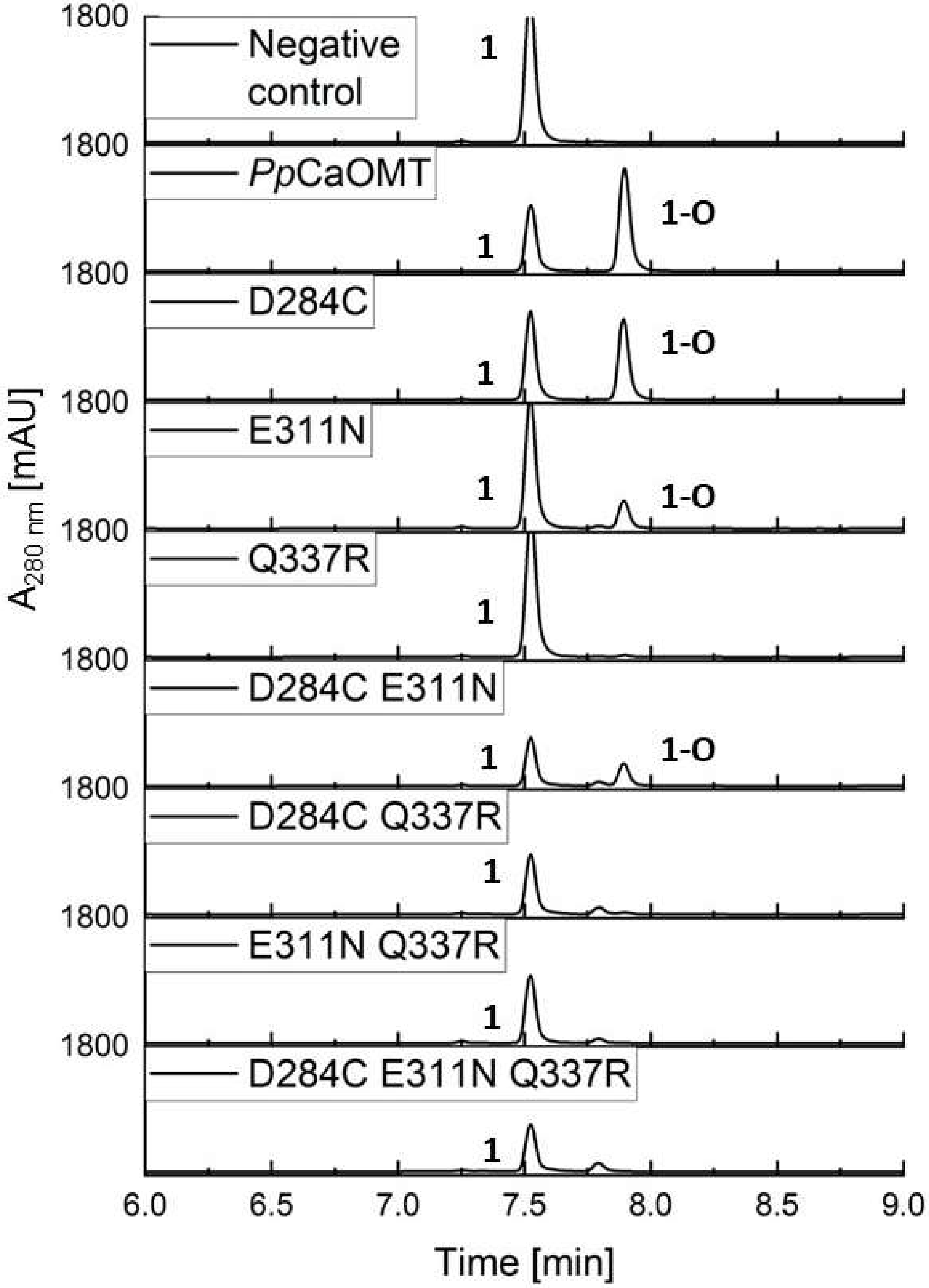

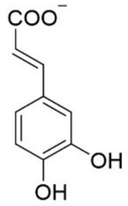

1

Figure S14 – Reactions catalysed by *Pp*CaOMT variants using substrate 1. The peak at 7.8 min remains unidentified. Though, methylation was excluded as possible reaction using ^{13}C NMR analysis (see Figure S22). Samples were taken after 20 h.

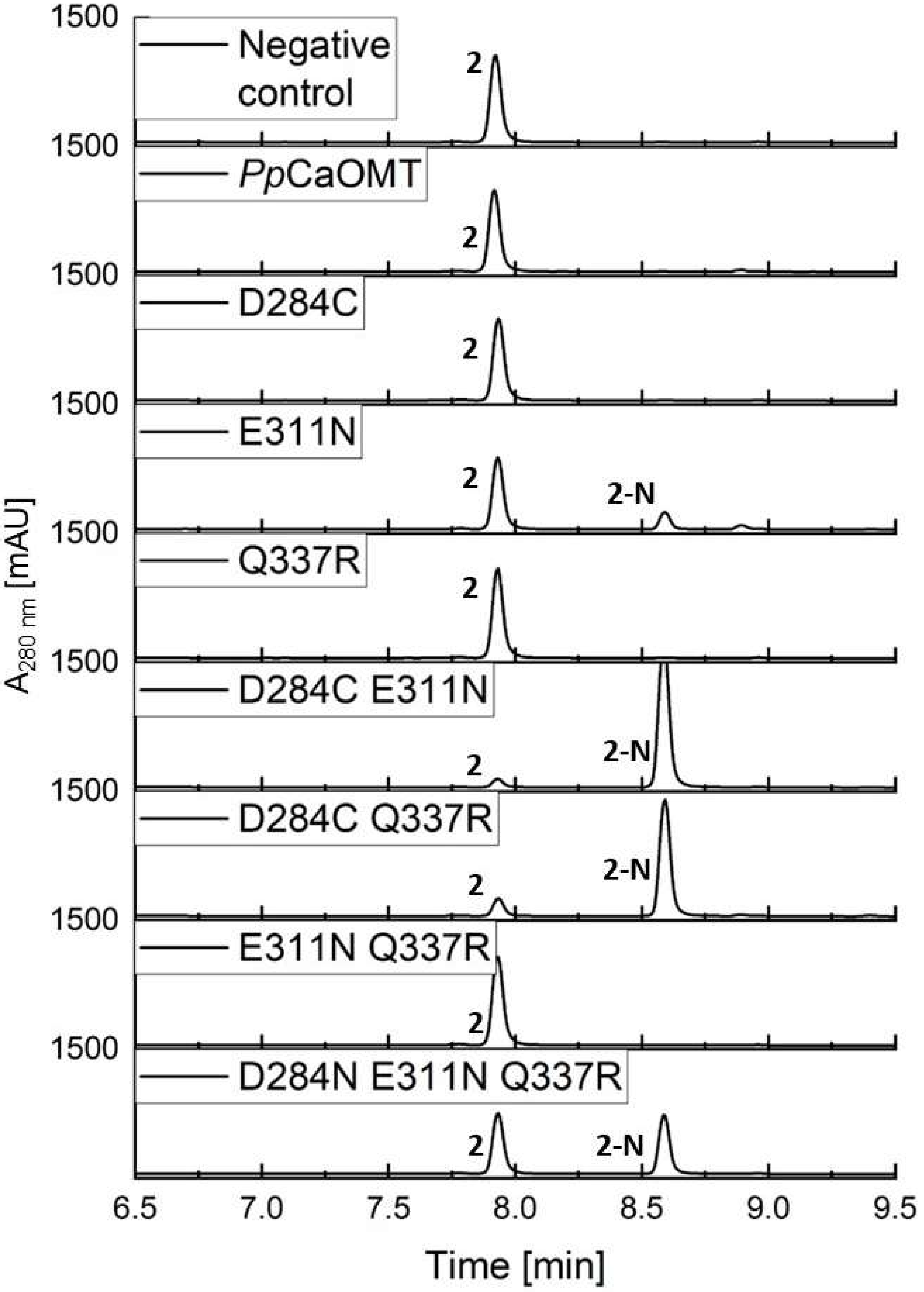

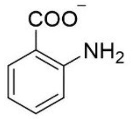

2

Figure S15 – Reactions catalysed by *Pp*CaOMT variants using substrate **2**. Samples were taken after 20 h.

Additional substrates
Several aminobromophenols (substrates **6** – **9**) with the respective substituents in different positions were incubated with enzyme variants 2O, 4O, and 7O, as well as variants 2N and 6N.

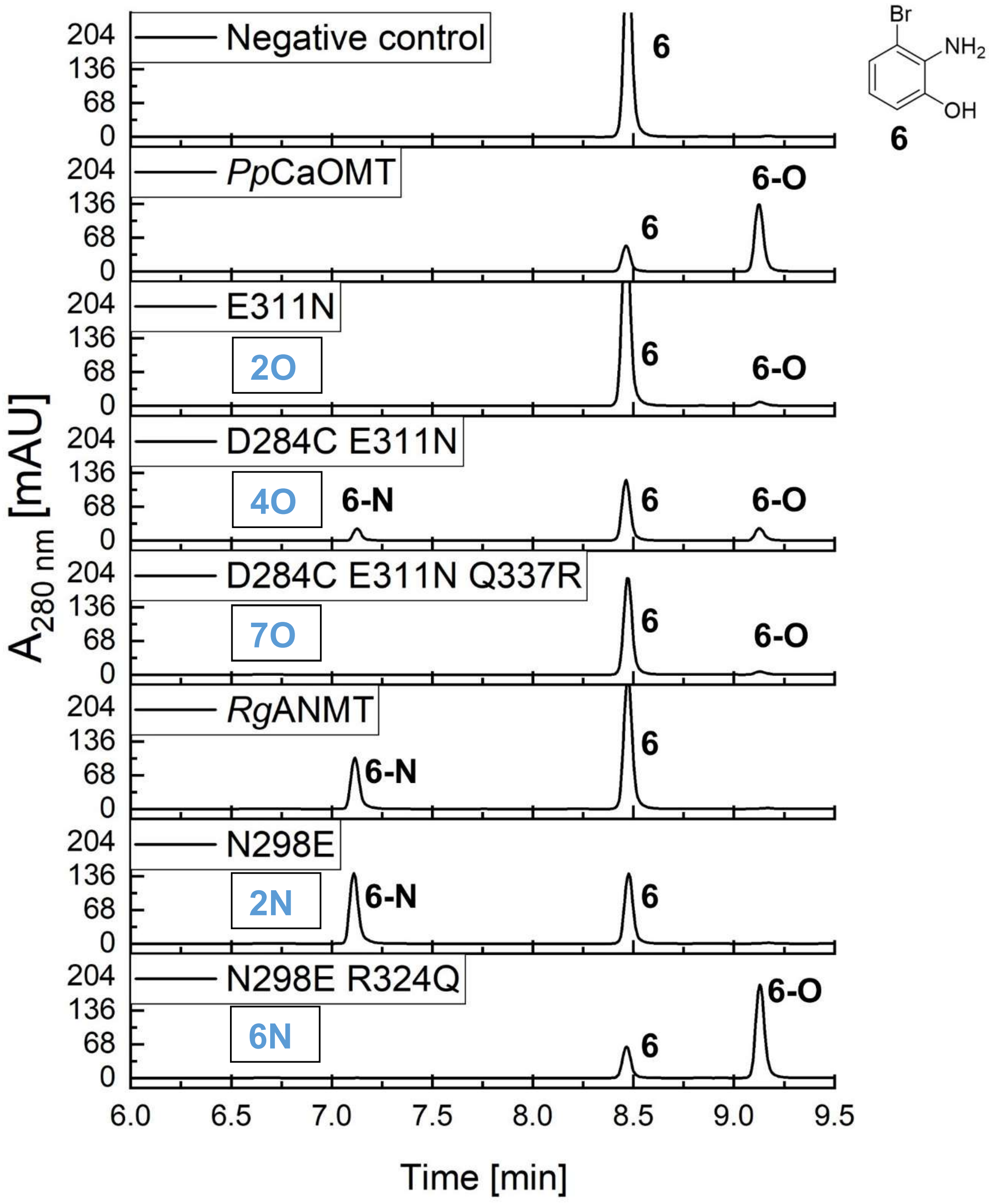

Figure S16 – Reactions catalysed by *Pp*CaOMT and *Rg*ANMT (variants) using substrate **6**. Samples were taken after 20 h.

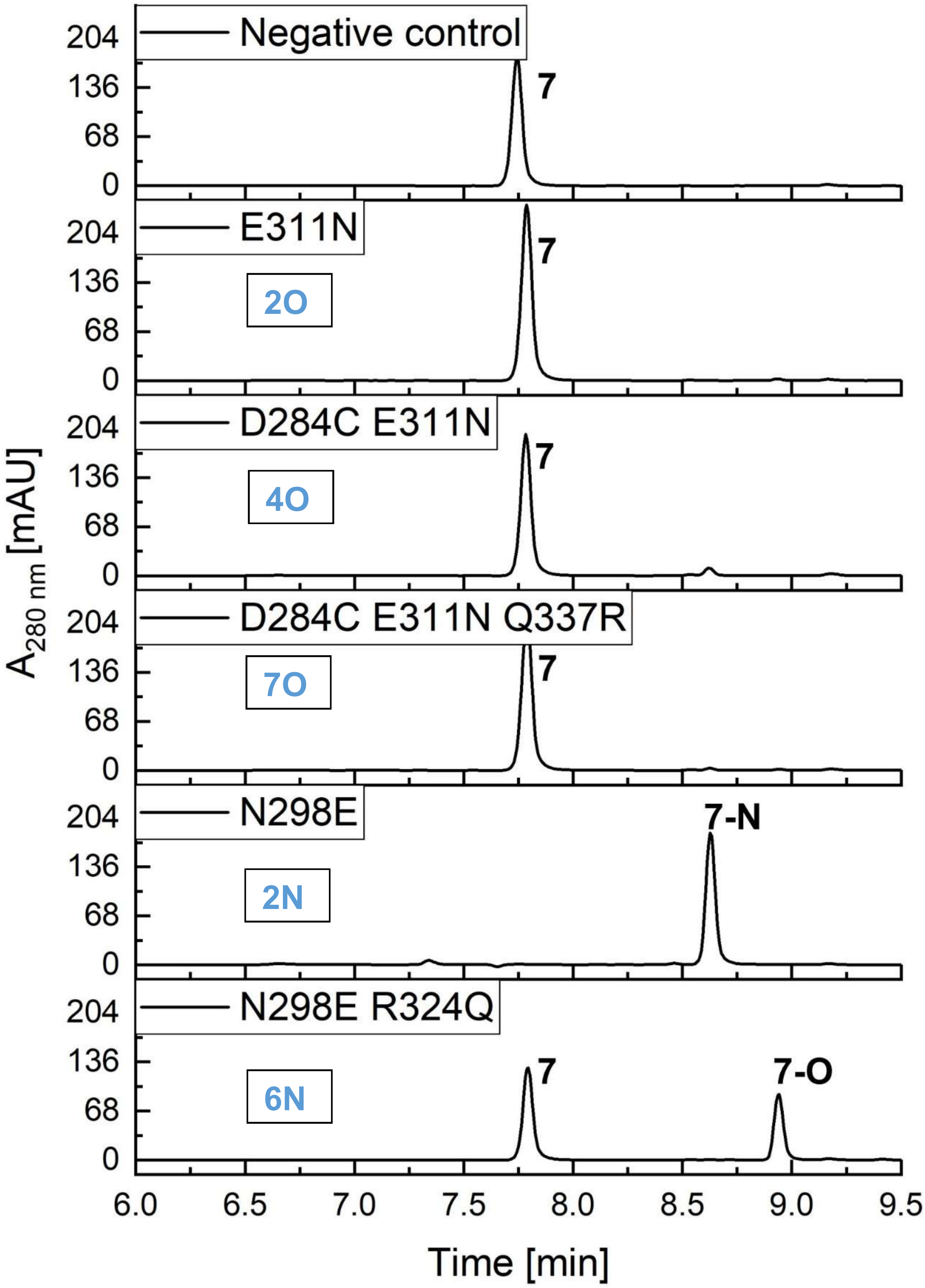

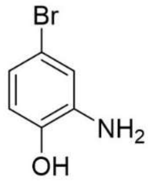

7

Figure S17 – Reactions catalysed by *Pp*CaOMT and *Rg*ANMT variants (2O, 4O, 7O; 2N, 6N) using substrate **7**. Reactions with the wildtype enzymes had been analysed in a previous study, for both enzymes the substrate were converted less then 20%.[3] While the variants of the *O*-MT do not lead to substantial conversion, both the *N*- and *O*-methylated product can be generated using the *Rg*ANMT variants 2N and 6N, respectively. Samples were taken after 20 h.

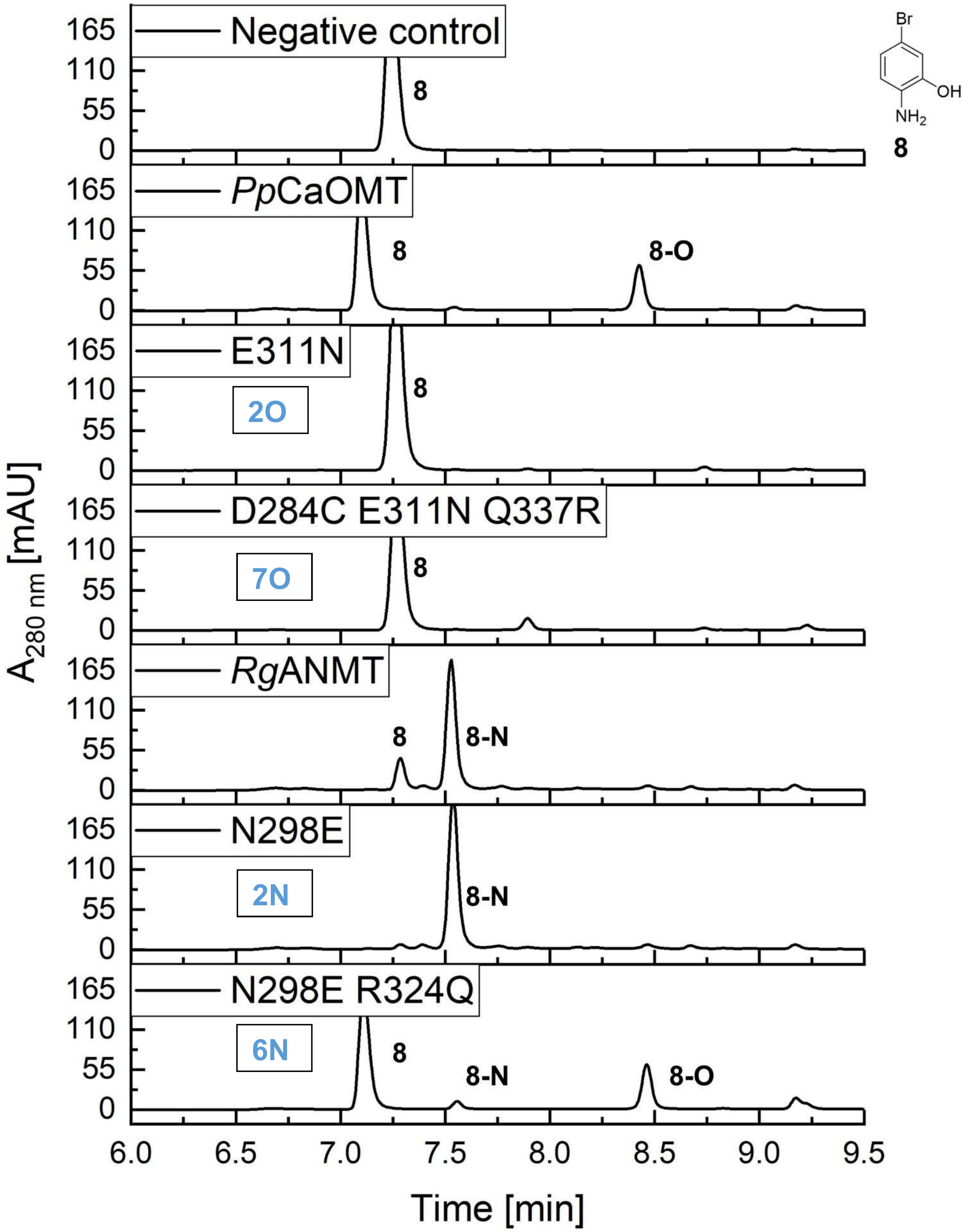

Figure S18 – Reactions catalysed by *Pp*CaOMT and *Rg*ANMT (variants) (2O, 7O; 2N, 6N) using substrate **8**. Samples were taken after 20 h.

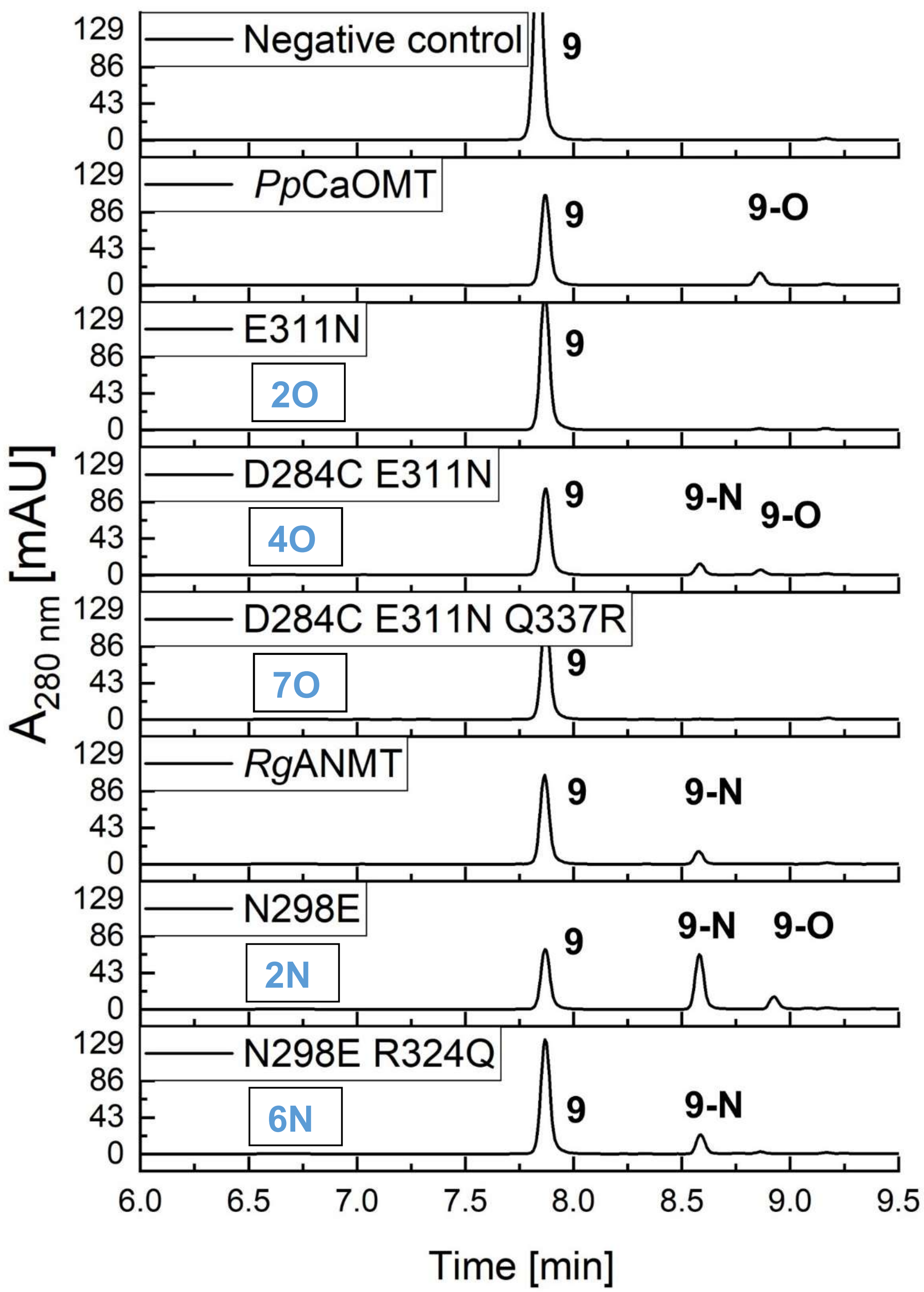

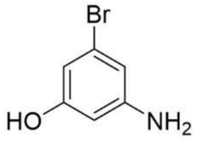

9

Figure S19 – Reactions catalysed by *Pp*CaOMT and *Rg*ANMT (variants) (2O, 4O, 7O; 2N, 6N) using substrate **9**. Samples were taken after 20 h.

N- and *O*-methylated products of substrates **7** and **8** (**7-N, 7-O; 8-N, 8-O)** had been identified in previous studies. Since there were no methylated standards available for substrates **6** and **9**, NMR analysis was used to identify the peaks referring to the *N*- or *O*-methylated products **6-N**/**6-O** and **9-N**/**9-O** (Figure S23 and S24).

NMR analysis

Confirmation of dimethylation at amino group, catalysed by RgANMT (pH screening)

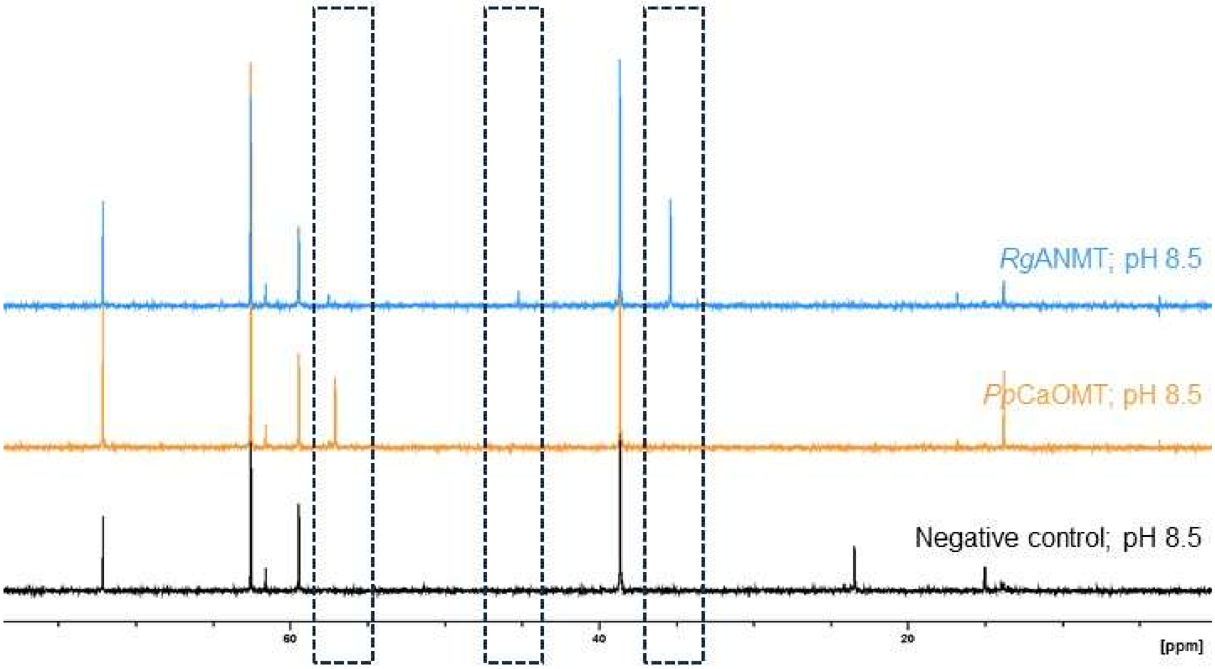

Figure S20 – ^{13}C NMR analysis of reaction with substrate **3** at pH 8.5 (using ^{13}C labeled methionine). The signal at 36 ppm represents the carbon attached to the nitrogen (blue line; *Rg*ANMT reaction, product **3-N**). The signal at 45 ppm shows the second carbon attached to the nitrogen (*Rg*ANMT reaction, product **3-NN**). In the reaction catalysed by *Pp*CaOMT, the carbon signal appears at 59 ppm (attached to the oxygen, product **3-O**). This signal is also present in a small intensity in the reaction catalysed by *Rg*ANMT, stemming from product **3-ON**. All other signals are explained in Table S3 in the NMR chapter.

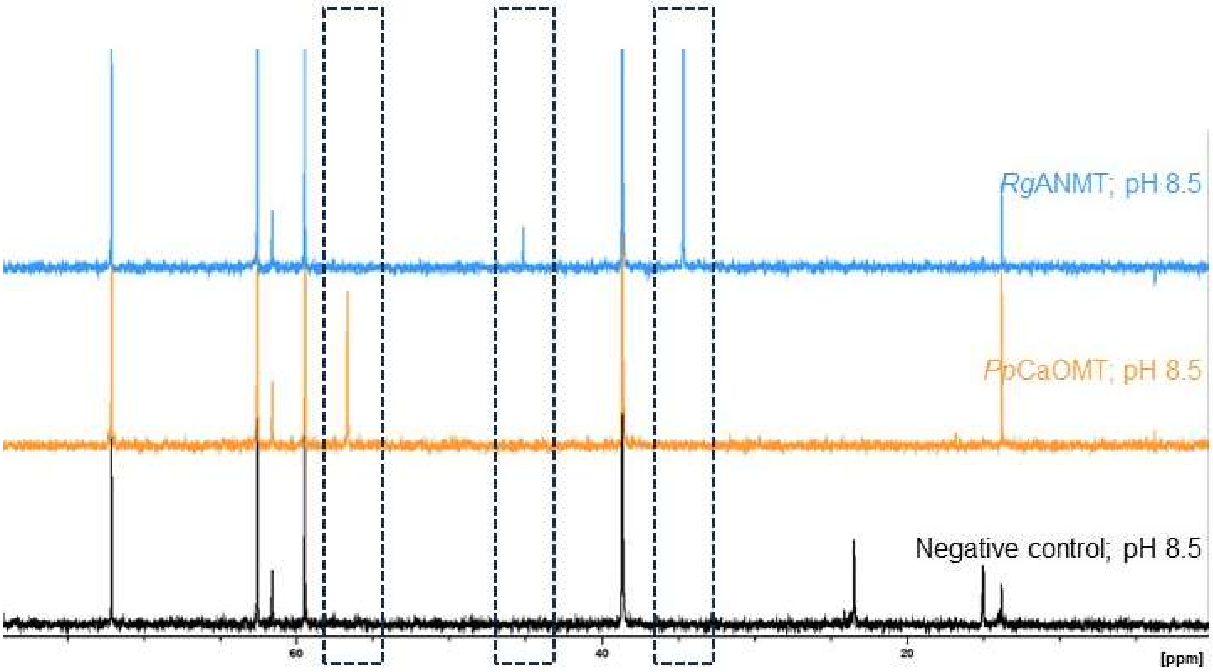

Figure S21 – ^{13}C NMR analysis of reaction with substrate **4** at pH 8.5 (using ^{13}C labeled methionine). The signal at 36 ppm was assigned to the carbon attached to the nitrogen (blue line; *Rg*ANMT reaction) (Product **4-N**). The signal at 45 ppm shows the second carbon attached to the nitrogen (*Rg*ANMT reaction, product **4-NN**). In the reaction catalysed by *Pp*CaOMT, the carbon signal appears at 59 ppm due (methyl attached to the oxygen, product **4-O**). All other signals are explained in Table S3 in the NMR chapter.

*Investigation of unidentified peak from variant assay with substrate **1***

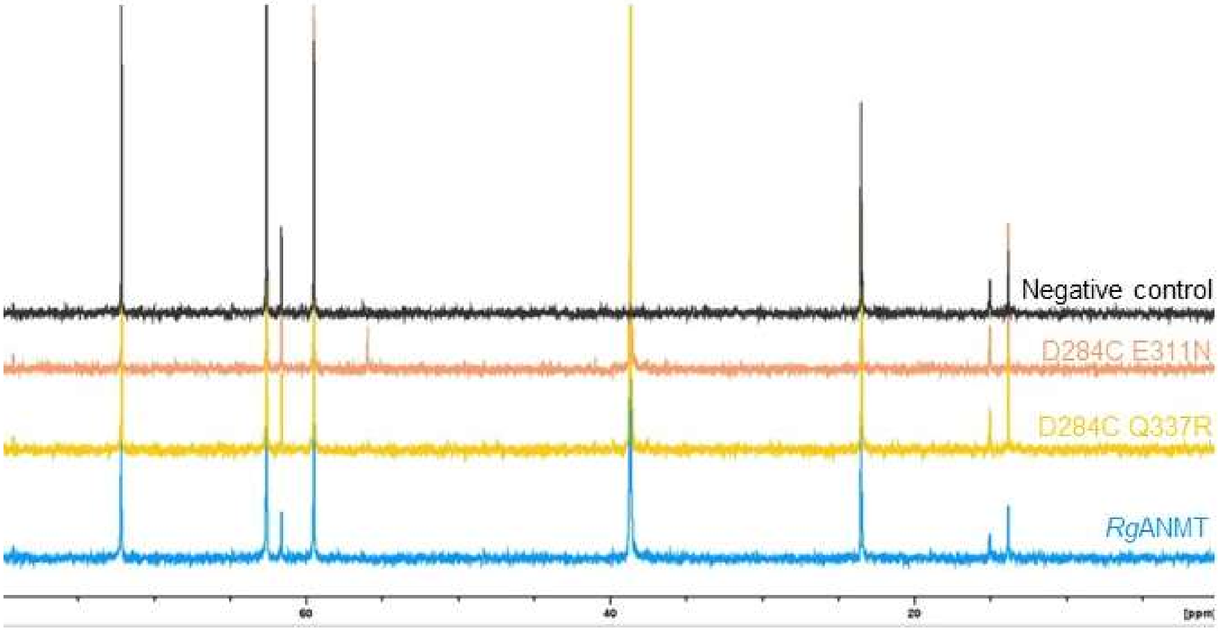

Figure S22 – ^{13}C NMR analysis of reaction with substrate **1** to investigate the unknown peak from the HPLC chromatograms (Figure S11 and S14). In the reaction catalysed by *Pp*CaOMT 4O (D284C E311N), there is a signal appears at 57 ppm (product **1-O**). Other than that, there are no signals for other methylated products found. All other signals are explained in Table S3 in the NMR chapter.

Confirmation of N-*methylated products*

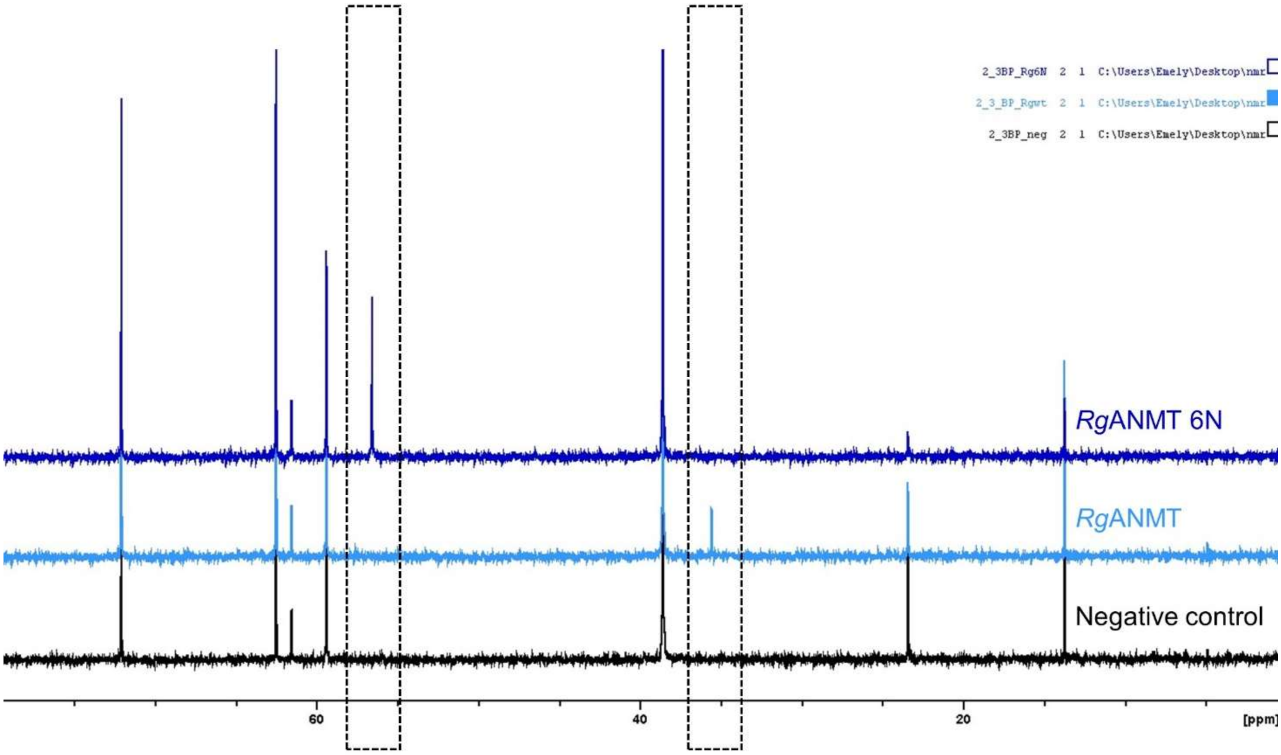

Figure S23 – ^{13}C NMR analysis of reaction with substrate **6** catalysed by *Rg*ANMT wildtype or *Rg*ANMT variant 6N (N298E R324Q). No methylated standards were commercially available. Peaks from HPLC chromatograms (Figure S16) were identified through NMR analysis. The methylation in *N*-position was confirmed in the *Rg*ANMT catalysed reaction (light blue) by the signal at 36 ppm. The chemoselectivity was different in the reaction catalysed by variant 6N in *O*-position [(dark blue) 58 ppm].

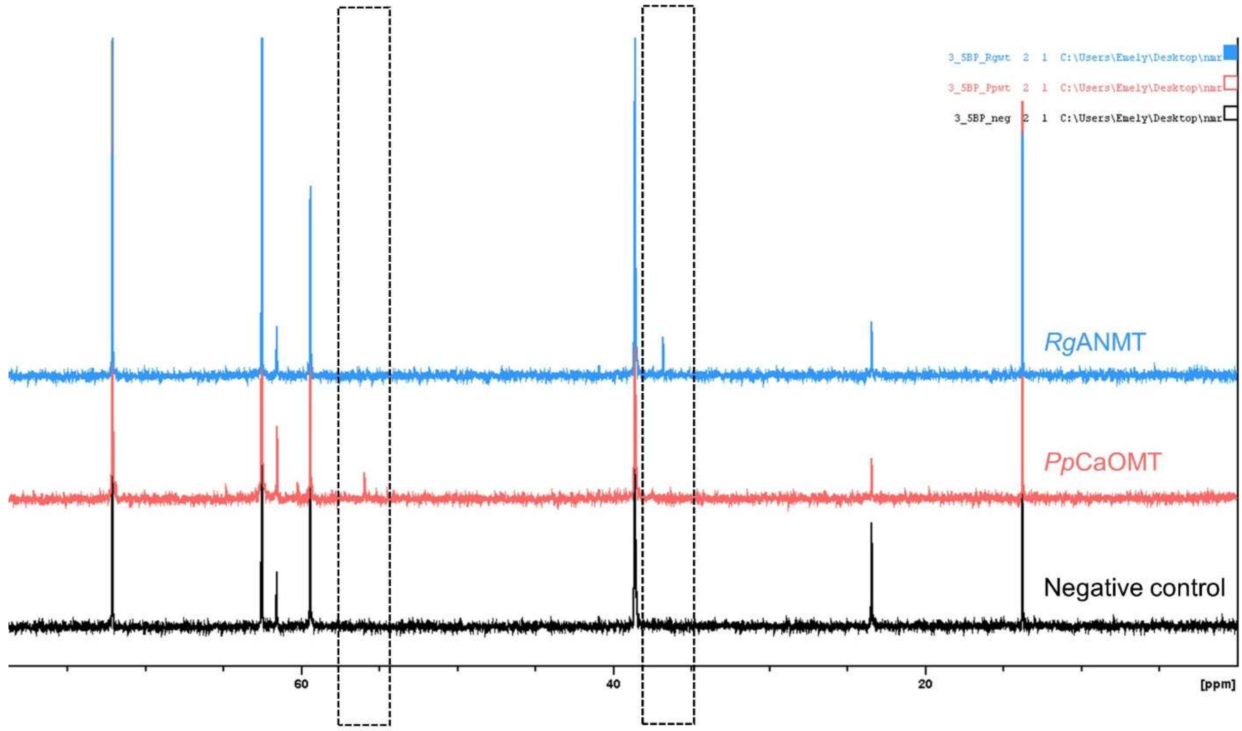

Figure S24 – ^{13}C NMR analysis of reaction with substrate **9** using *Pp*CaOMT and *Rg*ANMT wildtype enzymes. No methylated standards were commercially available. Peaks from HPLC chromatograms (Figure S19) were identified through NMR analysis. The methylation in *N*-position was confirmed in the *Rg*ANMT catalysed reaction (light blue) by the signal at 36 ppm. The chemoselectivity was different in the reaction catalysed by *Pp*CaOMT in *O*-position [(red) 58 ppm].

Computational modelling

Preliminary computational analysis of substrate-bound MD simulations

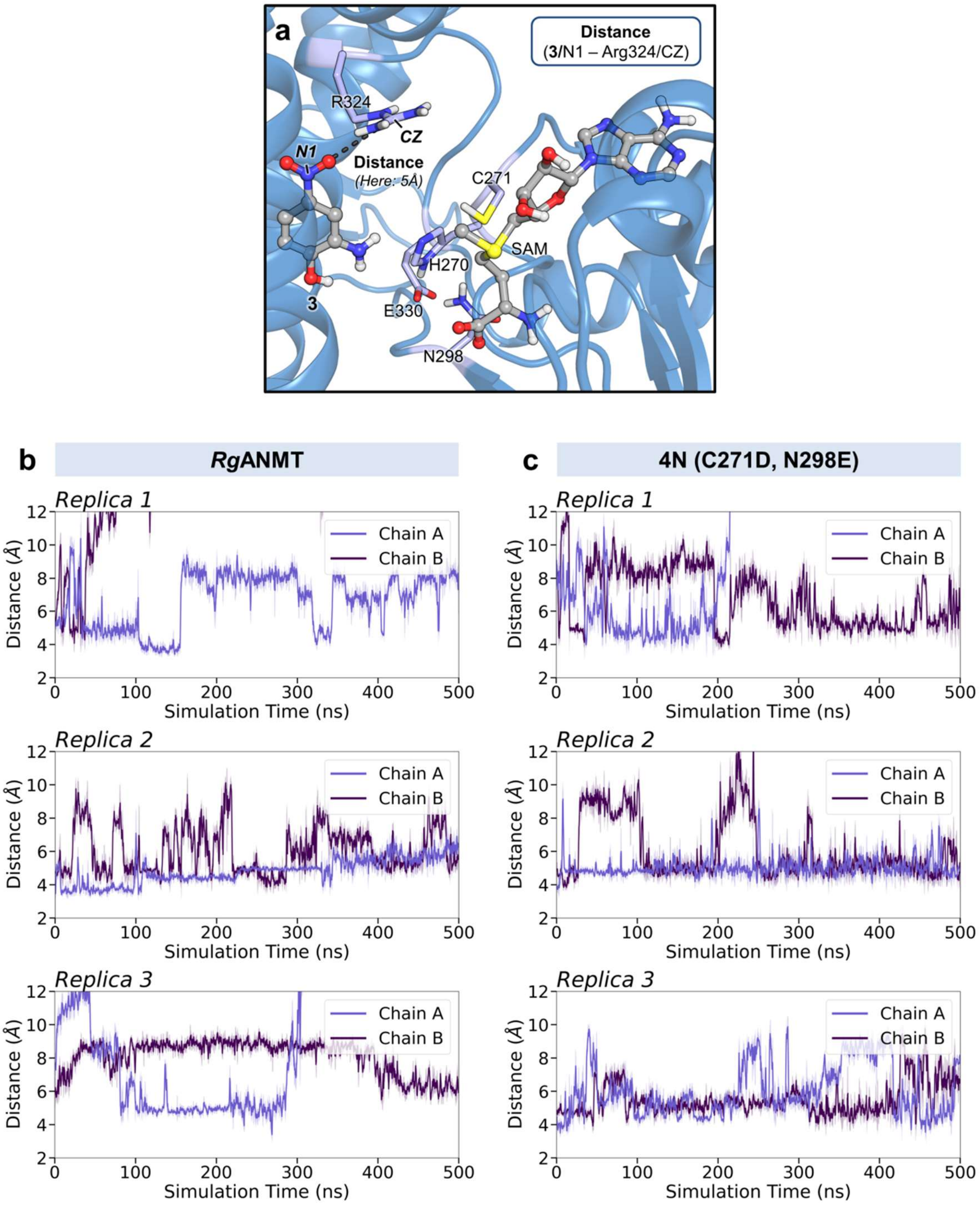

Figure S25 – Interaction between substrate **3**'s nitro group and Arg324 residue along the MD simulations with *Rg*ANMT and 4N. a: Molecular representation of the measured distance, which is between the nitrogen atom from 3's nitro group (N1) and the central carbon atom from Arg324's guanidinium group (CZ). The structural reference depicted corresponds to a frame extracted from the MD of *Rg*ANMT with **3**, featuring a representative (3/N1-Arg324/CZ) distance of 5 Å. Apart from the substrate and Arg324, active site residues and SAM are shown. b: Time evolution of the (3/N1-Arg324/CZ) distance in three MD replicas of 500 ns for wildtype *Rg*ANMT with substrate 3. c: Time evolution of the (3/N1-Arg324/CZ) distance in three MD replicas of 500 ns for variant 4N with substrate **3**.

Open and closed conformational analysis

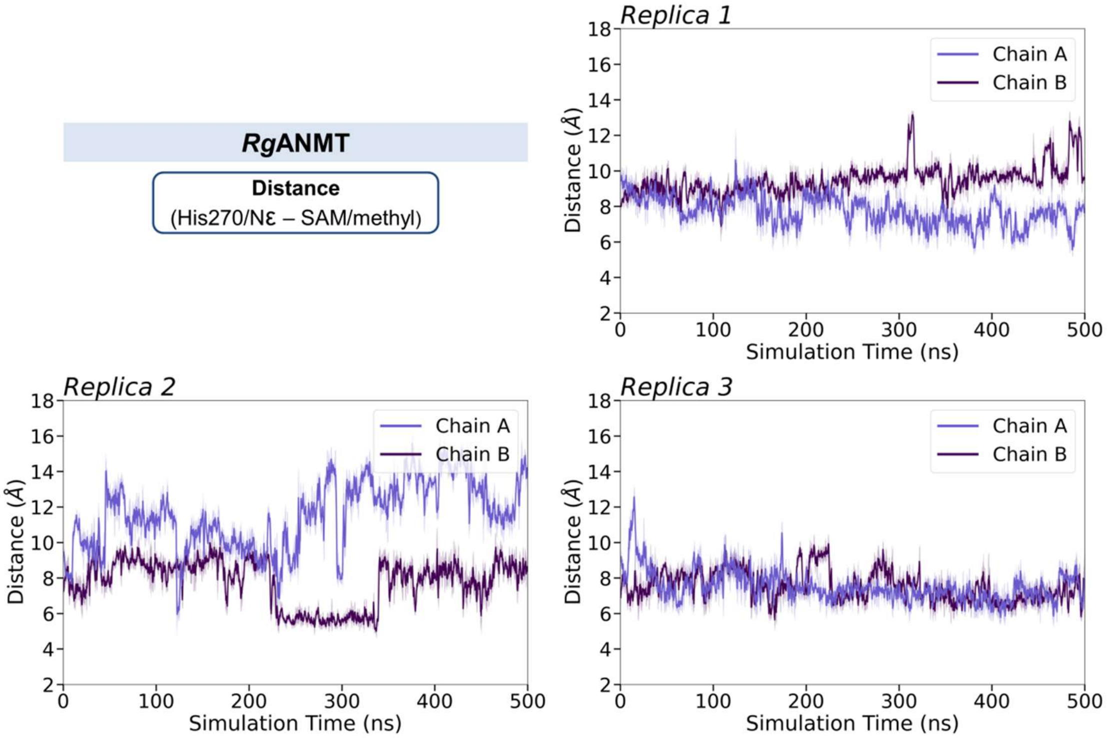

Figure S26 – Time evolution of the “catalytic histidine – reactive SAM methyl” distance in three replicas of 500 ns of MD simulations for the wildtype *Rg*ANMT holoenzyme. The distance has been measured between the epsilon nitrogen atom from His270 and the reactive carbon atom from SAM.

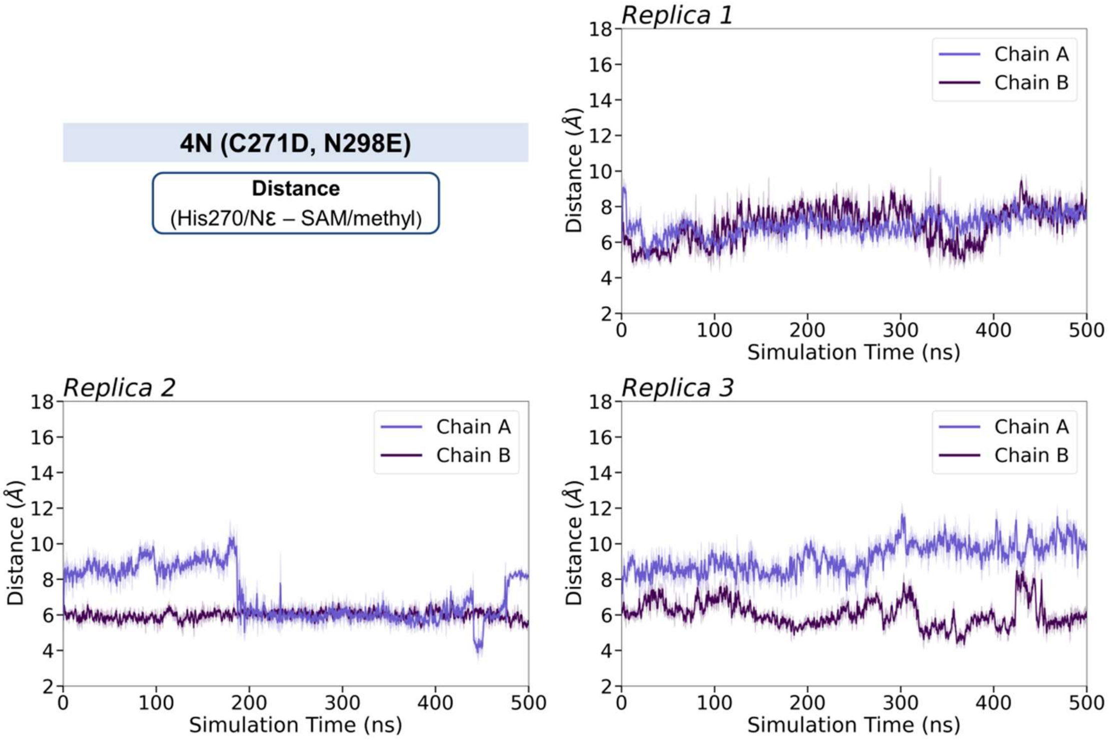

Figure S27 – Time evolution of the “catalytic histidine – reactive SAM methyl” distance in three replicas of 500 ns MD simulations for the variant 4N holoenzyme. The distance has been measured between the epsilon nitrogen atom from His270 and the reactive carbon atom from SAM.

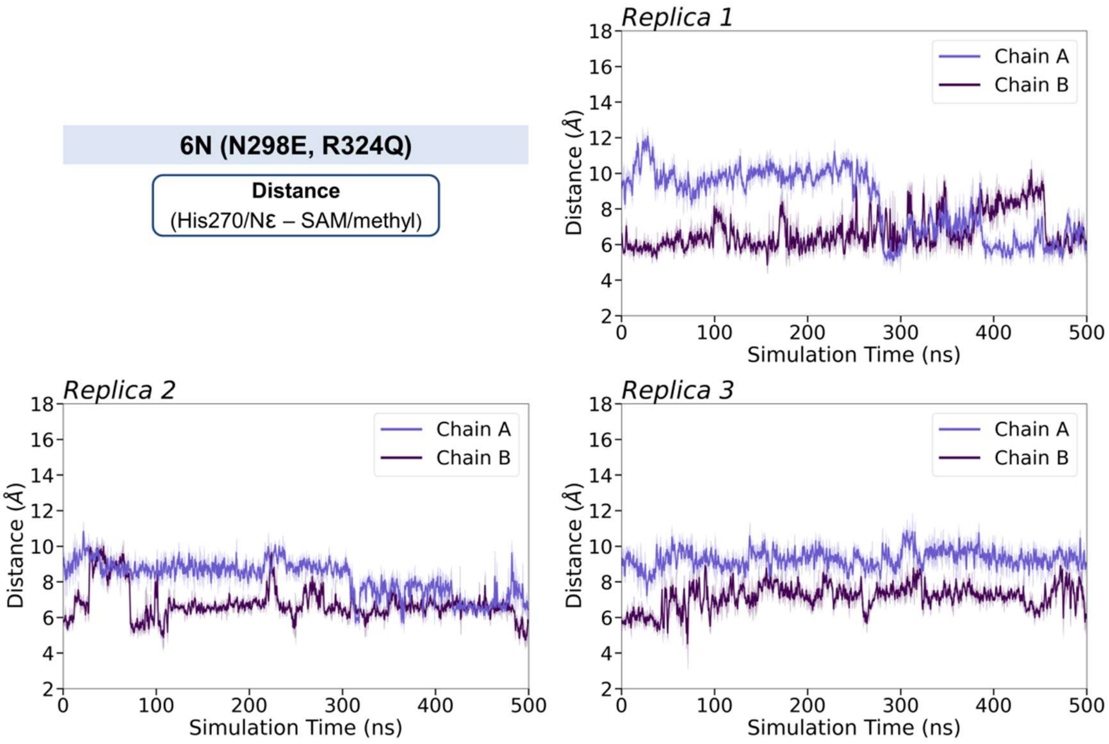

Figure S28 – Time evolution of the "catalytic histidine – reactive SAM methyl" distance in three replicas of 500 ns of MD simulations for the variant 6N holoenzyme. The distance has been measured between the epsilon nitrogen atom from His270 and the reactive carbon atom from SAM.

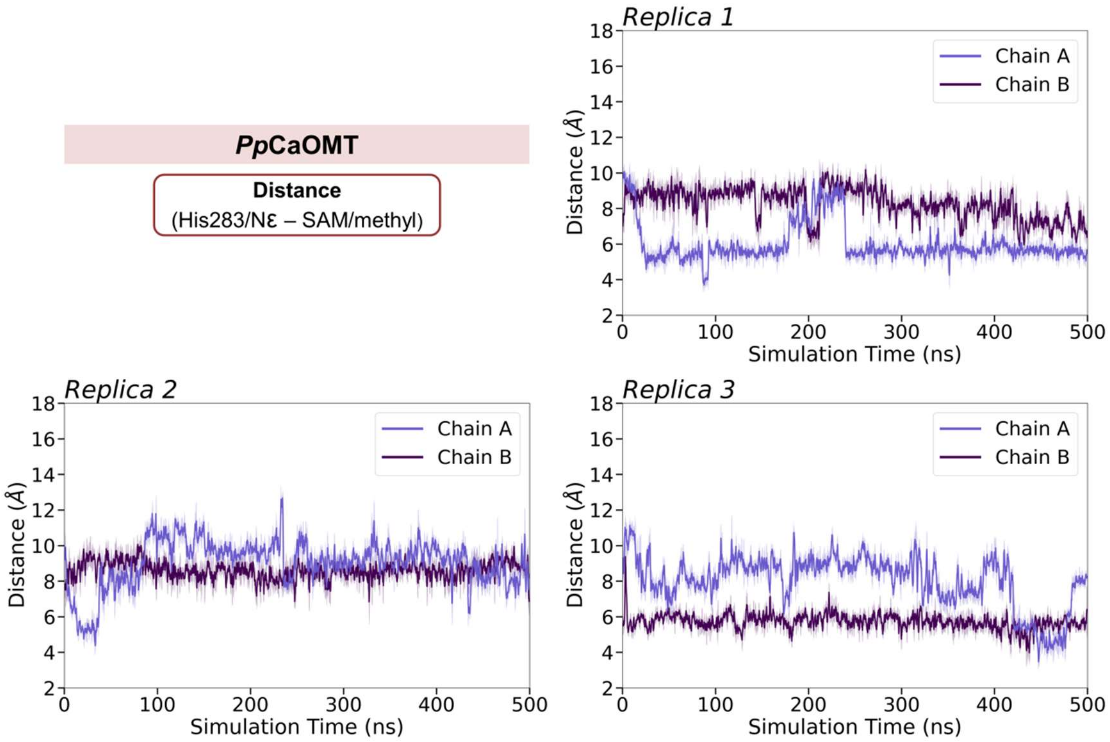

Figure S29 – Time evolution of the "catalytic histidine – reactive SAM methyl" distance in three replicas of 500 ns of MD simulations for the wildtype *Pp*CaOMT holoenzyme. The distance has been measured between the epsilon nitrogen atom from His283 and the reactive carbon atom from SAM.

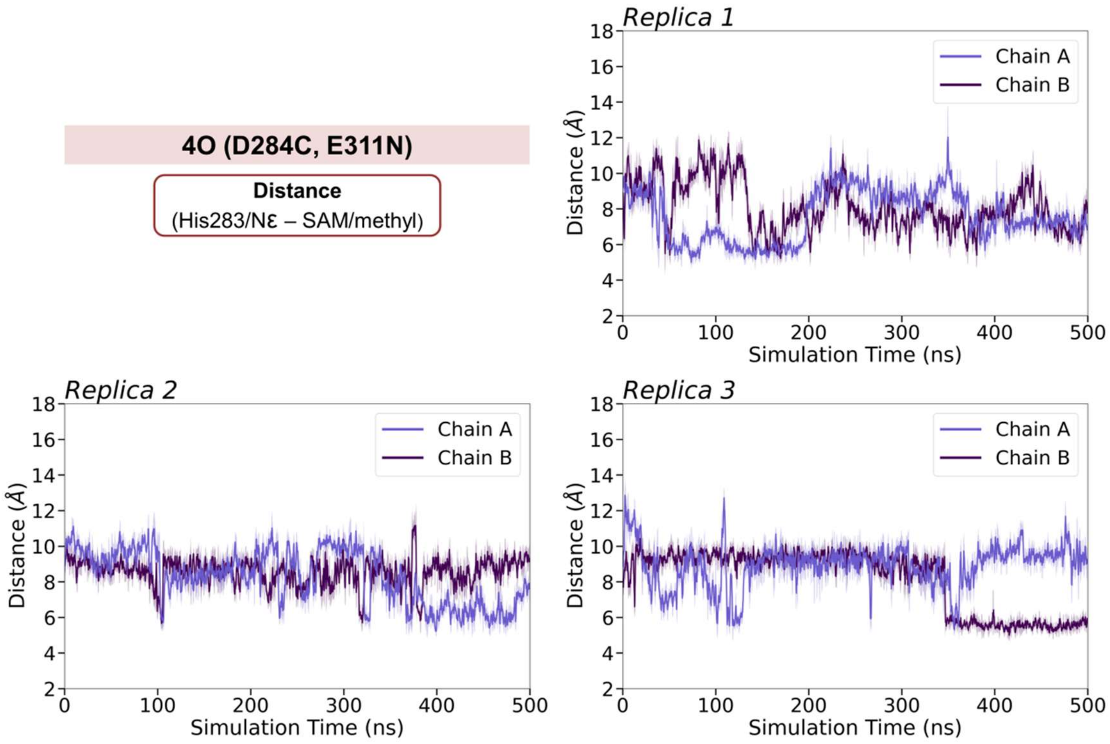

Figure S30 – Time evolution of the "catalytic histidine – reactive SAM methyl" distance in three replicas of 500 ns of MD simulations for the variant 4O holoenzyme. The distance has been measured between the epsilon nitrogen atom from His283 and the reactive carbon atom from SAM.

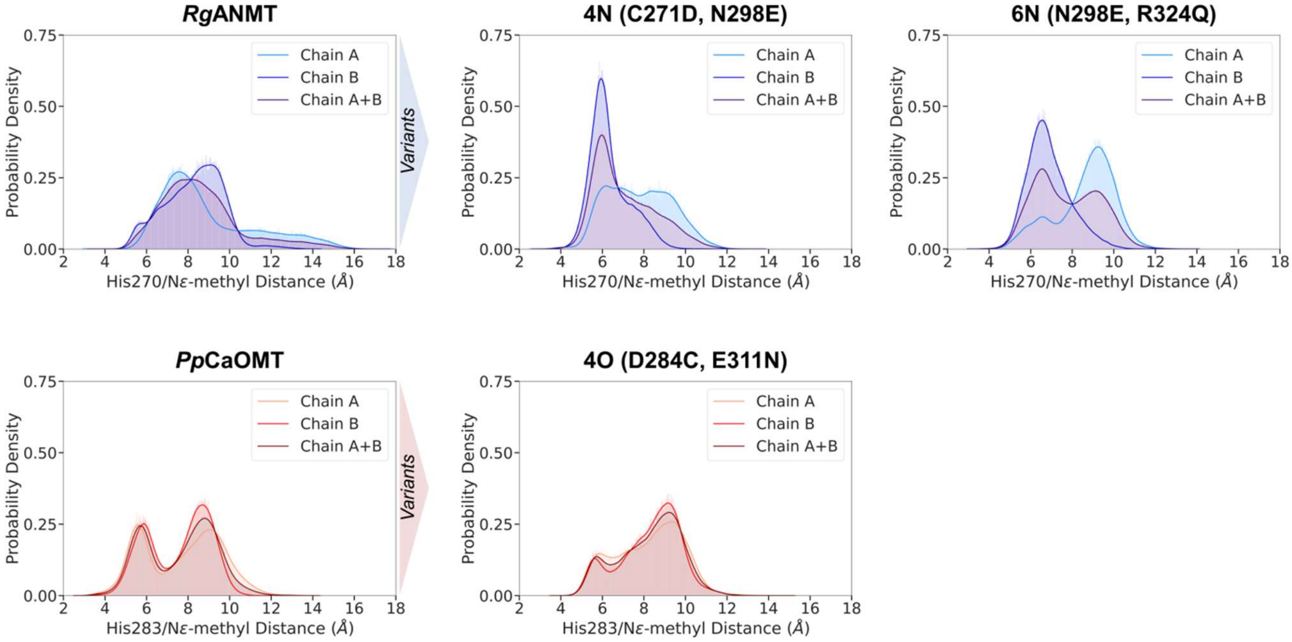

Figure S31 – Kernel density estimation (KDE) analysis of the "catalytic histidine - reactive SAM methyl" distance obtained from the collective analysis of MD trajectories of the following holoenzyme systems: the wildtype *Rg*ANMT and *Pp*CaOMT, and their corresponding variants 4N and 6N, and 4O. The probability distribution has been calculated considering separately each chain, and the both of them (Chain A+B). Taking the *Pp*CaOMT probability density results as a reference, we have qualitatively associated the term "closed conformation" to the structures with His-methyl distances below 6 Å, and "open conformation" to His-methyl distances over 8 Å.

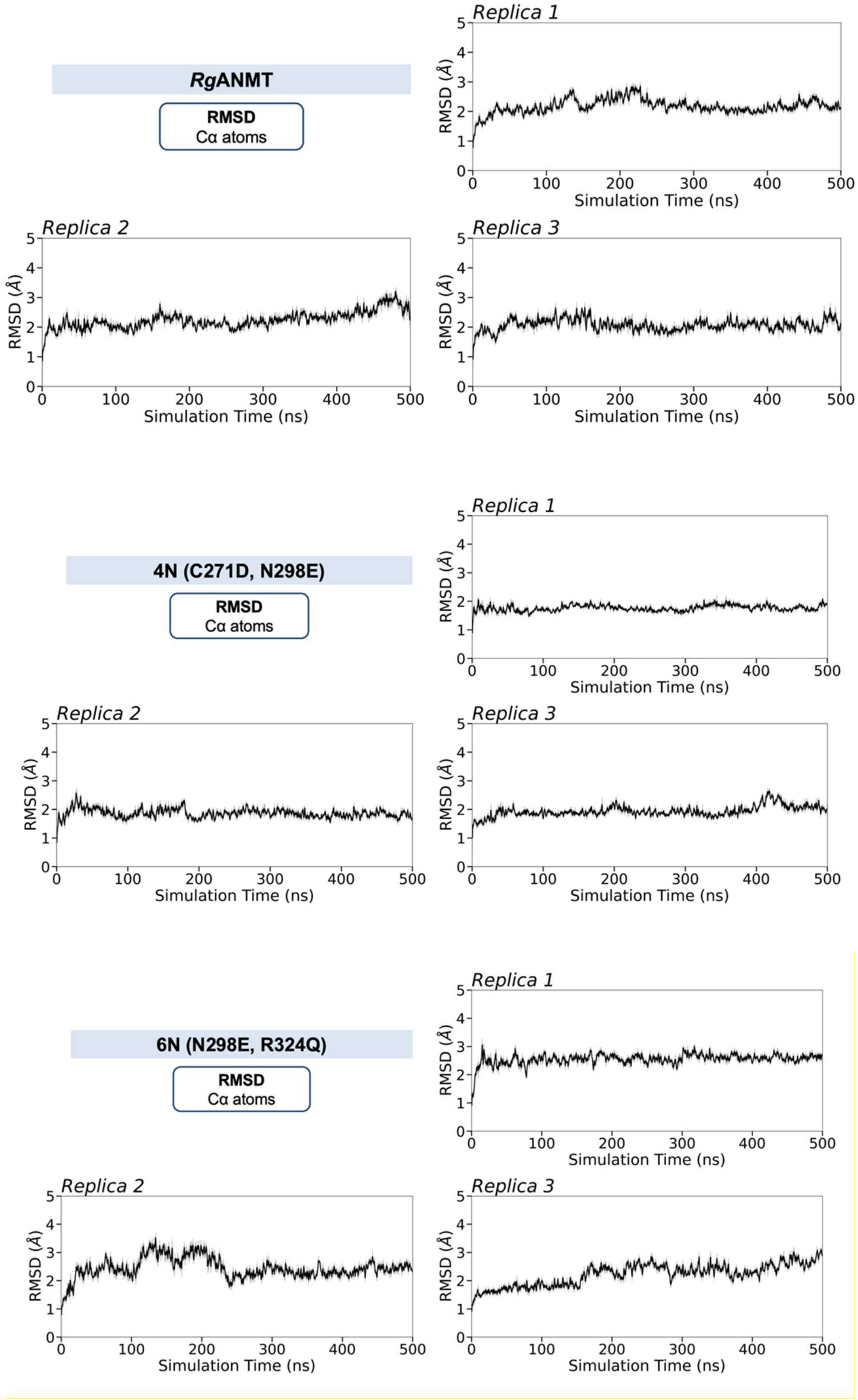

Figure S32 – Time evolution of the root-mean-square deviation (RMSD) in Å for the three replicas of 500 ns of MD simulations for *Rg*ANMT, 4N and 6N systems.

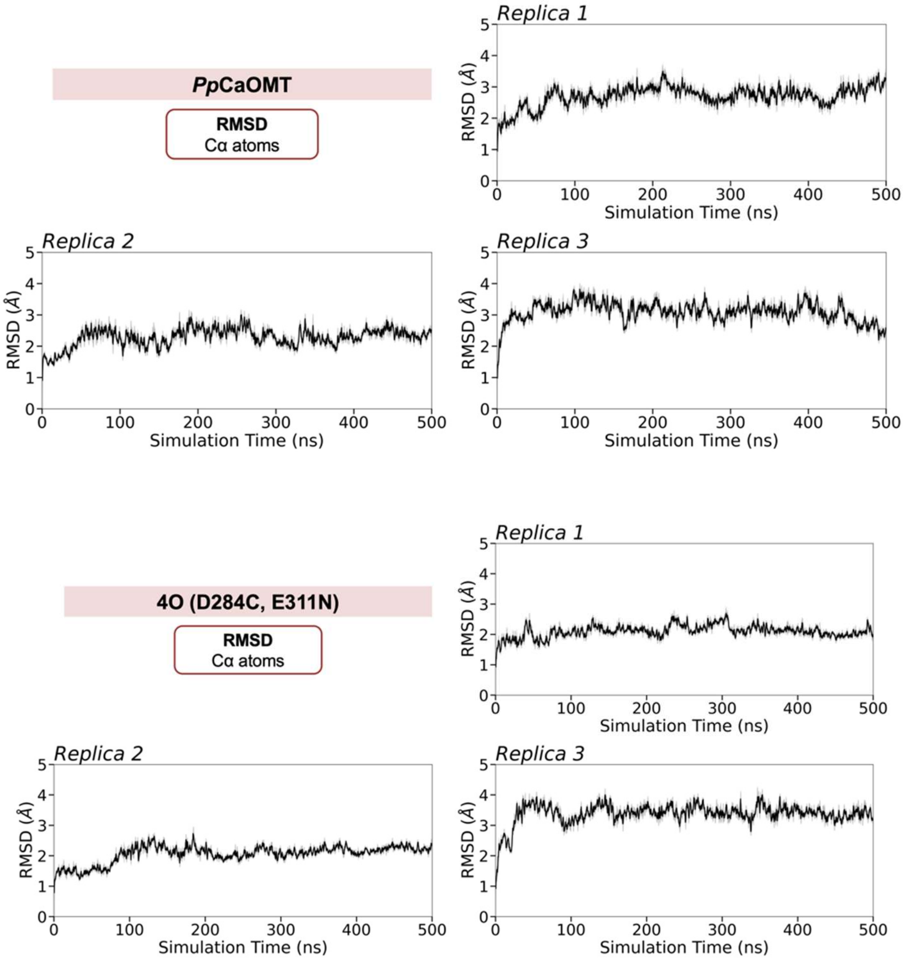

Figure S33 – Time evolution of the root-mean-square deviation (RMSD) in Å for the three replicas of 500 ns of MD simulations for *Pp*CaOMT and 4O systems.

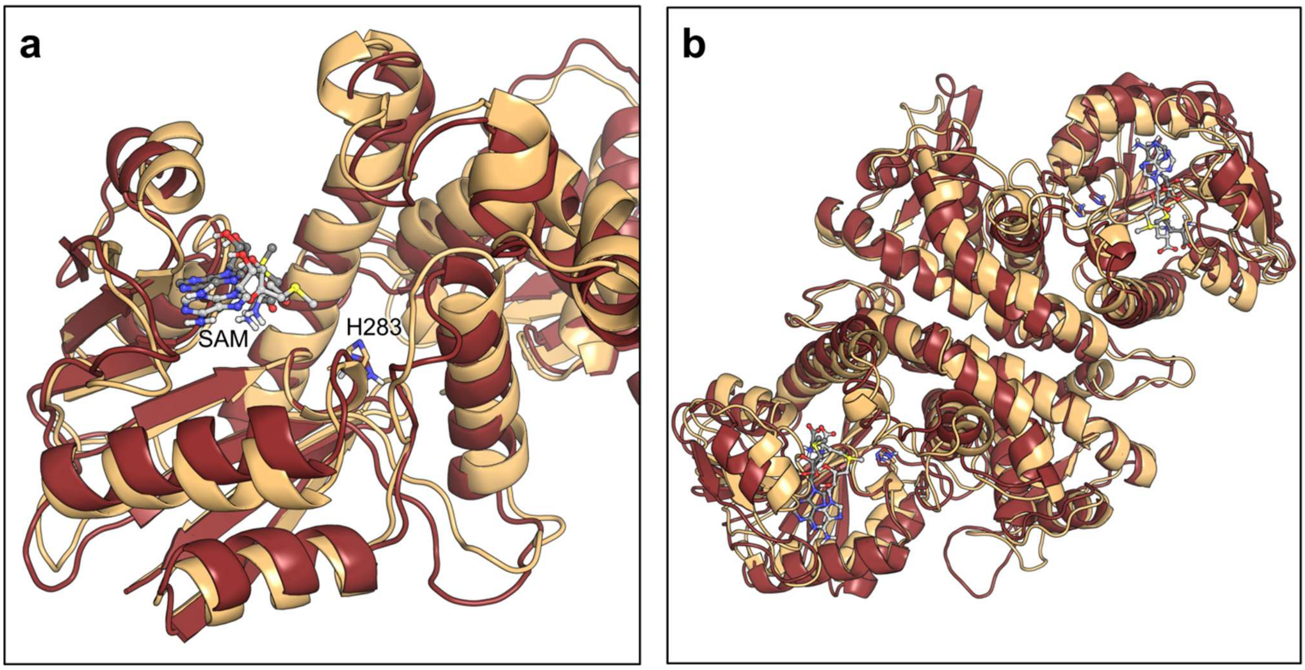

Figure S34 – Structural overlay of two representative frames from the MD trajectories of the *Pp*CaOMT holoenzyme model. a: Comparison of the open and closed conformational states. In the open, the protein is depicted in dark red colour and SAM in dark grey; in the closed, the protein is depicted in orange colour and SAM in light grey. The catalytic histidine is explicitly shown with stick representation. b: Extended view encompassing the whole homodimer. The monomer from (a) displaying the open/closed differences is located at the bottom left side of the image.

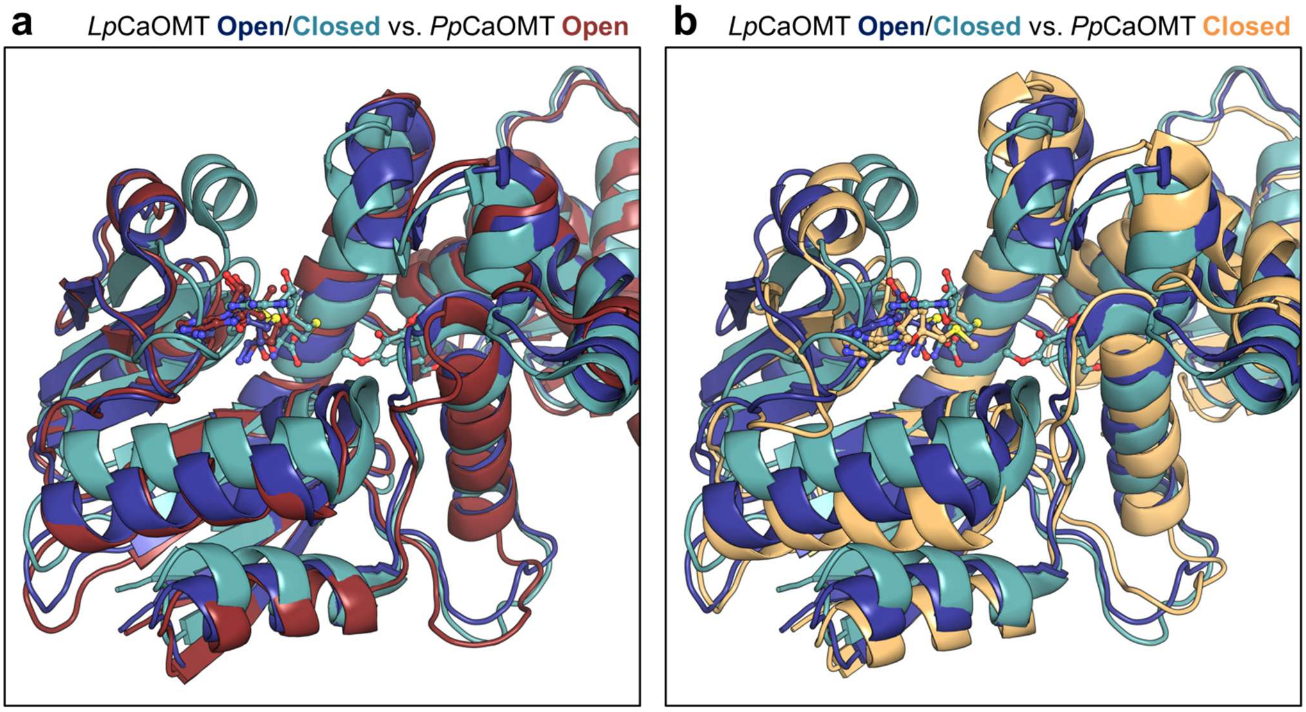

Figure S35 – Structural comparison of the open and closed crystallographic structures from *Lp*CaOMT (pdb: 3P9C and 3P9I) with the observed open and closed characters in the MD *Pp*CaOMT holoenzyme model. The open *Lp*CaOMT (holoenzyme) conformation is depicted in blue colour; the closed *Lp*CaOMT (product-bound) is depicted in teal; the open *Pp*CaOMT is depicted in dark red; and the closed *Pp*CaOMT is depicted in orange. The cofactors and the sinapaldehyde product (from closed *Lp*CaOMT) are explicitly shown in ball and stick representation. All structures have been aligned to the *Lp*CaOMT open conformation. a: Overlay of the *Lp*CaOMT open and closed conformation with the *Pp*CaOMT open conformation. b: Overlay of the *Lp*CaOMT open and closed conformation with the *Pp*CaOMT closed conformation.

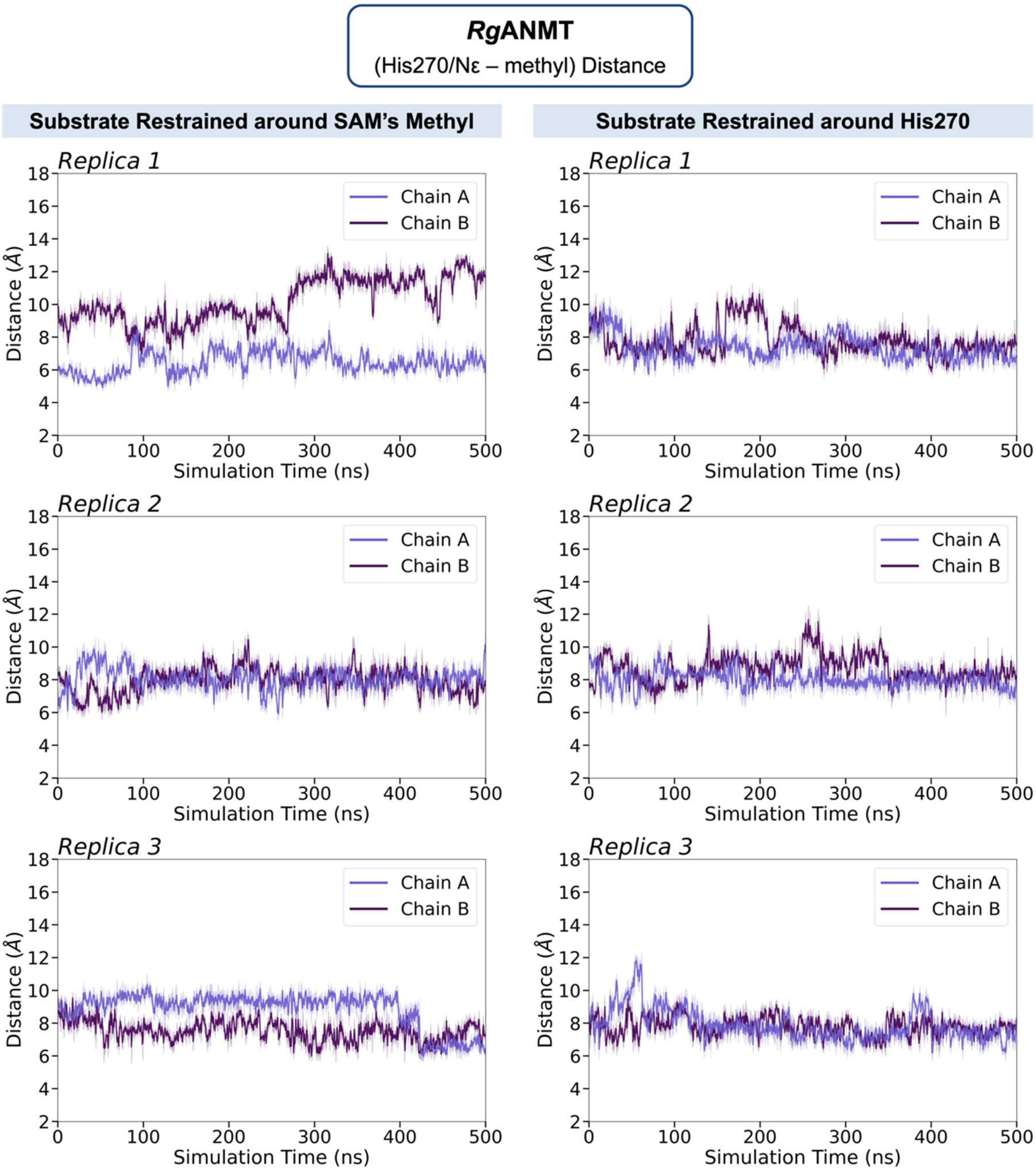

Figure S36 – Time evolution of the "catalytic histidine – reactive SAM methyl" distance in three replicas of 500 ns of restrained MD simulations for the substrate **3** bound wildtype *Rg*ANMT. The distance has been measured between the epsilon nitrogen atom from His270 and the reactive carbon atom from SAM. As described in Computational Methods, the restrained MD simulations are performed by applying a distance restraint between the substrate and SAM (left) and substrate and catalytic histidine (right).

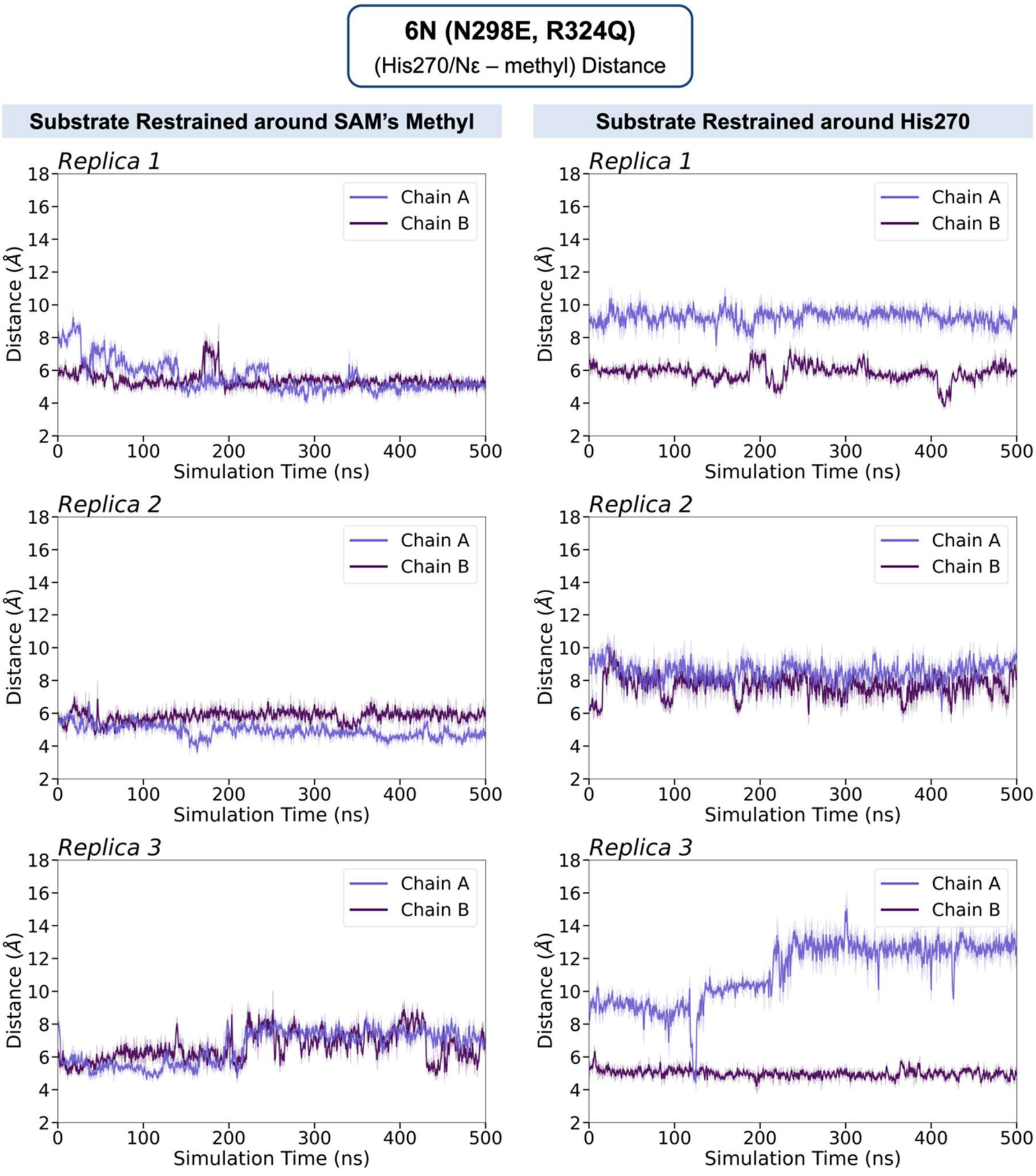

Figure S37 – Time evolution of the "catalytic histidine – reactive SAM methyl" distance in three replicas of 500 ns of restrained MD simulations for the substrate **3** bound wildtype 6N. The distance has been measured between the epsilon nitrogen atom from His283 and the reactive carbon atom from SAM. As described in Computational Methods, the restrained MD simulations are performed by applying a distance restraint between the substrate and SAM (left) and substrate and catalytic histidine (right).

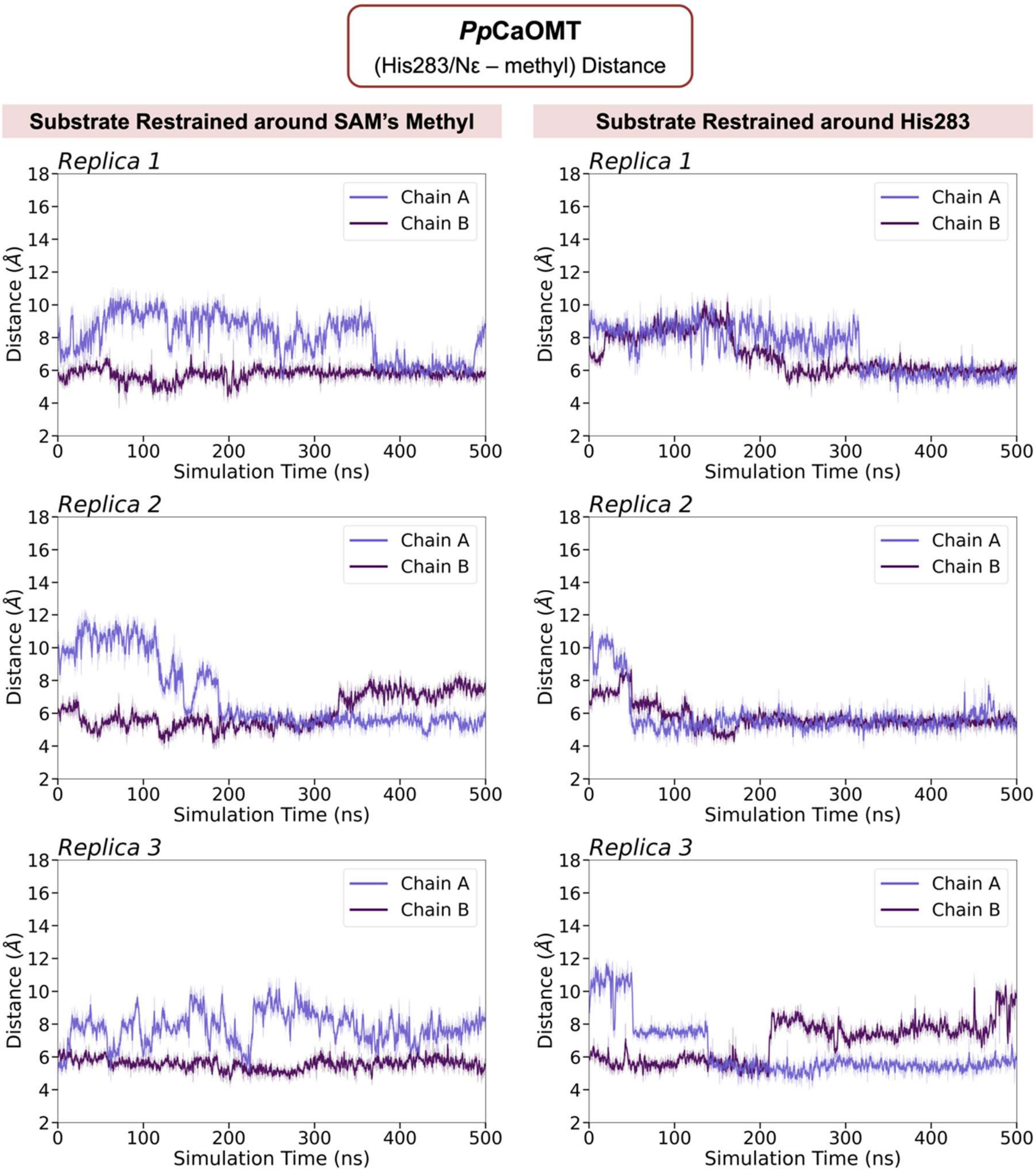

Figure S38 – Time evolution of the "catalytic histidine – reactive SAM methyl" distance in three replicas of 500 ns of restrained MD simulations for the substrate **3** bound wildtype *Pp*CaOMT. The distance has been measured between the epsilon nitrogen atom from His283 and the reactive carbon atom from SAM. As described in Computational Methods, the restrained MD simulations are performed by applying a distance restraint between the substrate and SAM (left) and substrate and catalytic histidine (right).

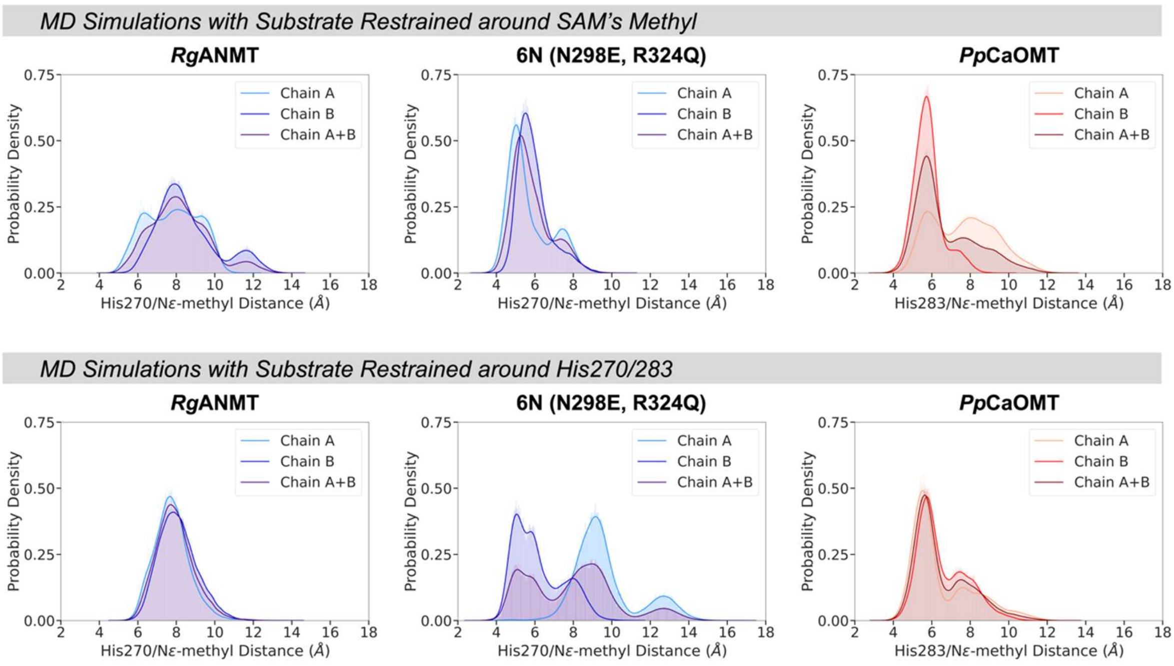

Figure S39 – Kernel density estimation (KDE) analysis of the "catalytic histidine - reactive SAM methyl" distance obtained from the collective analysis of restrained MD trajectories of the following ternary complex systems: the wildtype *Rg*ANMT and *Pp*CaOMT, and the variant 6N. The probability distribution has been calculated considering separately each chain, and the both of them (Chain A+B). Taking the *Pp*CaOMT probability density results as a reference, we have qualitatively associated the term "closed conformation" to the structures with His-methyl distances below 6 Å, and "open conformation" to His-methyl distances over 8 Å. As described in Computational Methods, the restrained MD simulations are performed by applying a distance restraint between the substrate and SAM (top) and substrate and catalytic histidine (below).

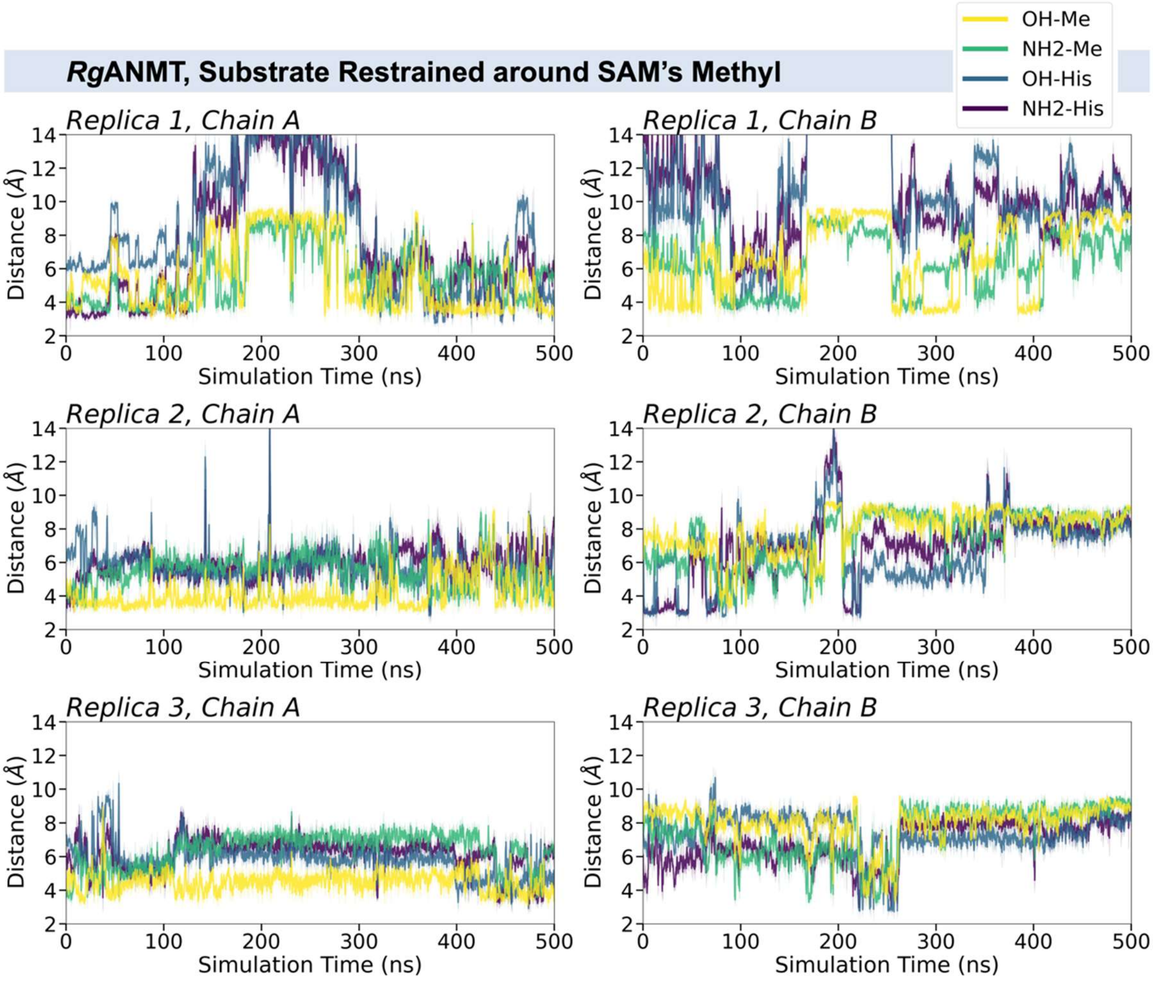

Figure S40 – Time evolution of the substrate **3 (OH/NH$_2$)**-methyl/histidine distances in three replicas of 500 ns of restrained MD simulations for the substrate **3** bound wildtype *Rg*ANMT with a distance restraint between the SAM and the substrate. The distances has been measured between: 1) the epsilon nitrogen atom from His270 and the amino nitrogen of the substrate (purple); 2) the epsilon nitrogen atom from His270 and the hydroxyl oxygen of the substrate (blue); 3) the reactive carbon atom from SAM and the amino nitrogen of the substrate (green); and 4) the reactive carbon atom from SAM and the hydroxyl oxygen of the substrate (yellow).

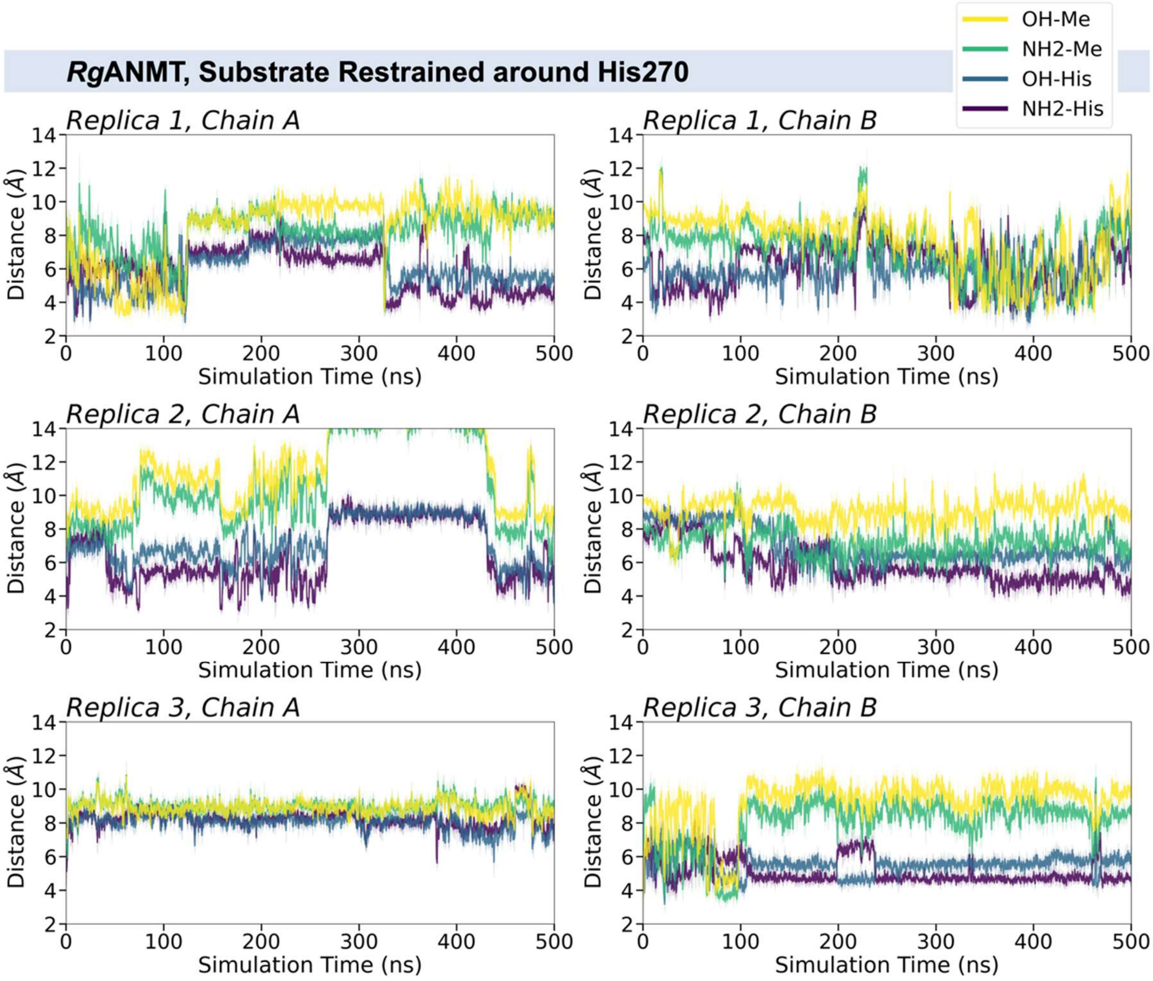

Figure S41 – Time evolution of the substrate **3 (OH/NH$_2$)**-methyl/histidine distances in three replicas of 500 ns of restrained MD simulations for the substrate **3** bound wildtype *Rg*ANMT with a distance restraint between the His270 and the substrate. The distances have been measured between: 1) the epsilon nitrogen atom from His270 and the amino nitrogen of the substrate (purple); 2) the epsilon nitrogen atom from His270 and the hydroxyl oxygen of the substrate (blue); 3) the reactive carbon atom from SAM and the amino nitrogen of the substrate (green); and 4) the reactive carbon atom from SAM and the hydroxyl oxygen of the substrate (yellow).

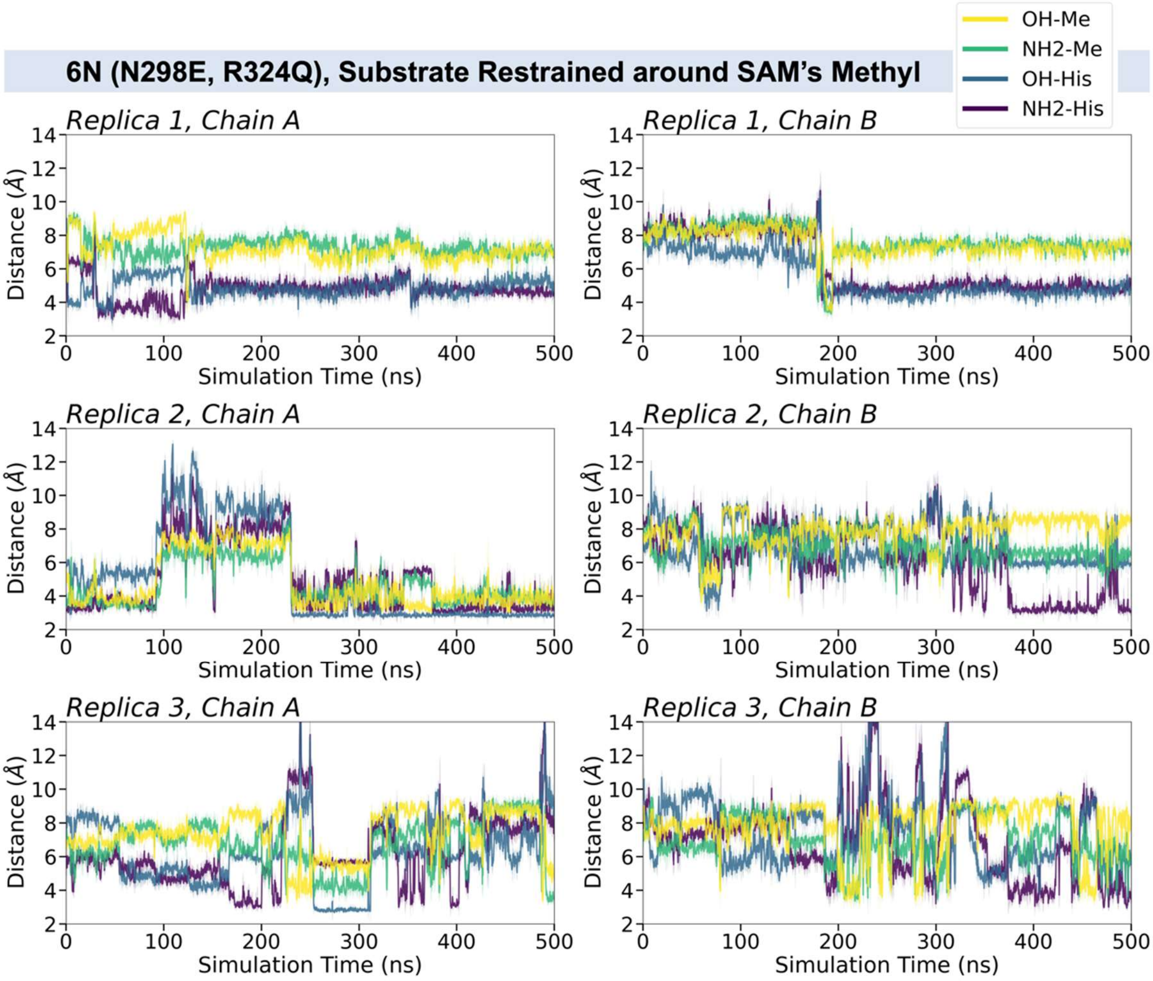

Figure S42 – Time evolution of the substrate **3 (OH/NH$_2$)**-methyl/histidine distances in three replicas of 500 ns of restrained MD simulations for the substrate **3** bound 6N variant with a distance restraint between the SAM and the substrate. The distances have been measured between: 1) the epsilon nitrogen atom from His270 and the amino nitrogen of the substrate (purple); 2) the epsilon nitrogen atom from His270 and the hydroxyl oxygen of the substrate (blue); 3) the reactive carbon atom from SAM and the amino nitrogen of the substrate (green); and 4) the reactive carbon atom from SAM and the hydroxyl oxygen of the substrate (yellow).

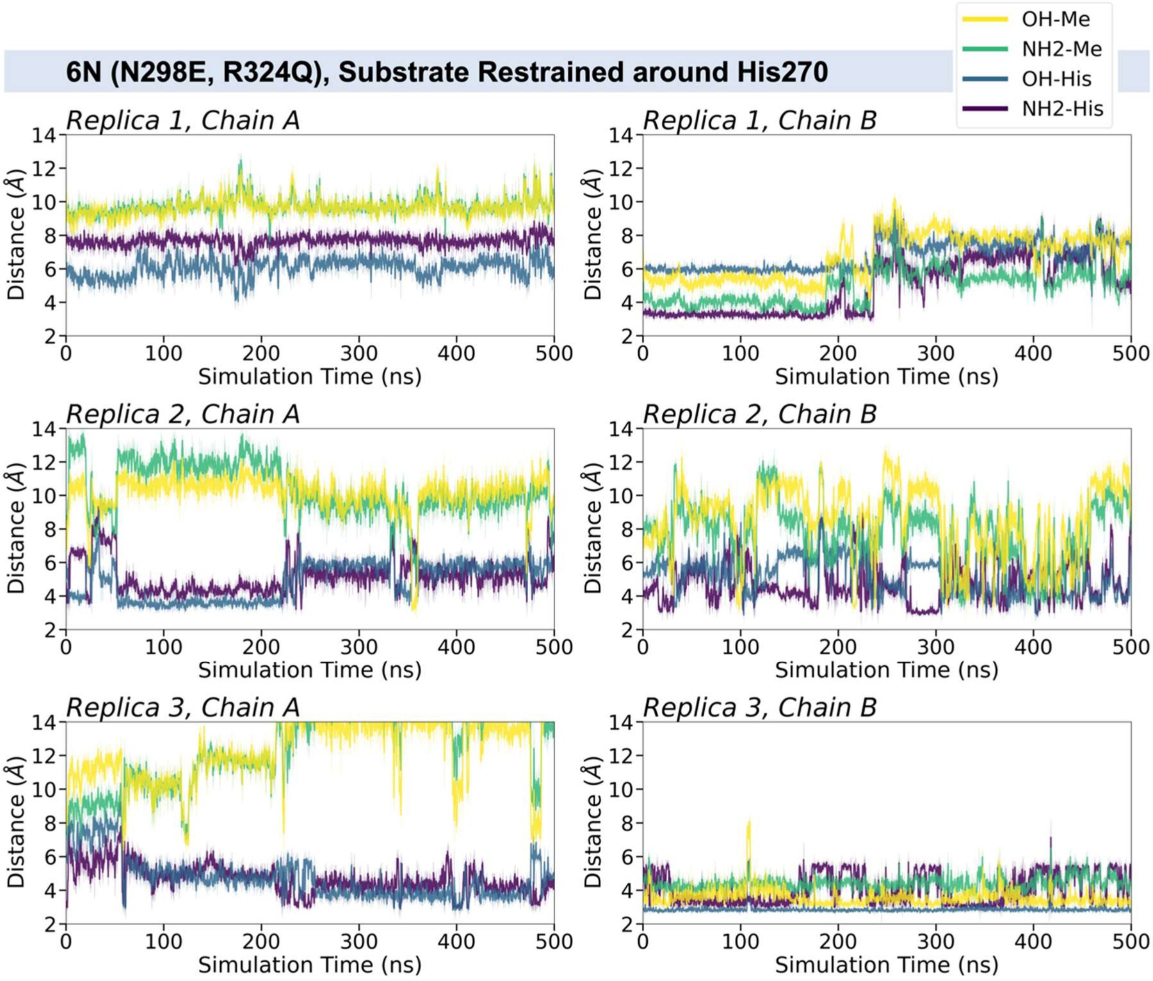

Figure S43 – Time evolution of the substrate **3 (OH/NH$_2$)**-methyl/histidine distances in three replicas of 500 ns of restrained MD simulations for the substrate **3** bound 6N variant with a distance restraint between catalytic histidine and the substrate. The distances have been measured between: 1) the epsilon nitrogen atom from His270 and the amino nitrogen of the substrate (purple); 2) the epsilon nitrogen atom from His270 and the hydroxyl oxygen of the substrate (blue); 3) the reactive carbon atom from SAM and the amino nitrogen of the substrate (green); and 4) the reactive carbon atom from SAM and the hydroxyl oxygen of the substrate (yellow).

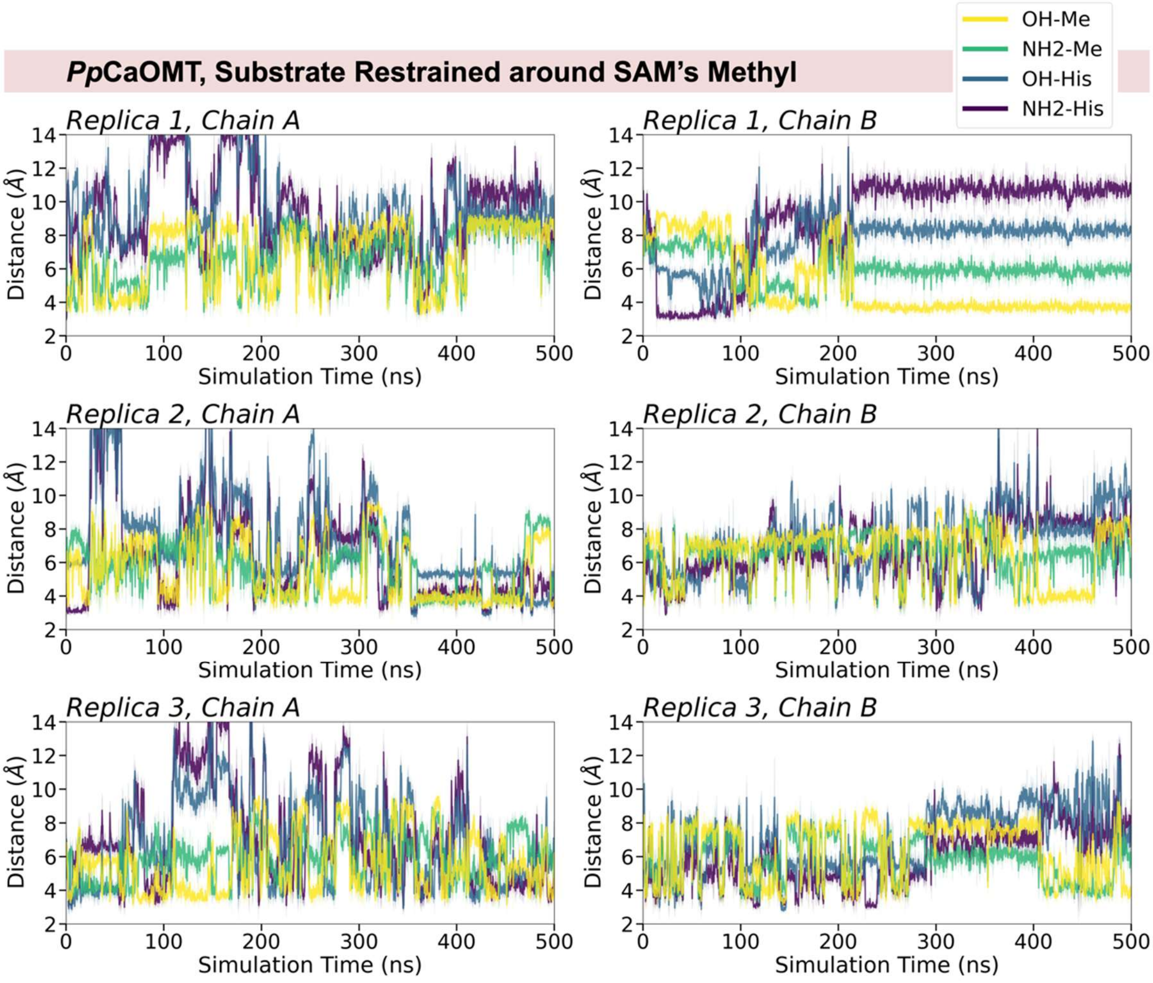

Figure S44 – Time evolution of the substrate **3 (OH/NH₂)**-methyl/histidine distances in three replicas of 500 ns of restrained MD simulations for the substrate **3** bound wildtype *Pp*CaOMT with a distance restraint between the SAM and the substrate. The distances have been measured between: 1) the epsilon nitrogen atom from His283 and the amino nitrogen of the substrate (purple); 2) the epsilon nitrogen atom from His283 and the hydroxyl oxygen of the substrate (blue); 3) the reactive carbon atom from SAM and the amino nitrogen of the substrate (green); and 4) the reactive carbon atom from SAM and the hydroxyl oxygen of the substrate (yellow).

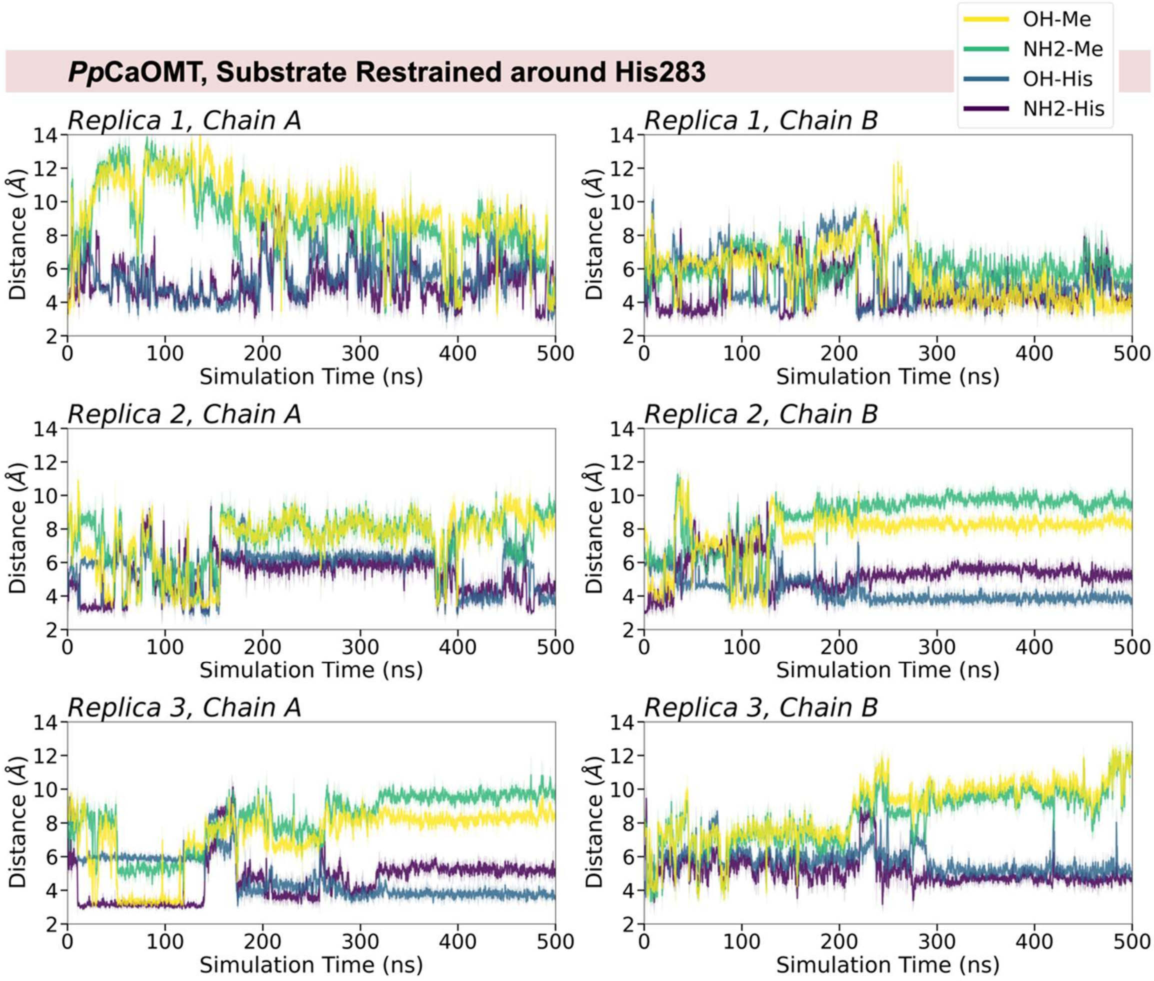

Figure S45 – Time evolution of the substrate **3 (OH/NH$_2$)**-methyl/histidine distances in three replicas of 500 ns of restrained MD simulations for the substrate **3** bound wildtype *Pp*CaOMT with a distance restraint between the catalytic histidine and the substrate. The distances have been measured between: 1) the epsilon nitrogen atom from His283 and the amino nitrogen of the substrate (purple); 2) the epsilon nitrogen atom from His283 and the hydroxyl oxygen of the substrate (blue); 3) the reactive carbon atom from SAM and the amino nitrogen of the substrate (green); and 4) the reactive carbon atom from SAM and the hydroxyl oxygen of the substrate (yellow).

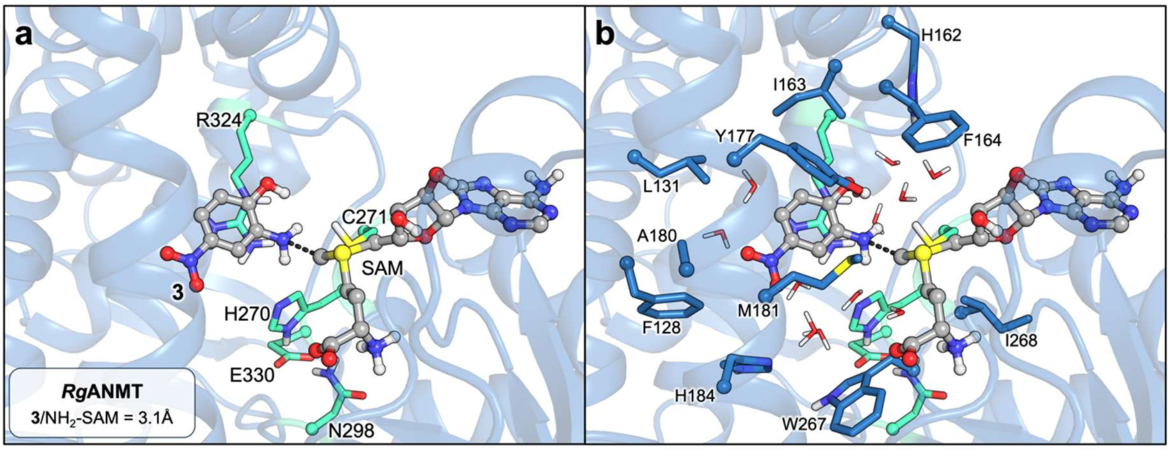

Figure S46 – Representative structure that depicts the interactions between substrate 3 and active site residues of *Rg*ANMT obtained from restrained MD simulations. a: Molecular representation of the substrate **3** (in grey), catalytic residues (in green) and SAM (in grey). The substrate is properly position for methylation in N. b: Same structure including active site residues (in blue) and active site water molecules indicating a higher degree of solvent exposure when the substrate is bound.

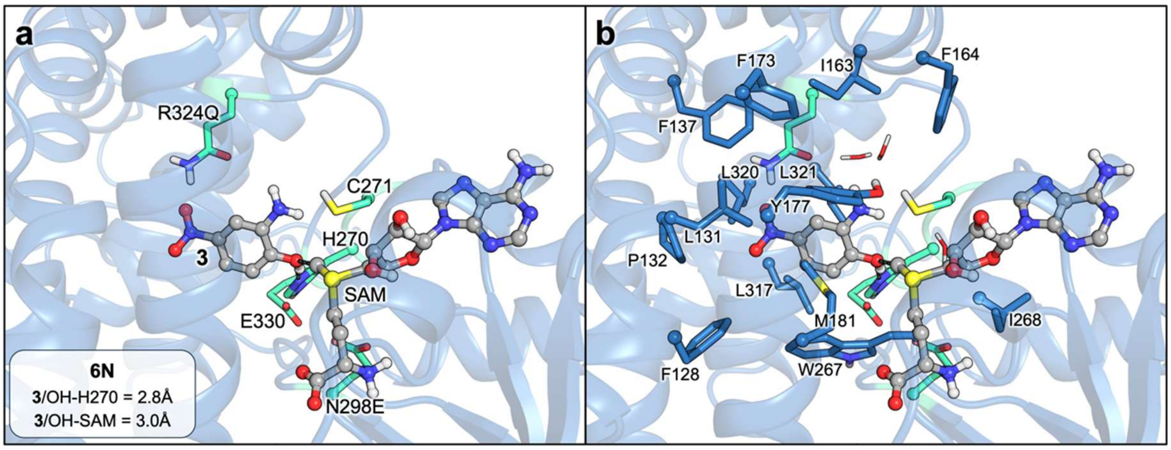

Figure S47 – Representative structure that depicts the interactions between substrate 3 and active site residues of 6N variant obtained from restrained MD simulations. a: Molecular representation of the substrate **3** (in grey), catalytic residues (in green) and SAM (in grey). The substrate is properly positioned for methylation in O. b: Same structure including active site residues (in blue) and active site water molecules indicating a significant confinement of the active site in the closed conformation.

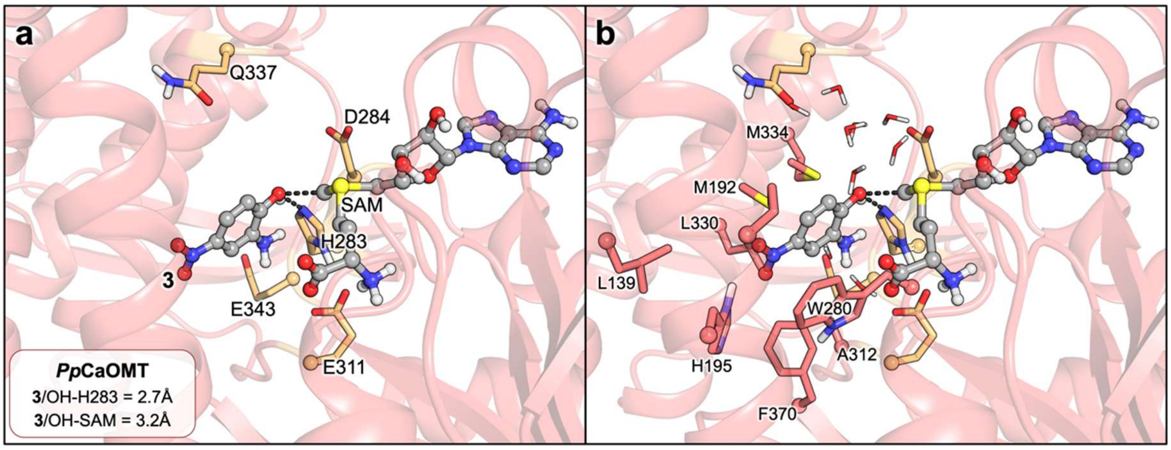

Figure S48 – Representative structure that depicts the interactions between substrate 3 and active site residues of *Pp*CaOMT variant obtained from restrained MD simulations. a: Molecular representation of the substrate **3** (in grey), catalytic residues (in orange) and SAM (in grey). The substrate is properly positioned for methylation in O. b: Same structure including active site residues (in pink) and active site water molecules indicating a significant confinement of the active site in the closed conformation.

Principal component analysis of MD trajectories

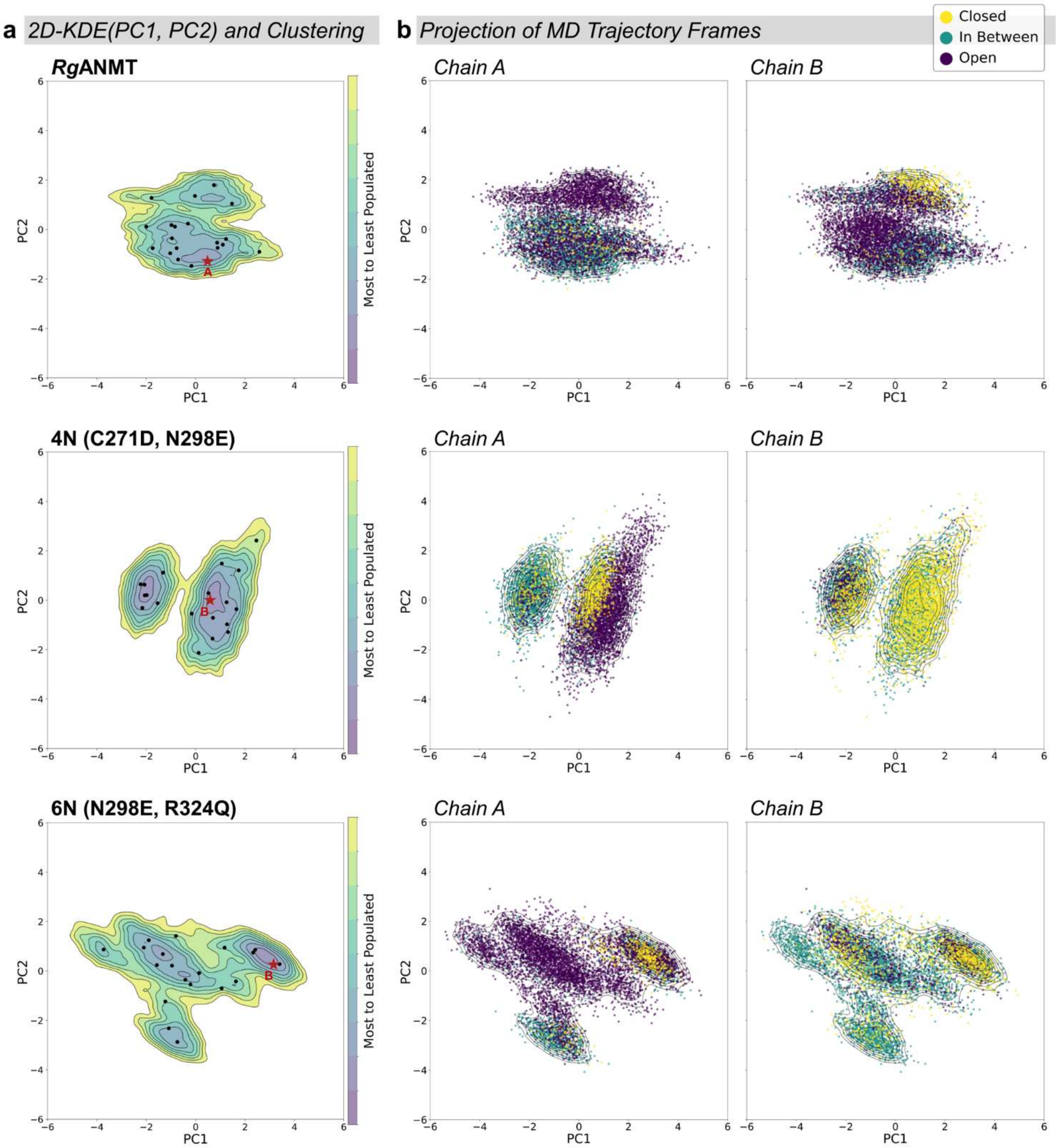

Figure S49 – Principal component analysis (PCA) results for the wildtype *Rg*ANMT holoenzyme, and its variants 4N and 6N. a: Probability distribution of the holoenzyme conformations along the two principal collective motions (PC1 and PC2) obtained from PCA of each system's MD trajectory data considering the coordinates of all alpha carbons. The 20 cluster centres retrieved from the k-means clustering analysis are scattered on the 2D probability density map with black dots and a red star. The red star marks the cluster to which the system's representative frame used for electrostatic analysis (see below) belongs, indicating the letter 'A' or 'B' for the specific chain. b: Colour-coded projection of the MD trajectory frames over the PC1 and PC2 collective coordinate space. Frames are coloured depending on the chain's open/closed state, which is defined by the "catalytic histidine – reactive SAM methyl" distance value: yellow indicates a closed state (≤ 6 Å), purple an open state (≥ 8 Å), and green the conformations with distances in between (6-8 Å). The obtained PCA results support that the active site mutations introduce a change in the *Rg*ANMT conformational dynamics.

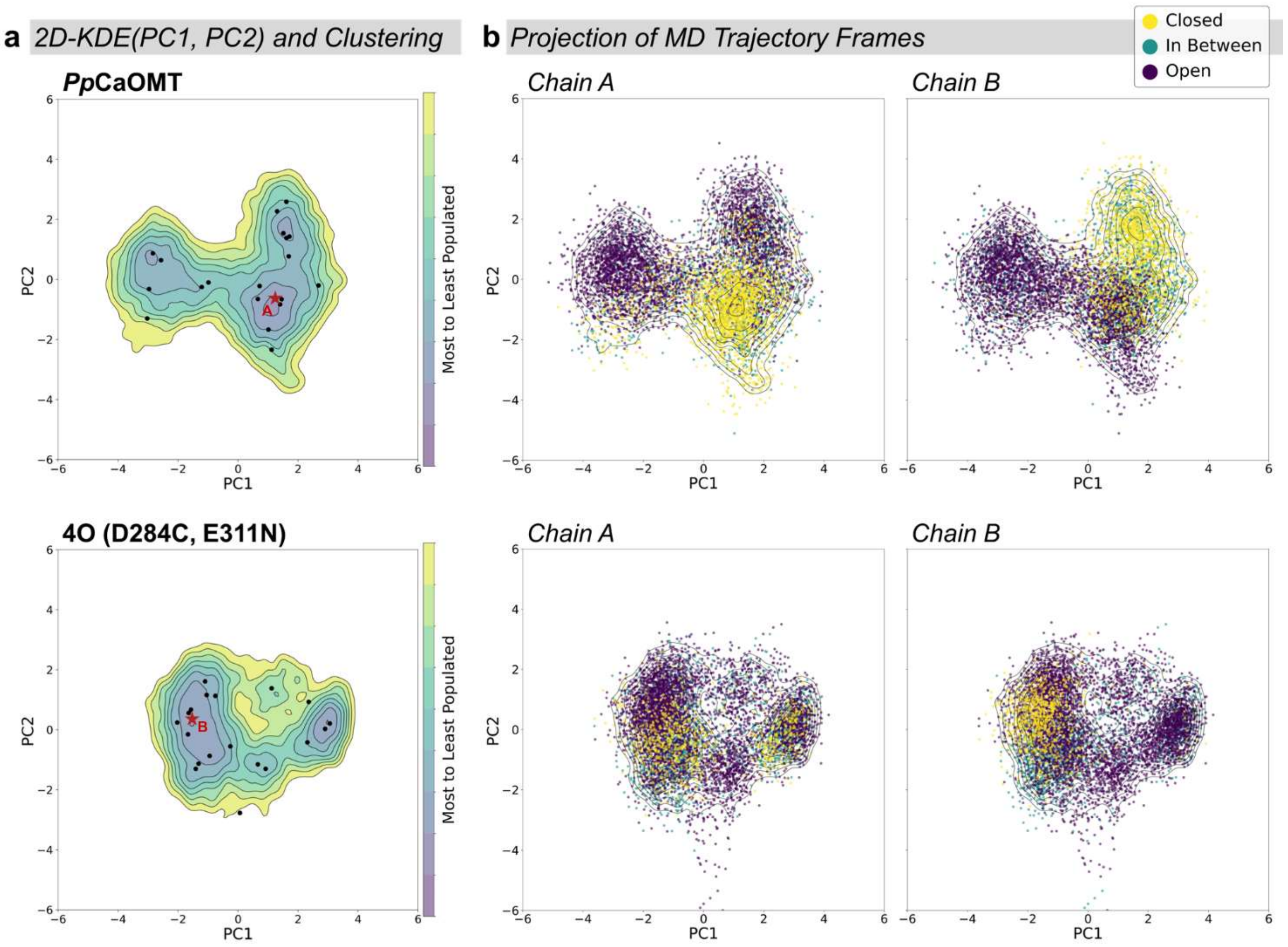

Figure S50 – Principal component analysis (PCA) results for the wildtype *Pp*CaOMT holoenzyme, and its variant 4O. a: Probability distribution of the holoenzyme conformations along the two principal collective motions (PC1 and PC2) obtained from PCA of each system's MD trajectory data considering the coordinates of all alpha carbons. The 20 cluster centres retrieved from the k-means clustering analysis are scattered on the 2D probability density map with black dots and a red star. The red star marks the cluster to which the system's representative frame used for electrostatic analysis (see below) belongs, indicating the letter 'A' or 'B' for the specific chain. b: Colour-coded projection of the MD trajectory frames over the PC1 and PC2 collective coordinate space. Frames are coloured depending on the chain's open/closed state, which is defined by the "catalytic histidine – reactive SAM methyl" distance value: yellow indicates a closed state (≤ 6 Å), purple an open state (≥ 8 Å), and green the conformations with distances in between (6-8 Å). Interestingly, the PC1 motion from *Pp*CaOMT unlocks the closed conformation; and the PC2 motion determines which of the two chains is closed, as the open-to-closed transition led by PC2 is an antiparallel one. The obtained PCA results support that the active site mutations introduce a change in the *Pp*CaOMT conformational dynamics.

Electrostatic analysis

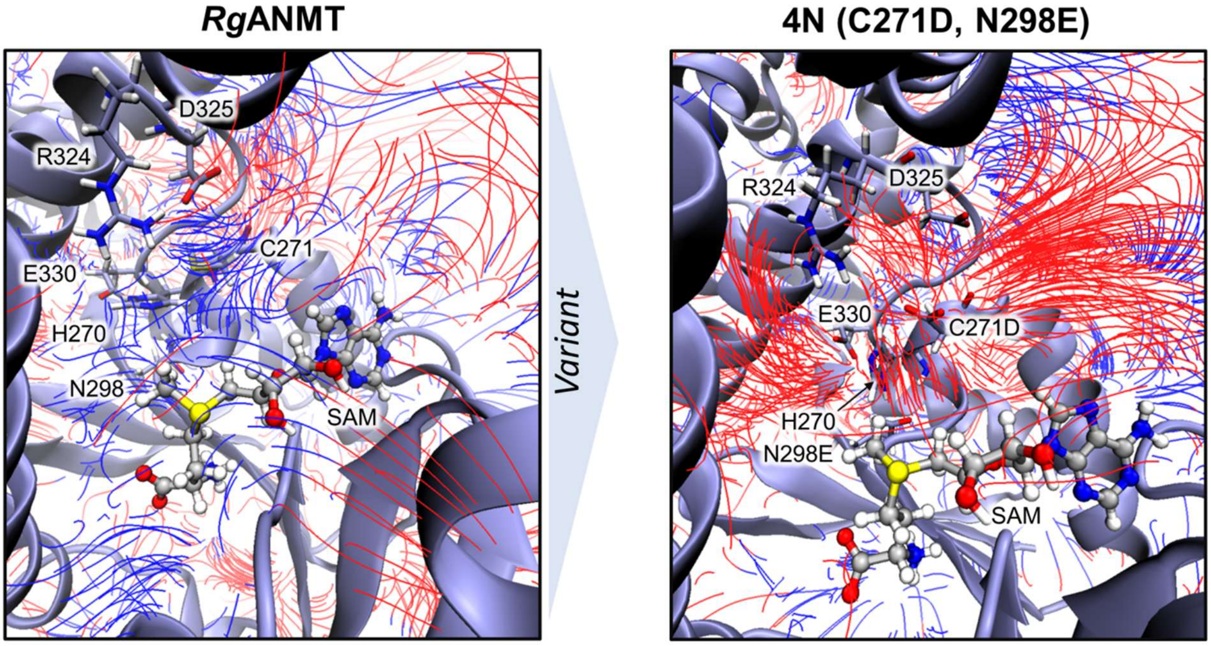

Figure S51 – Map of the electric field in the active site of *Rg*ANMT and variant 4N. The red-coloured line segments indicate negative electrostatic potentials, while blue segments represent positive electrostatic potentials. The direction of the field lines indicates the direction of attractive electrostatic interactions. Only electric field lines with a gradient magnitude above 2.23 kTe^{-1}Å^{-1} are shown. Active site residues are marked and represented with sticks.

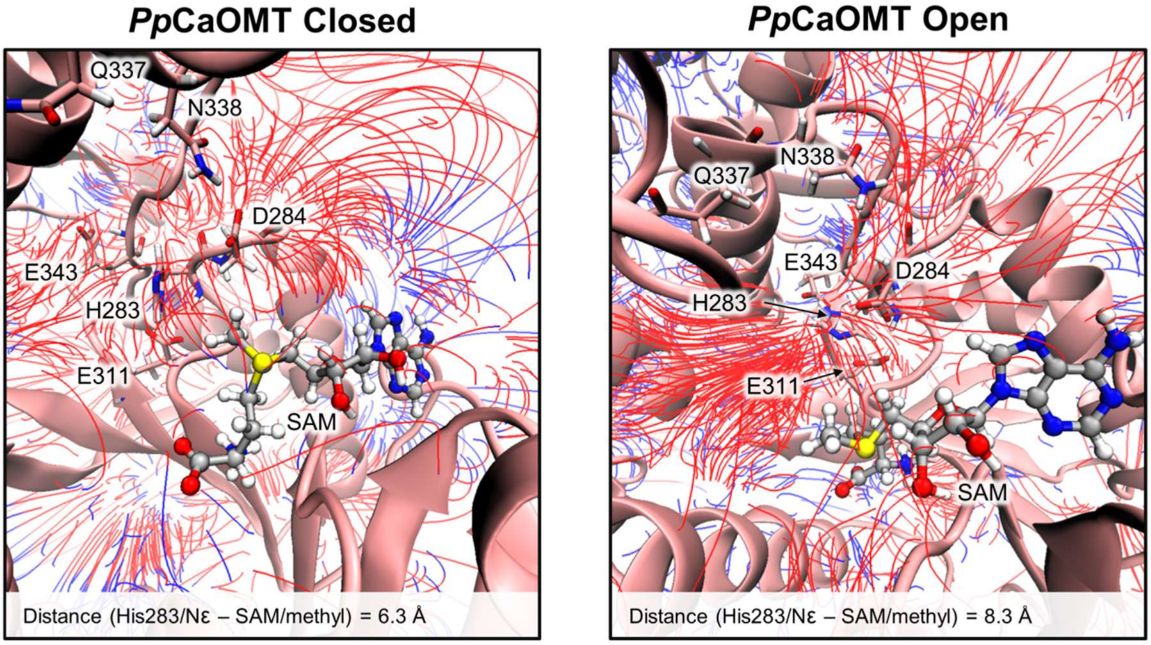

Figure S52 – Map of the electric field in the active site of the open and closed conformational states of *Pp*CaOMT. The open conformation is obtained from the same representative frame as the closed conformation. Therefore, the two displayed catalytic cavities correspond to the different chains in the homodimer corresponding to the same MD frame. The red-coloured line segments indicate negative electrostatic potentials, while blue segments represent positive electrostatic potentials. The direction of the field lines indicates the direction of attractive electrostatic interactions. Only electric field lines with a gradient magnitude above 2.23 $kTe^{-1}Å^{-1}$ are shown. Active site residues are marked and represented with sticks. The results show that the electrostatic environment remains qualitatively unchanged when comparing closed and open conformations.

Hydrogen/deuterium exchange – mass spectrometry

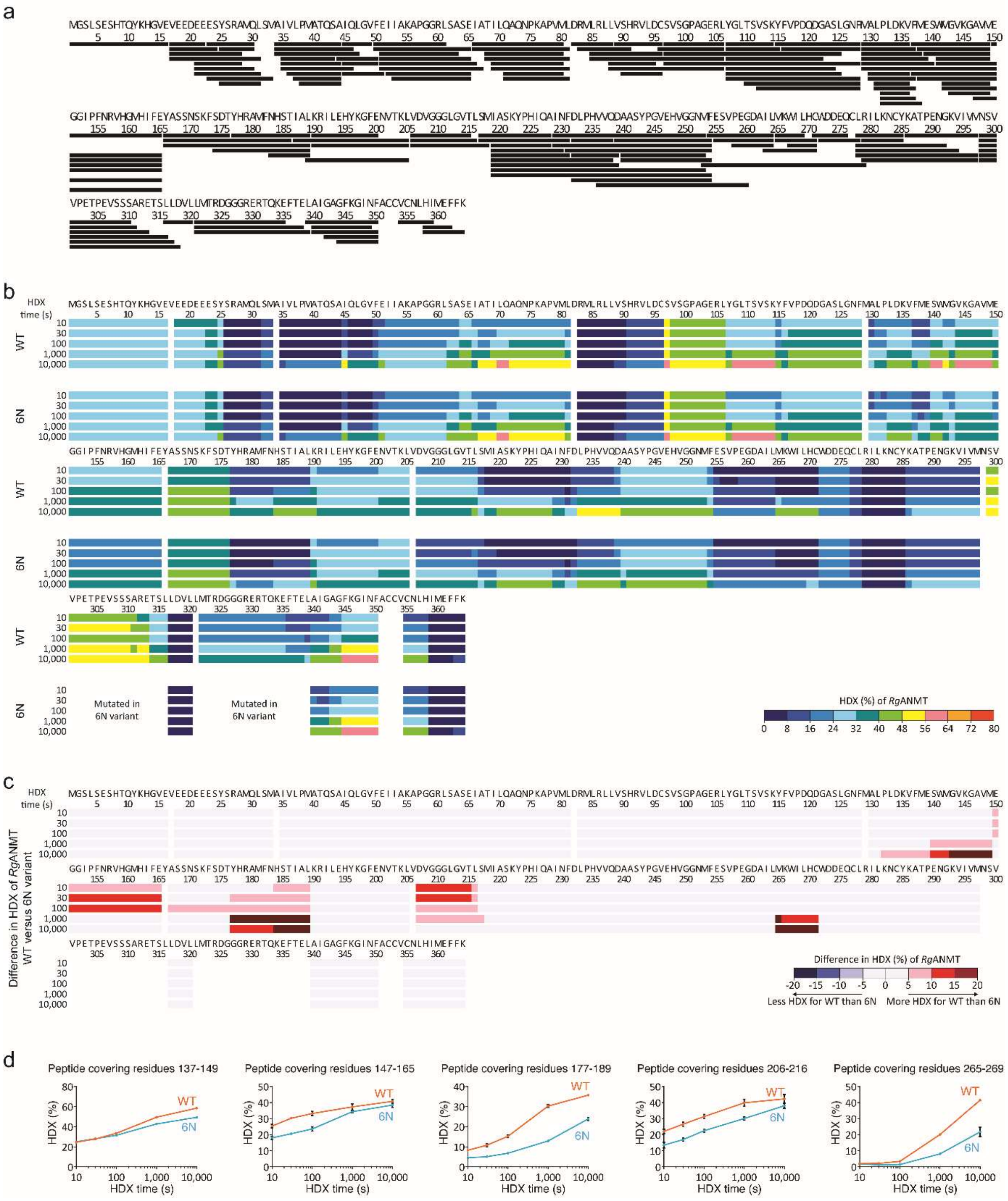

Figure S53 – Differences in protein conformation between *Rg*ANMT WT and its 6N variant probed by HDX-MS. a. Each black bar denotes a *Rg*ANMT peptide identified in HDX-MS experiments. b. Residue-specific HDX of *Rg*ANMT WT and 6N variant derived from peptide's HDX analysis. c. Difference in residue-specific HDX of *Rg*ANMT between WT and the 6N variant. Red colour indicates higher observed HDX for *Rg*ANMT WT than for 6N variant. d. HDX of selected representative *Rg*ANMT peptides. Data represent the mean ± s.d. of n = 3 replicates.

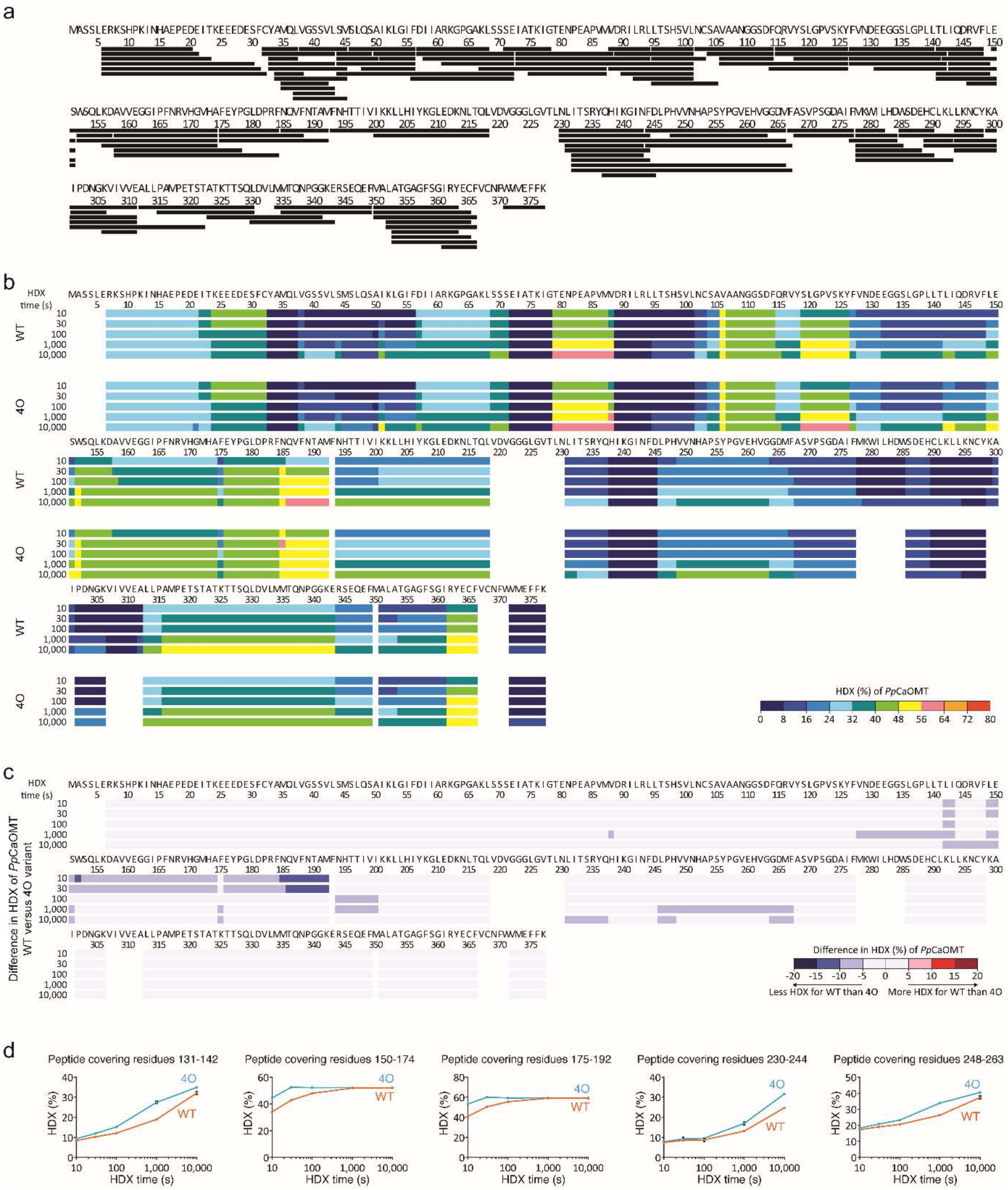

Figure S54 – Differences in protein conformation between *Pp*CaOMT WT and its 4O variant probed by HDX-MS. a. Each black bar denotes a *Pp*CaOMT peptide identified in HDX-MS experiments. b. Residue-specific HDX of *Pp*CaOMT WT and 4O variant derived from peptide's HDX analysis. c. Difference in residue-specific HDX of *Pp*CaOMT between WT and the 4O variant. Blue colour indicates lower observed HDX for *Pp*CaOMT WT than for 4O variant. d. HDX of selected representative *Pp*CaOMT peptides. Data represent the mean ± s.d. of n = 3 replicates.

Supporting References

(1) Sievers, F.; Higgins, D. G. Clustal Omega for Making Accurate Alignments of Many Protein Sequences. *Protein Sci.* **2018**, *27* (1), 135–145. https://doi.org/10.1002/pro.3290.

(2) Sievers, F.; Wilm, A.; Dineen, D.; Gibson, T. J.; Karplus, K.; Li, W.; Lopez, R.; McWilliam, H.; Remmert, M.; Söding, J.; Thompson, J. D.; Higgins, D. G. Fast, Scalable Generation of High-quality Protein Multiple Sequence Alignments Using Clustal Omega. *Mol. Syst. Biol.* **2011**, *7* (1). https://doi.org/10.1038/msb.2011.75.

(3) Jockmann, E., Subrizi, F., Mohr, M.K.F., Carter, E.M., Hebecker, P.M., Popadić, D., Hailes, H.C., Andexer, J.N., 2023. Expanding the Substrate Scope of N- and O-Methyltransferases from Plants for Chemoselective Alkylation**. *ChemCatChem* **2023**, *15* (22), e202300930. https://doi.org/10.1002/cctc.202300930.
